# Improved immunostaining of nanostructures and cells in human brain specimens through expansion-mediated protein decrowding

**DOI:** 10.1126/scitranslmed.abo0049

**Published:** 2024-01-31

**Authors:** Pablo A. Valdes, Chih-Chieh (Jay) Yu, Jenna Aronson, Debarati Ghosh, Yongxin Zhao, Bobae An, Joshua D. Bernstock, Deepak Bhere, Michelle M. Felicella, Mariano S. Viapiano, Khalid Shah, E. Antonio Chiocca, Edward S. Boyden

**Affiliations:** 1 Department of Neurosurgery, University of Texas Medical Branch, Galveston, TX, 77555; 2 Department of Neurosurgery, Brigham and Women’s Hospital, Harvard Medical School, Boston, MA, USA, 02115; 3 Media Arts and Sciences, MIT, Cambridge, MA, USA, 02115; 4 Department of Biological Engineering, MIT, MA, USA, 02139; 5 McGovern Institute for Brain Research, MIT, Cambridge, MA, USA, 02139; 6 RIKEN Center for Brain Science, Saitama, Japan, 351-0198; 7 Department of Brain and Cognitive Sciences, MIT, Cambridge, MA, USA, 02139; 8 Department of Biological Sciences, Carnegie Mellon University, Pittsburgh, PA, USA, 15213; 9 Koch Institute, MIT, Cambridge, MA, USA, 02139; 10 Department of Pathology, Microbiology and Immunology, School of Medicine Columbia, University of South Carolina, Columbia, SC, USA, 29209; 11 Center for Stem Cell and Translational Immunotherapy, Harvard Medical School/Brigham and Women’s Hospital, Boston, MA, USA, 02115; 12 Department of Pathology, University of Texas Medical Branch, Galveston, TX, USA, 77555; 13 Department of Neuroscience and Physiology, SUNY Upstate Medical University, Syracuse, NY, USA, 13210; 14 MIT Center for Neurobiological Engineering and K. Lisa Yang Center for Bionics, MIT, Cambridge, MA, USA, 02139; 15 Howard Hughes Medical Institute, Cambridge, MA, USA, 02139

## Abstract

Proteins are densely packed in cells and tissues, where they form complex nanostructures. Expansion microscopy (ExM) variants have been used to separate proteins from each other in preserved biospecimens, improving antibody access to epitopes. Here we present an ExM variant, decrowding expansion pathology (dExPath), which can expand proteins away from each other in human brain pathology specimens, including formalin-fixed paraffin-embedded (FFPE) clinical specimens. Immunostaining of dExPath-expanded specimens reveals, with nanoscale precision, previously unobserved cellular structures, as well as more continuous patterns of staining. This enhanced molecular staining results in observation of previously invisible disease marker-positive cell populations in human glioma specimens, with potential implications for tumor aggressiveness. dExPath results in improved fluorescence signals even as it eliminates lipofuscin-associated autofluorescence. Thus, this form of expansion-mediated protein decrowding may, through improved epitope access for antibodies, render immunohistochemistry more powerful in clinical science and, perhaps, diagnosis.

## One- sentence summary

Tissue expansion and subsequent labeling of decrowded proteins reveals previously unobserved structures and cells in human brain pathology specimens.

## Introduction

Immunohistochemistry uses antibodies to identify accessible epitopes on proteins embedded in intact cells and tissues. Target epitopes in fixed tissues are often physically inaccessible to conventional antibodies (3–14), such as the commonly used class of immunoglobulin G (IgG) (1, 2).

Expansion microscopy (ExM) enables physical expansion of biological specimens, thereby permitting nanoscale resolution imaging on diffraction-limited microscopes (15, 16). Briefly, ExM starts by covalently anchoring biomolecules, or labels against biomolecules, to a swellable hydrogel densely and evenly synthesized throughout a preserved biological specimen. Then, an enzymatic or protein-denaturing treatment softens the mechanical properties of the specimen. Water then causes the polymer network to expand, and thus the anchored molecules to be pulled uniformly away from one another. Given the difficulty of labeling many epitopes in their natural, densely packed state, we asked whether, in human tissues, conventional antibodies introduced in the post-expansion, decrowded, state could access previously undetectable epitopes.

Some expansion protocols preserve protein antigens throughout the expansion process (Table S1) (17–25), and are thus compatible with post-expansion immunostaining. However, most of these existing post-expansion staining protocols either require specialized fixative compositions (17, 18, 21, 22, 25), and thus are incompatible with archival clinical samples, or cause tissue cracks and anisotropy due to incomplete tissue softening (19), or had uncharacterized nanoscale isotropy (20). In addition, none of these studies underwent quantitative comparison of structures or cells in the same specimen of human tissue with pre- versus post- expansion staining, key to understanding whether the decrowding of proteins contributed to visualization of previously invisible structures.

We previously developed expansion pathology (ExPath), a form of ExM that prepares human specimens for expansion microscopy, using pre-expansion antibody staining (6). Here we present decrowding ExPath (dExPath), an expansion pathology variant that preserves protein epitopes for post-expansion staining. dExPath can be applied to formalin-fixed paraffin-embedded (FFPE) human tissues, as well as other standard formats of interest in basic and applied biology, such as4%-paraformaldehyde (PFA)-fixed mouse brain tissue. We validated dExPath systematically, comparing, within the same specimen of human brain tissue, immunostaining intensity and continuity between pre- and post-expansion staining, showing improvements in both intensity and continuity, and revealing new features, including disease marker-bearing cell populations (in human glioma specimens) that were previously invisible. Furthermore, dExPath eliminates autofluorescence associated with lipofuscin, an aggregated product commonly found in brain tissue, in addition to autofluorescence reduction resulting from the loss of autofluorescent molecules shown in prior expansion protocols (6). dExPath supports multi-round immunostaining, enabling highly multiplexed imaging of protein targets within the same human brain specimen.

## Results

### Rationale for dExPath technology

**Fig. 1.**
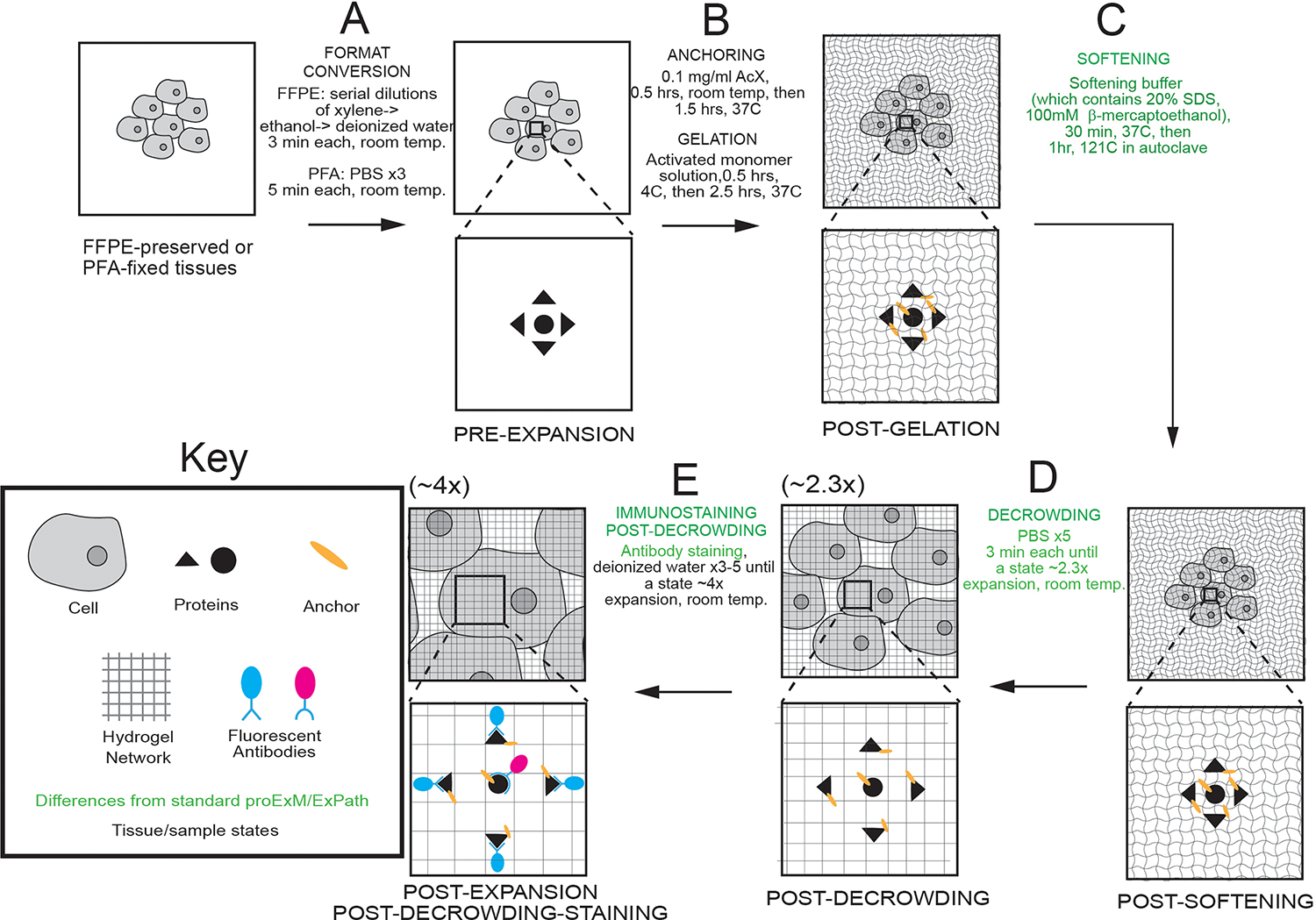
Decrowding expansion pathology (dExPath) for post-expansion immunostaining of human tissue and other formaldehyde-fixed specimens. **(A-E)** Workflow for expanding formalin-fixed paraffin-embedded (FFPE) or formaldehyde-fixed human or mouse brain specimens. Key modifications of proExM/ExPath protocols are highlighted in green. PFA, paraformaldehyde; PBS, phosphate buffered saline; RT, room temperature; AcX, Acryloyl-X; SDS, sodium dodecyl sulfate. (A) Tissue samples undergo conversion into a state compatible with expansion. (B) Tissue samples are treated so that gel-anchorable groups are attached to proteins, then the sample is permeated with an expandable polyacrylate hydrogel. (C) Samples are incubated in a softening buffer to denature, and loosen disulfide bonds and fixation crosslinks between, proteins. (D) Softened samples are washed in a buffer to partially expand them. Linear expansion factor is shown in parentheses. (E) Samples are stained and then expanded fully by immersion in water.

We first prepared tissue to enter the expansion pipeline (Fig. 1A; involving tissue deparaffinization and re-hydration, for FFPE samples) (6), followed by protein anchoring and gel formation (Fig. 1B). In contrast to the original ExPath protocol, which uses protease digestion to soften the specimen (feasible because fluorescent antibodies, which are partly protease-resistant, are applied pre-expansion and anchored to the polymer network for later imaging), we used a buffer to maximally enable protein separation for post-expansion staining. We used higher concentrations of sodium dodecyl sulfate (SDS) (20% weight/volume (w/v)) than in earlier protein-preserving protocols (Table S1) (17–23, 26), reasoning that this could better denature proteins, and minimize non-covalent intra- and inter-protein interactions (27, 28). We included a new ingredient, the reducing agent β-mercaptoethanol (100 mM), which we reasoned could cleave inter-molecular disulfide bridges between proteins (24, 27–31). We used the same high concentration of ethylenediaminetetraacetic acid (EDTA) (25 mM) as in original ExPath, which proved to be useful for isotropic tissue expansion (6), possibly through de-stabilization of metal-mediated protein interactions (28–30). We used a higher temperature than in original ExPath, adapted from a form of proExM that uses autoclaving to expose samples to 121°C (Fig. 1C) to strongly denature and loosen bonds between proteins in the sample, allowing them to separate during washes (which drives partial tissue expansion, ~2.3x; Fig. 1D). Antibodies were applied at this post-decrowding state (Fig. 1E; see Table S2 for antibodies used in this work), instead of the fully expanded [~4x] state, because full expansion requires sample immersion in water, which can hinder antibody binding(17–22). Multiplexing is possible because these antibodies can be stripped using the same buffer, and then new antibodies applied (fig. S1), a strategy previously demonstrated by other post-expansion staining protocols but not on human tissues (18, 20, 32).

High grade glioma tissues are known to undergo abnormal endothelial proliferation, leading to some areas of tissue with abnormally large amounts of vascularity and extracellular matrix (ECM). These areas can be identified under conventional clinical microscopy (33) but present a challenge to isotropic expansion of tissue (6). To circumvent this problem, we devised a modified form of dExPath using collagenase treatment prior to softening (fig. S2). Thus, dExPath was designed to provide a methodology for isotropic tissue expansion, enabling preservation, and post-expansion as well as multiplexed staining, of decrowded proteins in both normal and pathologic human and rodent brain tissues.

### Validation of dExPath expansion isotropy in brain tissue

**Fig. 2.**
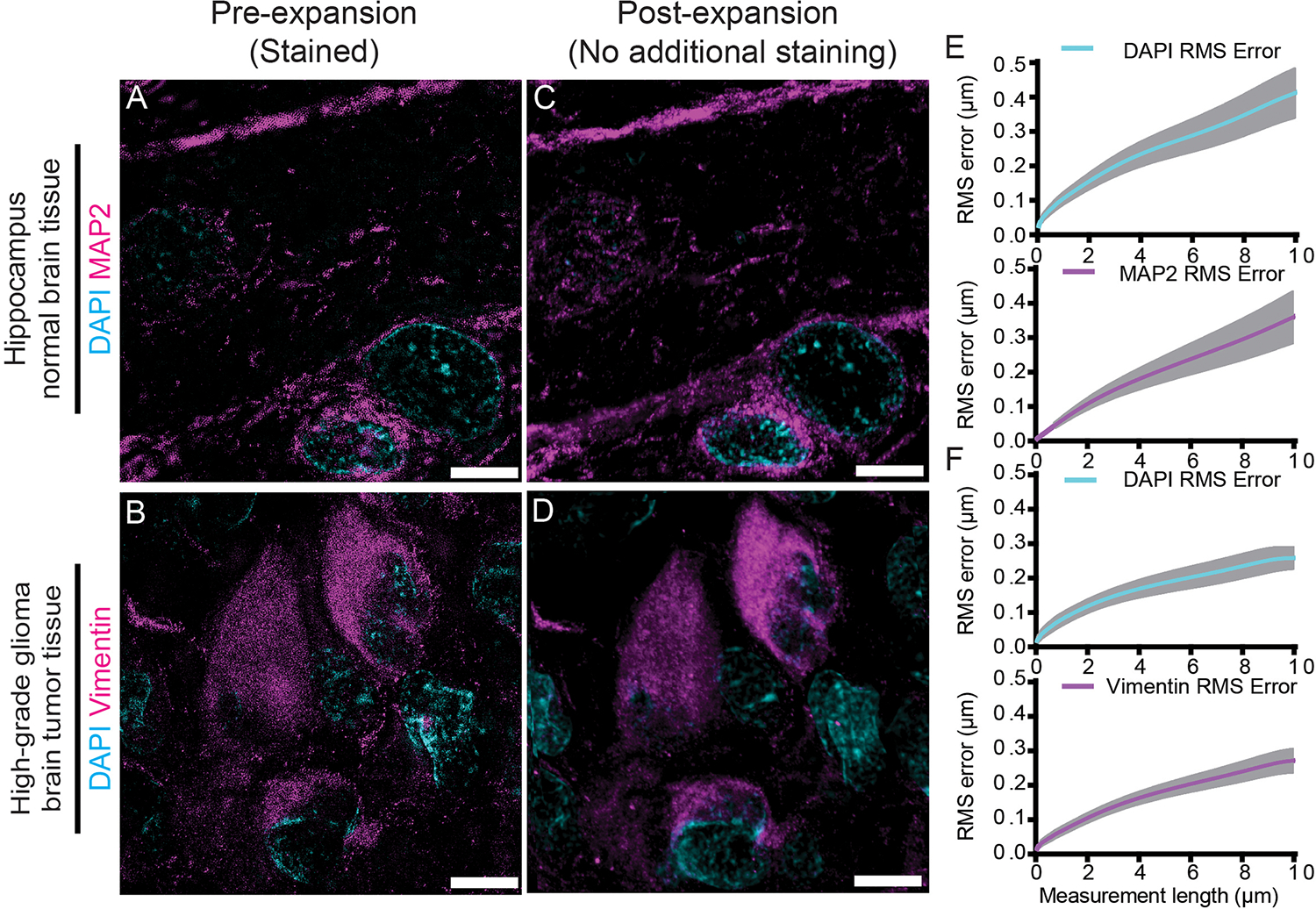
Isotropy of dExPath. **(A-B)** Representative pre-expansion super resolution structured illumination microscopy (SR-SIM) images of healthy human hippocampus (A) and human cerebrum high-grade glioma brain tumor tissue (B) which underwent processing as in fig. S3A with staining for MAP2 and DAPI (A), or for vimentin and DAPI (B). **(C-D)** Post-expansion images of the same fields of view as in (A-B), respectively. Samples underwent anchoring, gelation and softening (as in fig. S3B–C), another round of DAPI staining, ~4x linear expansion (as in fig. S3D), and imaging with confocal microscopy. **(E-F)** Root-mean-square (RMS) length measurement errors obtained by comparing pre- and post-expansion images such as in A-D (n = 4 samples, each from a different patient, E; n = 3 samples, each from a different patient, F). Line, mean; shaded area, standard deviation. Images are sum intensity z-projections, either of SR-SIM (A-B), or confocal (C-D) image stacks, both covering an equivalent tissue depth in biological units. Brightness and contrast settings: first set by the ImageJ auto-scaling function, and then manually adjusted to improve contrast for the stained structures of interest; quantitative analysis in (E-F) was conducted on raw image data. Scale bars (in biological units: physical sizes of expanded samples divided by their expansion factors, used throughout this manuscript, unless otherwise noted): (A-D) 5 μm. Linear expansion factors: (C-D) 4.0x.

We validated the isotropy of dExPath on normal and diseased FFPE 5-μm-thick human brain tissues (standard for clinical samples), using the pre-vs-post distortion analysis used to validate earlier expansion protocols (6, 15, 18, 19, 34, 35). We performed antigen retrieval followed by pre-expansion immunostaining against microtubule-associated protein 2 (MAP2, a neuronal dendritic marker) (36), and the intermediate filament protein vimentin (37–39), on normal human hippocampus (Fig. 2A) and on high-grade glioma tissues (located in the human cortex or white matter) (Fig. 2B), respectively. We performed standard immunostaining (6, 40–42) and obtained pre-expansion images using a super-resolution structured illumination microscope (SR-SIM) (Fig. 2A–B). Next, we performed dExPath (modified to use pre-expansion staining prior to anchoring and gelation, to facilitate distortion comparison between pre- and post-expansion images of the same sample, outlined in fig. S3), obtaining post-expansion images of the same fields of view (Fig. 2C–D) using a confocal microscope. We observed low distortion between pre- and post-expansion images, similar to previous versions of ExM applied to mouse brain tissue (6, 15, 19) (Fig. 2E–F). Our modified form of dExPath using collagenase treatment prior to softening was used to compare pre- and post-expansion images of the same specimen, outlined in fig. S4; low distortion was obtained on high-grade glioma tissues with a high degree of ECM (fig. S5). Thus, dExPath isotropically expands archival clinical samples of FFPE normal brain and brain tumor tissues by ~4x without the need for enzymatic epitope destruction (6, 19), or specialized fixatives (17, 18, 21, 22).

### dExPath removes lipofuscin autofluorescence, improving visualization of intracellular structures

**Fig. 3.**
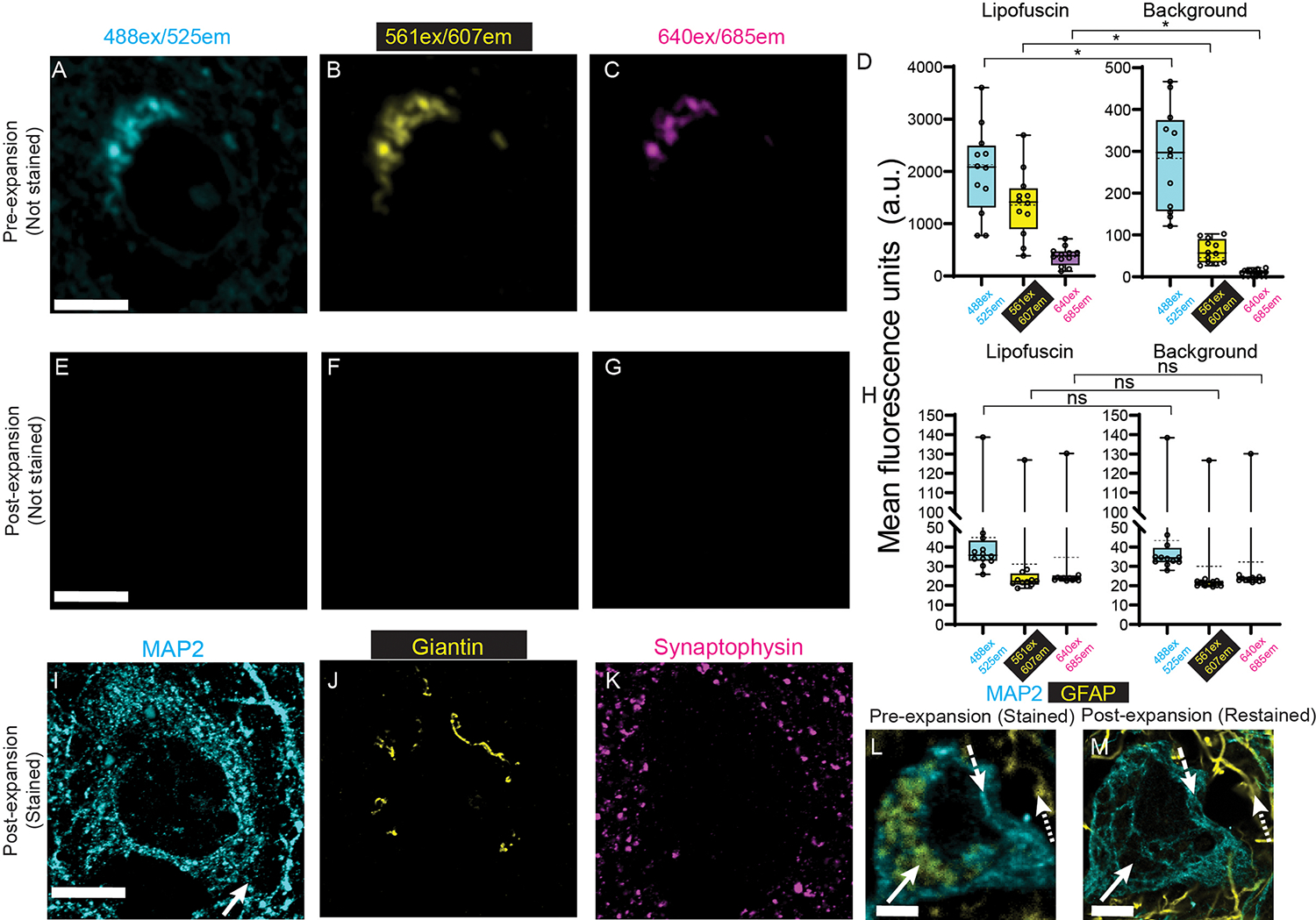
dExPath removal of lipofuscin autofluorescence. **(A-C)** Pre-expansion confocal images (single z slices) of a neuron in a a 5-μm-thick normal human cortex sample(format conversion as in Fig. 1A). Images were acquired for 3 fluorescent channel settings: (A) 488 nm ex/ 525 nm em; (B )561ex/607em; and (C) 640ex/685em. **(D)** Mean fluorescence intensities from pre-expansion images, averaged across regions of interest (ROIs) that exhibited prominent lipofuscin (left bar graph), as well as across background ROIs (right bar graph); (n = 4 tissue samples, each from a different patient). Brightness and contrast settings: first set by the ImageJ auto-scaling function, and then manually adjusted to improve contrast for lipofuscin; quantitative analysis was conducted on raw image data. Box plot: individual values (open circles; 3 measurements were acquired from each patient), median (middle line), mean (dotted line), first and third quartiles (lower and upper box boundaries), lower and upper raw values (whiskers). Statistical testing: 2-tailed paired t-test (non-Bonferroni corrected) was applied to lipofuscin vs. background, for pre-expansion mean fluorescence intensities for each spectral channel. *, p < 0.05; ns, not significant. **(E-G)** Shown are post-expansion confocal images after the sample from A-C was treated with anchoring, gelation,softening and decrowding (as in Fig. 1B–D), DAPI staining, and ~4x linear expansion, without post-decrowding immunostaining. Sum intensity z-projections of image stacks corresponding to the biological thickness of the original slice, taken under identical settings and of the same field of view as A-C and displayed under the same settings. **(H)** Mean fluorescence intensities, from post-expansion images, averaged across the same lipofuscin (left) and background (right) ROIs used in panel D. Plots and statistics as in D. **(I-K)** Confocal images as in (E-G), after post-decrowding immunostaining for MAP2 (microtubule-associated protein 2), giantin, and synaptophysin (labeled with antibodies in the same spectral ranges as indicated above A-C), as well as stained for DAPI (not shown; used for alignment), and then re-expanded to ~4x linear expansion. **(L)** Representative pre-expansion confocal image of a tissue sample of FFPE 5-μm-thick normal human hippocampus processed as in fig S3A. Pre-expansion immunostaining for MAP2 (488ex/525em) and GFAP (glial fibrillary acidic protein) (640ex/685em). Solid arrow indicates a region with lipofuscin aggregates (GFAP-like staining but found in a neuron); dashed arrow indicates MAP2 staining without lipofuscin; dotted arrow indicates GFAP staining. **(M)** Confocal image of the same field of view as (L). Tissues underwent softening and ~4x expansion, followed by decrowding, post-decrowding staining for MAP2 and GFAP, and expansion to ~4x (as in fig. S3B–F). Arrows, as in L. Scale bars (in biological units): (A, E, I) 7 μm; (L, M) 5 μm. Linear expansion factors: (E-G, I-K) 4.3x; (M) 4.1x.

Fluorescence microscopy of clinical tissues is often hindered by lipofuscin (43–50), an autofluorescent (throughout the visible optical spectrum) material that is composed of aggregates of oxidized proteins, lipids, and metal cations, and that accumulates in many cell and tissue types (51–54). We imaged regions with lipofuscin in normal human cortex (age: 19 – 45 years old), in the pre-expansion state (Fig. 3A–D) and in the post-expansion state (Fig. 3E–H), under 3 common fluorescent channel settings (488 nm excitation/525 nm emission; 561ex/607em; 640ex/685em), finding that lipofuscin fluorescence was at least an order of magnitude higher than background fluorescence (Fig. 3D; lipofuscin vs background: 488ex/525em, p = 0.00001; 561ex/607em, p = 0.00002; 640ex/685em, p = 0.00002; 2-tailed paired t-test; all t-tests were non-Bonferroni corrected;). After dExPath, lipofuscin autofluorescence was reduced to background brightness (Fig. 3H; lipofuscin vs background: 488ex/525em, p = 0.11; 561ex/607em, p = 0.07; 640ex/685em, p = 0.29; 2-tailed paired t-test). dExPath removed lipofuscin autofluorescence in brain tissue specimens from patients with Alzheimer’s disease (AD) (Fig. S6). Classical ExPath showed some lipofuscin autofluorescence post-expansion (fig. S7). Using dExPath, structures masked by lipofuscin became detectable. Comparing the same location in the same specimen pre- and post-expansion, with stains against MAP2 (36), giantin (a Golgi-apparatus marker) (55, 56), and synaptophysin (a pre-synaptic marker) (57) (Fig. 3I–K), some giantin staining overlapped with lipofuscin (compare Fig. 3B vs. 3J, note, images were obtained with the same microscope settings). As another example, human hippocampal tissues that underwent pre-expansion immunostaining against MAP2 (488ex/525em) and glial fibrillary acidic protein (GFAP, a marker of astrocytes (37, 58, 59); 640ex/685em) showed false positive fluorescence in the GFAP channel in somata of MAP2-positive cells (Fig. 3L). In contrast, post-decrowding, such false positive GFAP staining no longer appeared in the somata (Fig. 3M). Thus, dExPath-mediated lipofuscin removal has the potential to improve detection of fluorescent signals in human tissues.

### dExPath enables visualization of decrowded proteins revealing previously invisible cells and structures

**Fig. 4.**
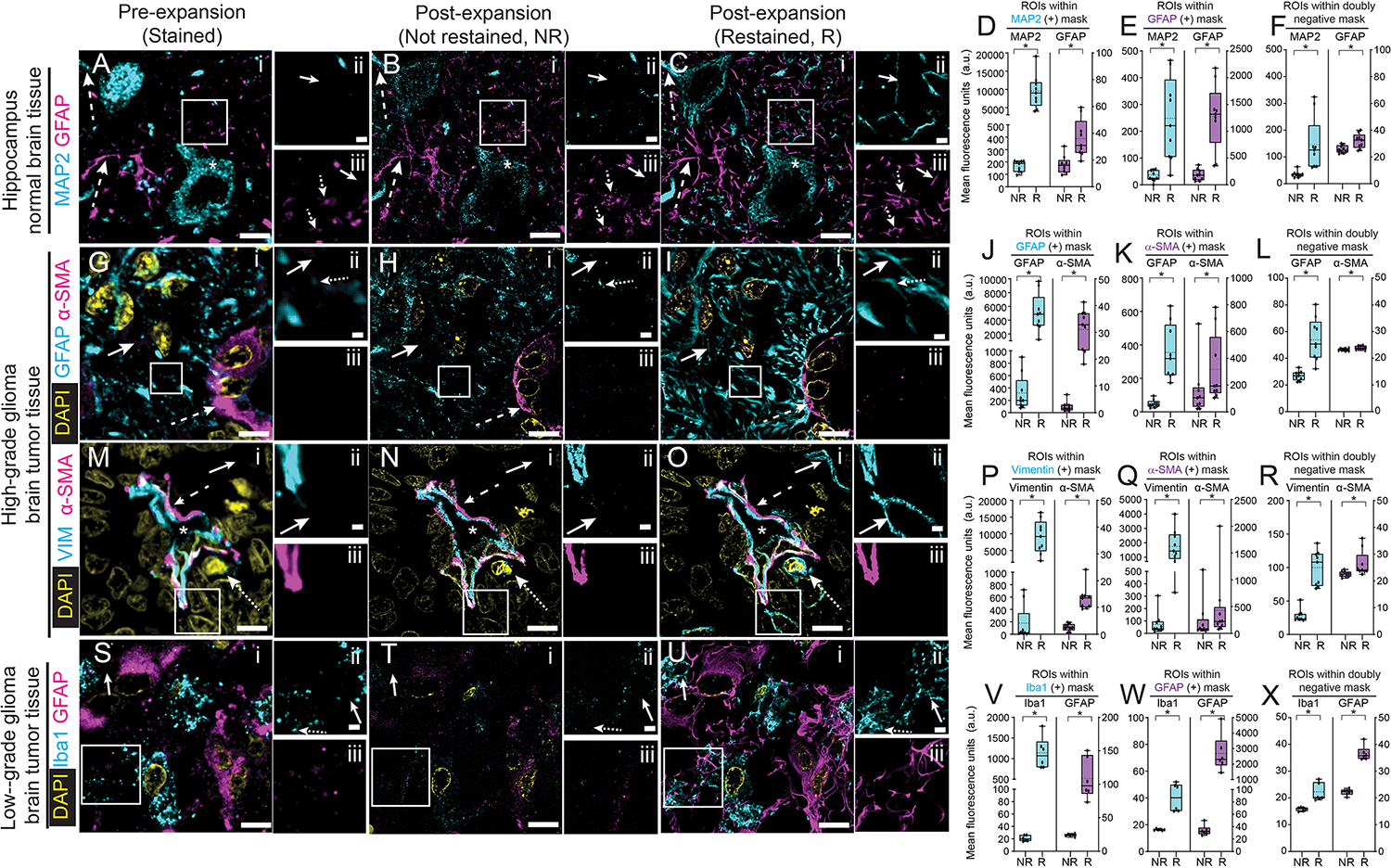
dExPath-mediated protein decrowding reveals cells and structure not detected in pre-expansion staining forms of expansion microscopy. **(A)** Representative pre-expansion confocal image (single z slice) of 5-μm-thick FFPE normal human hippocampal tissue.(Sample underwent processing as in fig. S3A and immunostaining for MAP2 and GFAP. White box in (i) marks a region with sparse and discontinuous signals that is shown magnified and in separate channels at the right (MAP2 in (ii) and GFAP in (iii)). MAP2 staining of a putative cell body (asterisk in (i)) and dendrite (upper dashed arrow in (i)). GFAP staining of a putative astrocytic process (lower dashed arrow in (i)) and discontinuous GFAP regions (dotted arrows in (iii)). Solid arrows show regions that were MAP2-negative (ii) or GFAP-negative (iii) in pre-expansion images (A), for comparison to post-expansion staining panels later in this figure. **(B)** Shown is a post-expansion confocal image after processing as in fig. S3B–D and imaging at ~4x linear expansion. Sum intensity z-projection of an image stack covering the biological thickness of the original slice (used for all expanded images throughout this figure); images were of the same fields of view as in (A), using identical hardware settings. Asterisks and arrows as in (A). **(C)** Post-decrowding stained confocal images of the same fields of view as in (A-B) after decrowding and additional immunostaining for MAP2 and GFAP and re-expansion to ~4x (fig. S3E–F), using identical hardware settings. Asterisks and arrows as in (A). **(D)** Quantification of fluorescence intensities for raw data of images post-expansion such as those of B (NR, “not restained”) and C (R, “restained”), averaged across MAP2-positive ROIs, for the MAP2 channel (cyan) and the GFAP channel (magenta). Box plot: individual values (open circles; 3 measurements were acquired from each patient), median (middle line), mean (dotted line), first and third quartiles (lower and upper box boundaries), lower and upper raw values (whiskers). Statistical testing: 2-tailed paired t-test (non-Bonferroni corrected) *, p < 0.05. **(E)** As in D, but for GFAP-positive ROIs, for the MAP2 channel (cyan) and the GFAP channel (magenta). **(F)** As in D, but for double negative ROIs, for the MAP2 channel (cyan) and the GFAP channel (magenta). **(G)** Representative pre-expansion confocal image (single z slice) of 5-μm-thick FFPE human high-grade glioma. Sample underwent format conversion, antigen retrieval, and immunostaining for GFAP and α-SMA (α-smooth muscle actin), and DAPI staining (fig. S3A). White box in (i) marks a region with sparse and discontinuous signals that is shown magnified and in separate channels at the right (GFAP in (ii) and α-SMA in (iii)). α-SMA-staining of pericytes that are enveloping blood vessels (dashed arrow in (i)). Discontinuous GFAP regions (dotted arrow in (ii)). Solid arrows in (i) and (ii) show regions that were GFAP-negative pre-expansion (G), for comparisons to post-expansion staining panels later in this figure. **(H)** As in B, but for panel G. **(I)** As in C, but for panel G. **(J)** As in D, but for the GFAP (cyan) and α-SMA (magenta) channels, in GFAP-positive ROIs. **(K)** As in D, but for the GFAP (cyan) and α-SMA (magenta) channels in α-SMA-positive ROIs. **(L)** As in D, but for the GFAP (cyan) and α-SMA (magenta) channels in double negative ROIs. **(M)** Representative (pre-expansion confocal image (single z slice) of 5-μm-thick human high grade glioma tissue (cortex or white matter). Sample underwent format conversion, antigen retrieval, and immunostaining for vimentin and α-SMA, and DAPI staining (fig. S3A). White box in (i) marks a region including part of a blood vessel that is shown magnified and in separate channels to the right (vimentin in (ii) and α-SMA in (iii)). Vimentin and α-SMA-staining of the blood vessel wall (dashed arrow in (i)) which surrounds the vessel lumen (asterisk in (i)). A vimentin-positive cell outside the blood vessel (dotted arrow in (i)). Solid arrows in (i) and (ii) show regions that were vimentin-negative pre-expansion (M), for comparison to post-expansion staining panels later in this figure. **(N)** As in B, but for panel M. **(O)** As in C, but for panel M. **(P)** As in D, but for the vimentin channel (cyan) and the α-SMA channel (magenta), in vimentin-positive ROIs. **(Q)** As in D, but for the vimentin channel (cyan) and the α-SMA channel (magenta), in α-SMA-positive ROIs. **(R)** As in D, but for the vimentin channel (cyan) and the α-SMA channel (magenta), in double negative ROIs. **(S)** Representative pre-expansion confocal image (single z slice) of 5-μm-thick human low grade glioma tissue (cortex or white matter). Sample underwent format conversion, antigen retrieval, and immunostaining for ionized calcium binding adapter molecule 1 (Iba1) and GFAP, and DAPI staining (fig. S3A). White box in (i) marks a region with sparse and discontinuous signals that is shown magnified and in separate channels to the right (Iba1 in (ii) and GFAP in (iii)). Iba1 staining of discontinuous regions (dotted arrow in (ii)). Solid arrows in (i) and (ii) show regions that were Iba1-negative pre-expansion (S), for comparison to post-expansion staining panels later in this figure. **(T)** As in B, but for panel S. **(U)** As in C, but for panel S. **(V)** As in D, but for the Iba1 channel (cyan) and the GFAP channel (magenta), in the Iba1-positive ROIs. **(W)** As in D, but for the Iba1 channel (cyan) and the GFAP channel (magenta), in GFAP-positive ROIs. **(X)** As in D, but for the Iba1 channel (cyan) and the GFAP channel (magenta), in the double negative ROIs. Scale bars: (A-C) panel i, 9 μm; ii, 1.7 μm; (G-I) i, 7 μm; ii, 0.7 μm; (M-O) i, 8 μm; ii, 0.8 μm; (S-U) i, 8 μm; ii, 0.8 μm. Linear expansion factors: (B,C) 4.1x; (H,I) 4.0x; (N,O) 4.3x; (T,U) 4.2x.

To investigate whether post-expansion immunostaining could enable detection of previously inaccessible protein epitopes, we compared pre- vs. post-expansion staining of normal human hippocampus (Fig. 4A–F), supratentorial high-grade glioma tumor specimens (Fig. 4G–R), and low-grade glioma tumor specimens (Fig. 4S–X). Tissue samples were imaged pre-expansion, after antigen retrieval and antibody staining (Fig. 4A, G, M, S), and after expansion without restaining (Fig. 4B, H, N, T); and after expansion and re-staining with the same antibodies (Fig. 4C, I, O, U; experimental pipeline in Fig. S3). All tissue states were imaged using identical confocal imaging settings.

In one experiment (Fig. 4A–C), we used antibodies against the somato-dendritic marker, MAP2 (36, 60) and the astrocytic marker, GFAP (37, 58, 59, 61, 62). MAP2 staining yielded putative cell bodies and dendrites as well as sparser discontinuous dendrite-like regions (Fig. 4A). The latter regions remained discontinuous after 4x expansion (Fig. 4B). However, after post-expansion re-staining, new filaments appeared in areas previously MAP2-negative (Fig. 4C). We found similar improvements for GFAP, with pre-expansion staining showing discontinuous signals (Fig. 4A). Post-expansion, resolution improved (Fig. 4B), and after re-staining, those regions appeared more continuous, and new GFAP fibers became visible (Fig. 4C).

To quantify the improvement in labeling post-expansion vs. pre-expansion, we constructed a binary image “signal” mask, for each stain, that corresponded to pixels that were positive (above a manually selected threshold) for a given stain in both pre-expansion and post-expansion staining images. We also created a second “background” mask, for each stain, that corresponded to pixels that were negative (below the threshold mentioned before) in both pre- and post-expansion staining images; a “double negative” background mask corresponded to the pixels that were negative in both of these background masks. Next, we constructed regions of interest (ROIs) that were small enough (0.2 microns) to fit entirely within the signal mask for a given stain, but that were at least an ROI-width away from the signal mask for the other stain; we also constructed ROIs that were fully contained within the double negative mask, and similarly far from pixels that were positive in either signal mask. Finally, we calculated intensities averaged across the ROIs for the same locations in the expanded (Fig. 4B) vs. expanded-and-restained (Fig. 4C) images. In regions positive in the MAP2 and GFAP signal masks (Fig. 4D, left and Fig. 4E, right), we saw increases of both signals in their respective ROIs (MAP2, p = 0.0003, 2-tailed paired t-test; n = 3 tissue samples from different patients; GFAP, p = 0.0007, 2-tailed paired t-test; n = 3 tissue samples from different patients). Of course, we would not expect MAP2 to occur in GFAP-positive regions, nor GFAP in MAP2 regions. Thus, these two proteins give us the opportunity to assess whether post-expansion antibody application suffers from nonspecific staining. Indeed, GFAP was consistently low in both pre-expansion and post-expansion images (p = 0.0004, 2-tailed paired t-test; n = 3 tissue samples from different patients) in locations within the MAP2 signal mask. Similarly, MAP2, imaged in the GFAP signal mask, was consistently low in pre- and post-expansion images (p = 0.003, 2-tailed paired t-test).

In the double negative regions, MAP2 intensities were consistently low in pre-decrowding and post-decrowding states (Fig. 4F, left), as were GFAP intensities (Fig. 4F, right). Thus, staining in the double negative regions was similar to that in the single negative regions, supporting the idea that the nonspecific staining is extremely low.

We performed a similar analysis in high-grade glioma tissue from a human patient (Fig. 4G–I), staining for GFAP, which in glioma patients marks both astrocytes and glioma cells (58, 63–65), and α-SMA, a marker of pericytes (66–68), which envelope blood vessels (Fig. 4G). As with MAP2 vs. GFAP, α-SMA and GFAP would not be expected to overlap, except perhaps at sites where astrocytes and glioma cells touch pericytes (68, 69); accordingly, we chose GFAP-positive and α-SMA-positive ROIs that were far apart from α-SMA and GFAP staining respectively, as well as double negative ROIs that exhibited neither. As before, GFAP became more continuous with post-expansion staining (Figs. 4G–I), showing new filaments, and an overall increase in intensity in GFAP-positive ROIs (Fig. 4J, left; p = 0.0006, 2-tailed paired t-test; n = 3 tissue samples from different patients). α-SMA intensity also went up in α-SMA-positive regions (Fig. 4K, right, p = 0.0006 2-tailed paired t-test; n = 3 tissue samples from different patients). In contrast, α-SMA was consistently low in pre- and post-expansion states, in GFAP-positive ROIs (Fig. 4J, right; p = 0.004, 2-tailed paired t-test; n = 3 tissue samples from different patients); GFAP was consistently low in pre- and post-expansion states, in α-SMA -positive ROIs (p = 0.004; 2-tailed paired t-test; n = 3 tissue samples from different patients). And GFAP and α-SMA values in the double negative ROIs were comparably low (Fig. 4L).

Next, we examined vimentin and α-SMA in high-grade glioma tissue. Vimentin is expressed in some tumor cells (70), some activated microglia (71), as well as all endothelial cells (38) and some pericytes (72). Thus, vimentin would be expected to sometimes be near, or even overlapping, with α-SMA (in pericytes) and sometimes to be well-isolated from α-SMA (in other cell types) (68, 69, 73). We observed vimentin and α-SMA signals in the blood vessel wall and surrounding the vessel lumen (Fig. 4M). Vimentin signals were also observed in cells (putative tumor cells or activated microglia) outside of blood vessels (Fig. 4M); with similar observations after 4x expansion (Fig. 4N). However, after post-expansion re-staining (Fig. 4O), new vimentin-positivity appeared in cells, far from blood vessels, that were previously vimentin-negative (Fig. 4M–O). We analyzed vimentin ROIs far away from α-SMA, and found the vimentin staining to go up in these ROIs (Fig. 4P, left; p = 0.0008, 2-tailed paired t-test; n = 3 tissue samples from different patients); in α-SMA ROIs, vimentin also went up significantly (Fig. 4Q, left; p = 0.0001, 2-tailed paired t-test; n = 3 tissue samples from different patients), as expected. In contrast, α-SMA was very little located in the vimentin ROIs Fig. 4P, right; p = 0.0001, 2-tailed paired t-test; n = 3 tissue samples from different patients), and went up in α-SMA ROIs (Fig. 4Q, right; p = 0.04, 2-tailed paired t-test; n = 3 tissue samples from different patients) to some extent – clearly, not all proteins are equally crowded in all cells; perhaps α-SMA is relatively uncrowded to begin with. As before, double negative staining was consistently low (Fig. 4R).

Finally, we examined ionized calcium binding adapter molecule 1 (Iba1) and GFAP in low-grade glioma tissue, again from cortex or white matter. Iba1 is expressed in macrophages and microglia (74). The places we would expect colocalization of these two markers are at sites where an Iba1-positive cell (macrophage, microglia) (74) and a GFAP-positive cell (astrocyte, glioma) touch (75), or where microglia have phagocytosed GFAP-containing fragments (75), or possibly a cell type with a dual astrocytic and macrophage/microglia molecular phenotype (76–79). Accordingly, we chose ROIs that were Iba1-positive or GFAP-positive that were far apart from GFAP and Iba1 staining respectively, as well as double negative ROIs that exhibited neither. We observed GFAP and Iba1 signals in distinct cells before (Fig. 4S) and after expansion (Fig. 4T). However, after post-expansion re-staining (Fig. 4U), new Iba1-positivity appeared in regions that were previously Iba1-negative (Fig. 4S–U) and appeared more continuous (Fig. 4S–U). Iba1 increased in intensity in Iba1-positive ROIs (Fig. 4V, left; p = 0.0009, 2-tailed paired t-test; n = 3 tissue samples from different patients). GFAP also went up in GFAP-positive regions (Fig. 4W, right; p = 0.003 2-tailed paired t-test; n = 3 tissue samples from different patients). In contrast, GFAP was consistently low in pre- and post-expansion states, in Iba1-positive ROIs (Fig. 4V, right; p = 0.0009, 2-tailed paired t-test; n = 3 tissue samples from different patients); Iba1 was consistently low, pre- and post-expansion, in GFAP-positive ROIs (Fig. 4W, left; p = 0.002; 2-tailed paired t-test; n = 3 tissue samples from different patients). And as before, the Iba1 and GFAP values in the double negative ROIs were comparably low (Fig. 4X).

**Fig. 5.**
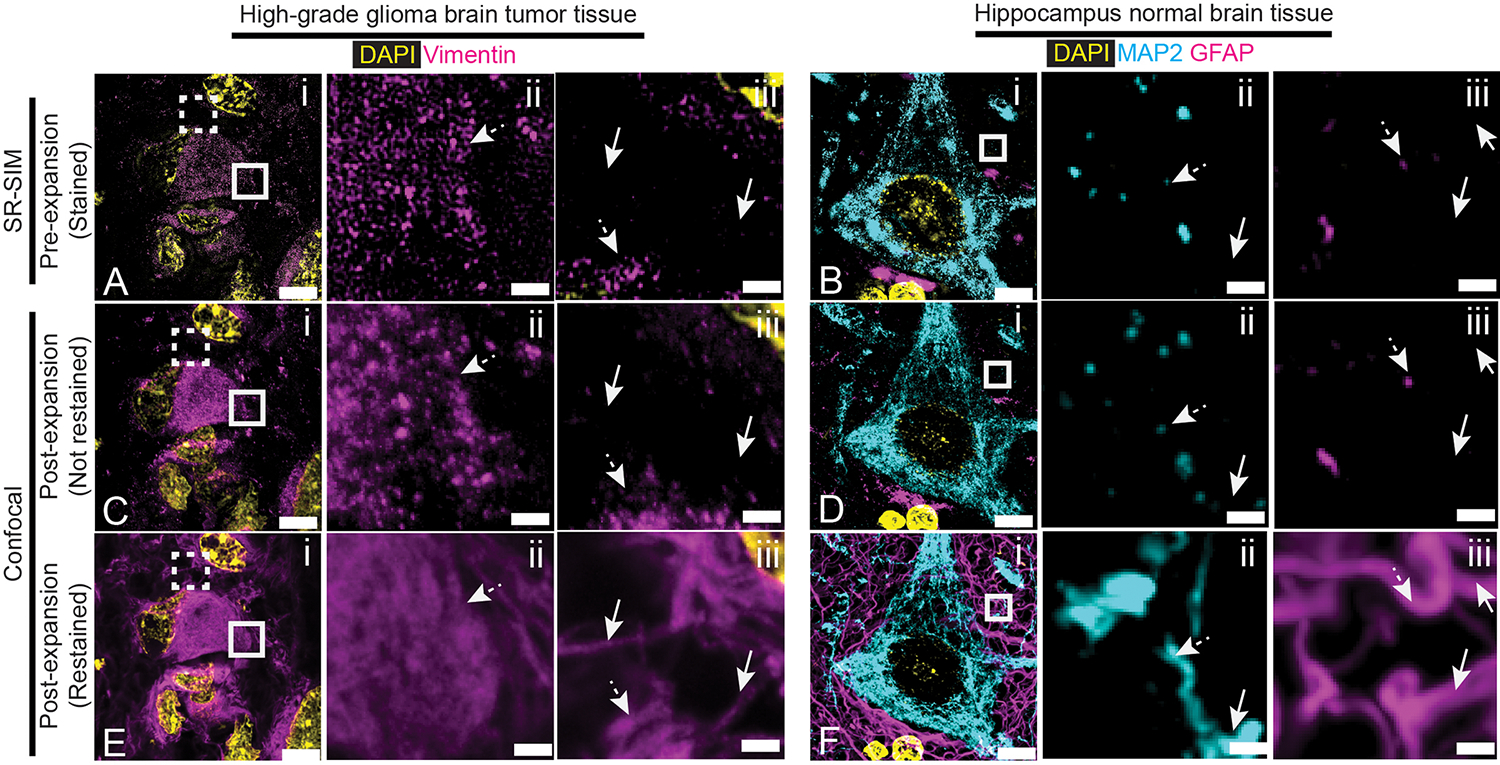
dExPath-mediated protein decrowding reveals cells and structures not detected by SR-SIM imaging of unexpanded tissues. **(A-B)** Representative pre-expansion SR-SIM images of FFPE 5-μm-thick human tissue (processed as in Fig. S3A). (A) High-grade glioma tissue stained for vimentin and DAPI. Solid and dashed white boxes in (i) mark two separate regions shown magnified in (ii) (solid box) and (iii) (dashed box), respectively. Dotted arrows mark regions that appear as punctate and discontinuous in pre-expansion SR-SIM images for vimentin in (ii) and (iii), and solid arrows mark regions that were negative for vimentin in (iii), for comparison to post-expansion staining panels later in this figure. (B) Normal human hippocampus tissue stained for MAP2, GFAP and DAPI. (A) Solid white box in (i) shown magnified in (ii) for MAP2 and in (iii) for GFAP. Arrows as in (A) but for MAP2 and GFAP, in their respective images. **(C-D)** Shown are representative samples used for (A-B) after processing for post-expansion imaging Fig. S3B–D) and not restained. Sum intensity z-projection of an image stack covering the biological thickness of the original slice (used for all expanded images throughout this figure); images were of the same fields of view as in (A-B). Arrows as in (A-B). **(E-F)** Images of the same fields of view as in (A-B) after decrowding and additional restained for vimentin (E), or MAP2 and GFAP (F), followed by DAPI staining and re-expansion to ~4x (Fig. S3E–F), imaged using identical hardware settings as in (C-D). Arrows as in (A-B). Brightness and contrast settings in images (A-F): first set by the ImageJ auto-scaling function, and then manually adjusted to improve contrast for stained structures. Scale bars (in biological units): (A, C, E) left column, 8.3 μm; middle and right columns 840 nm; (B, D, F) left column, 6.0 μm; middle and right columns 500 nm. Linear expansion factors: (C) 4.1x; (D) 4.3x; (E) 4.1x; (F) 4.2x

Having validated the decrowding aspect of dExPath, we next examined whether the improved immunostaining facilitated by dExPath improved images in comparison to those obtained by a similar-resolution super-resolution method that does not decrowd epitopes, SR-SIM. We first performed antigen retrieval and stained high-grade glioma and normal hippocampus with anti-vimentin or anti-MAP/anti-GFAP. Samples were imaged by SR-SIM (Fig. 5A,B), followed by the first part of the dExPath protocol (chemical softening and expansion) (fig. S3A–D) to acquire confocal images post-expansionwith pre-decrowding-staining (Fig. 5C–D). Next, we performed the last part of the dExPath protocol to acquire confocal images post-expansion with post-decrowding-staining (Fig. 5E–F; fig. S3E–F). Both SR-SIM and post-expansion confocal images of pre-decrowding-stained tissue revealed punctate patterns for vimentin (Fig. 5A, 5C), MAP2, and GFAP (Fig. 5B,5D). In contrast, these stains revealed continuous structures after post-decrowding staining (Fig. 5E, 5F), as well as new structures that had not been observed (compare Fig. 5A,C,E vs. 5B,D,F). Thus, dExPath may provide a general solution to the problem of punctate staining appearance in brain tissues, for continuous signals, in super-resolution microscopy (2, 9, 11, 13, 80).

### dExPath retains proteins with improved visualization of targets in normal, glioma, Alzheimer’s disease (AD) and Parkinson’s disease (PD) human brains

We next investigated whether post-decrowding immunostaining improved visualization of protein targets using validated, commercial antibodies useful for pathological analysis. We observed improved visualization compared to standard histopathological chromogenic analysis in immediately adjacent tissue sections of normal human cortex, Alzheimer’s disease (AD) human cortex, and Parkinson’s disease (PD) human cortex (fig. S8). For chromogenic analysis, tissues were imaged pre-expansion, after antigen retrieval, primary antibody staining followed by biotinylated secondary antibody staining and use of 3,3’-diaminobenzidine (DAB). For dExPath, immediately adjacent tissue sections underwent post-decrowding staining with the same primary antibodies, and fluorescent secondaries, under the same conditions (experimental pipeline in fig. S1). dExPath images yielded expected biological targets such as putative neurons (via anti-MAP2 antibody), neurofilaments (via anti-NF-L), AD plaques (via anti-β-amyloid), and PD aggregates (via anti-α-synuclein), but demonstrated better resolution, especially for densely-packed filamentous structures such as MAP2 and NF-L, and more structural detail with amyloid plaques and α-synuclein aggregates, vs. standard DAB analysis.

**Fig. 6.**
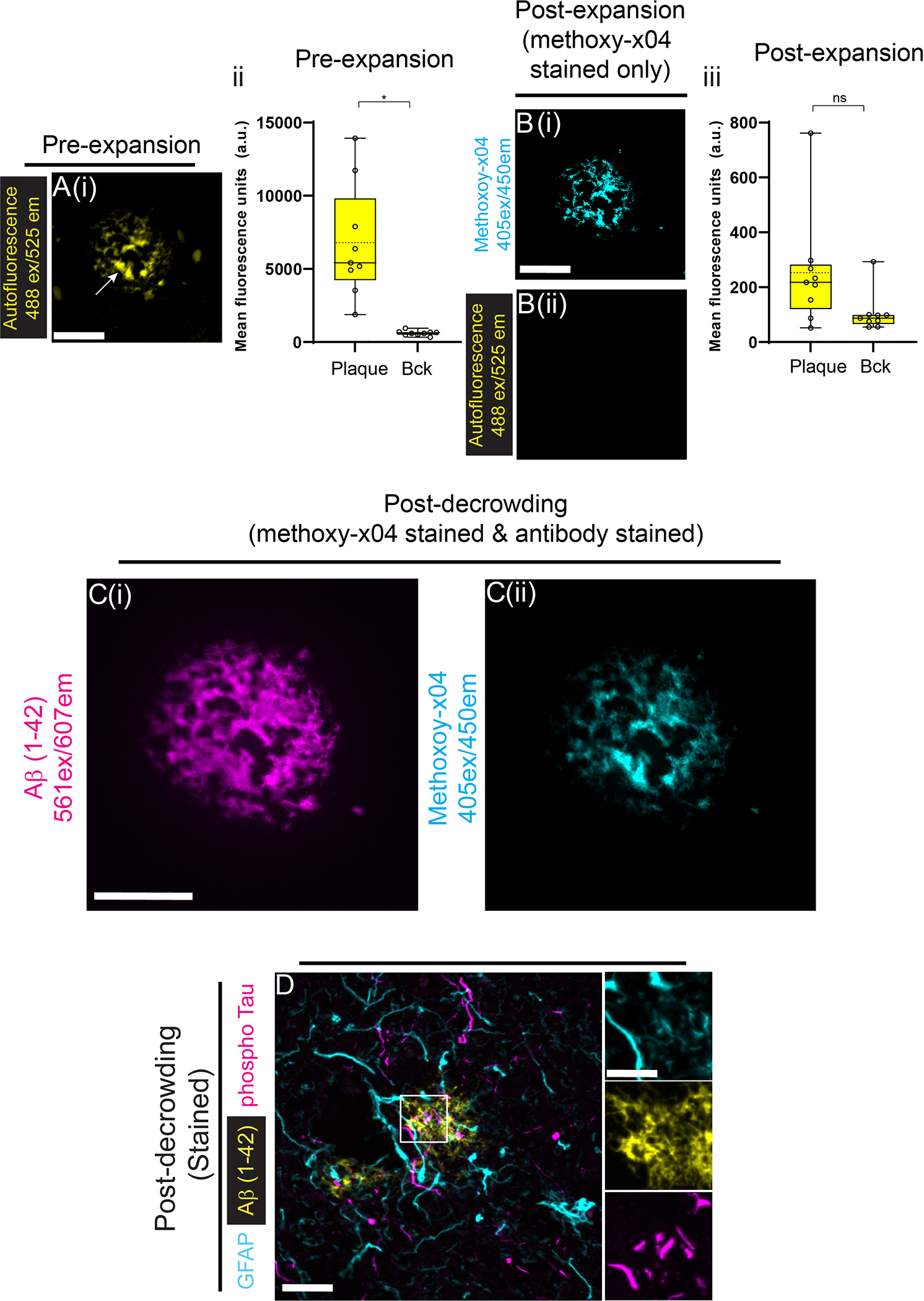
dExPath removes autofluorescence from amyloid plaques and preserves detection of disease markers in Alzheimer’s disease (AD). **(A)** Representative pre-expansion confocal image (single z slice) of an amyloid β plaque within a FFPE 5-μm-thick sample of AD human cortex. The samples underwent processing as in Fig. 1A. (i) Images were acquired for the fluorescent channel setting of 488ex/525em. Solid arrow points to an amyloid β plaque. (ii) Mean fluorescence intensities from pre-expansion images, averaged across regions of interest (ROIs) taken at amyloid β plaque ([plaque], left bar) and background ROIs ([Bck], right bar); Brightness and contrast settings: first set by the ImageJ auto-scaling function, then manually adjusted to improve contrast for amyloid β plaque; quantitative analysis in ii was conducted on raw image data. Box plot: individual values (open circles; 3 plaque measurements were acquired from each patient), median (middle line), mean (dotted line), first and third quartiles (lower and upper box boundaries), lower and upper raw values (whiskers). Statistical testing: 2-tailed paired t-test was applied to amyloid β plaque vs. background, for pre-expansion mean fluorescence intensities. *, p < 0.05; ns, not significant. **(B)** Post-expansion confocal images after the sample from A was processed as in Fig. 1B–D, post-decrowding methoxy-x04 stained, and ~4x linear expansion. Images were acquired for 2 common fluorescent channel settings: (i) a 405ex/450em channel to detect methoxy-x04; and (ii) a 488ex/525em channel to detect plaque autofluorescence as in A. Sum intensity z-projections of image stacks corresponding to the biological thickness of the original slice, taken under identical settings and of the same field of view as in A and displayed under the same settings. (iii) Mean fluorescence intensities, from post-expansion images, averaged across the same amyloid β plaque (left bar) and background (right bar) ROIs used in A. Plots and statistics as in A. **(C)** Images of the same field of view as in (A-B), but the sample was additionally immunostained post-decrowding (as in Fig. 1E), with a (i) Aβ(1–42) (amyloid β protein) monoclonal antibody and images were acquired for the channel settings 561ex/607em channel and (ii) methoxy-x04 using the same spectral ranges as indicated in B at ~2.2x linear expansion; brightness and contrast settings adjusted as in (A) to improve contrast for stained structures. (**D)** Confocal image of a FFPE 5-μm-thick sample of AD human cortex. Sample was processed as in Fig. 1A–D, post-decrowding immunostained, and imaged at ~2.3x linear expansion (Fig. 1E). The tissue sample was stained for Aβ(1–42) (an amyloid β plaque marker) and phospho-tau (a neurofibrillary tangle marker), and GFAP (an astrocyte marker). White boxes mark regions shown magnified in insets on the right. All images are sum intensity z-projections of a confocal image stack. Brightness and contrast settings determined as in C. Scale bars (in biological units): (A - C) 25 μm. Linear expansion factors: (B) 4.1x; (C) 2.2x. Scale bars (in physical units): (D) left panel, 25 μm; inset, 10 μm. Linear expansion factor: (D) 2.3x.

Next, we examined AD brain tissue pre-expansion (Fig. 6) and found that amyloid β plaque autofluorescence (imaged with the fluorescence channel 488ex/525em) was over one order of magnitude greater than background (Fig. 6A; amyloid β plaque vs background: 488ex/525em, p = 0.002; 2-tailed paired t-test; n = 3 tissue samples, each from a different patient). Following dExPath, amyloid β plaque autofluorescence decreased to background (Fig. 6B; amyloid β plaque vs background: 488ex/525em, p = 0.07; 2-tailed paired t-test; n = 3 tissue samples, each from a different patient; images were obtained with the same microscope settings).

Both methoxy-x04 and aβ (1–42) antibody staining overlapped with pre-expansion amyloid β plaque autofluorescence (compare Fig. 6A vs. 6C). We performed additional post-decrowding co-staining for amyloid β, phospho-tau, and GFAP, (Fig. 6D). In this image, plaques with ~20–30 μm diameters were visualized by aβ (1–42) antibody, and they were associated with smaller structures consistent with putative neurofibrillary tangles (81–84) that were positively stained by phospho-tau antibody and surrounded by GFAP-bearing structures, consistent with astrocytes (85).

We surveyed a panel of antibodies commonly used by clinical pathology labs, and found that dExPath could produce high quality images, enabling detection of protein targets across glioma, normal, AD, and PD human tissues (fig. S9). We also observed that dExPath does not lead to protein loss (fig. S10). We demonstrated that dExPath enables multiple rounds of staining and imaging on the same tissue sample, allowing multiplexed imaging with nanoscale resolution of human brain tissues (fig. S11 and fig. S12). dExPath worked well on mouse brain tissue, fixed with standard PFA (fig. S13). dExPath could be applied to tissue sections thicker than used in standard pathology preparations (50–100 μm) (fig. S14; Movie S1 and S2).

### dExPath reveals cell populations exhibiting combinations of disease-state markers in human glioma tissue

Our prior experiments using glioma tissues (Figs. 4 and 5) demonstrated that post-expansion staining increases the intensity, continuity, and number of structures stained for vimentin, Iba1 and GFAP vs. pre-expansion staining. We next asked whether this could lead to detecting more cells carrying specific antigen combinations, which might alter interpretation of clinical biopsies as well as basic understanding of brain tumor biology. For example, cells with both GFAP and vimentin have been reported to be more aggressive than vimentin-negative/GFAP-positive tumor cells (91, 123–125).

**Fig. 7.**
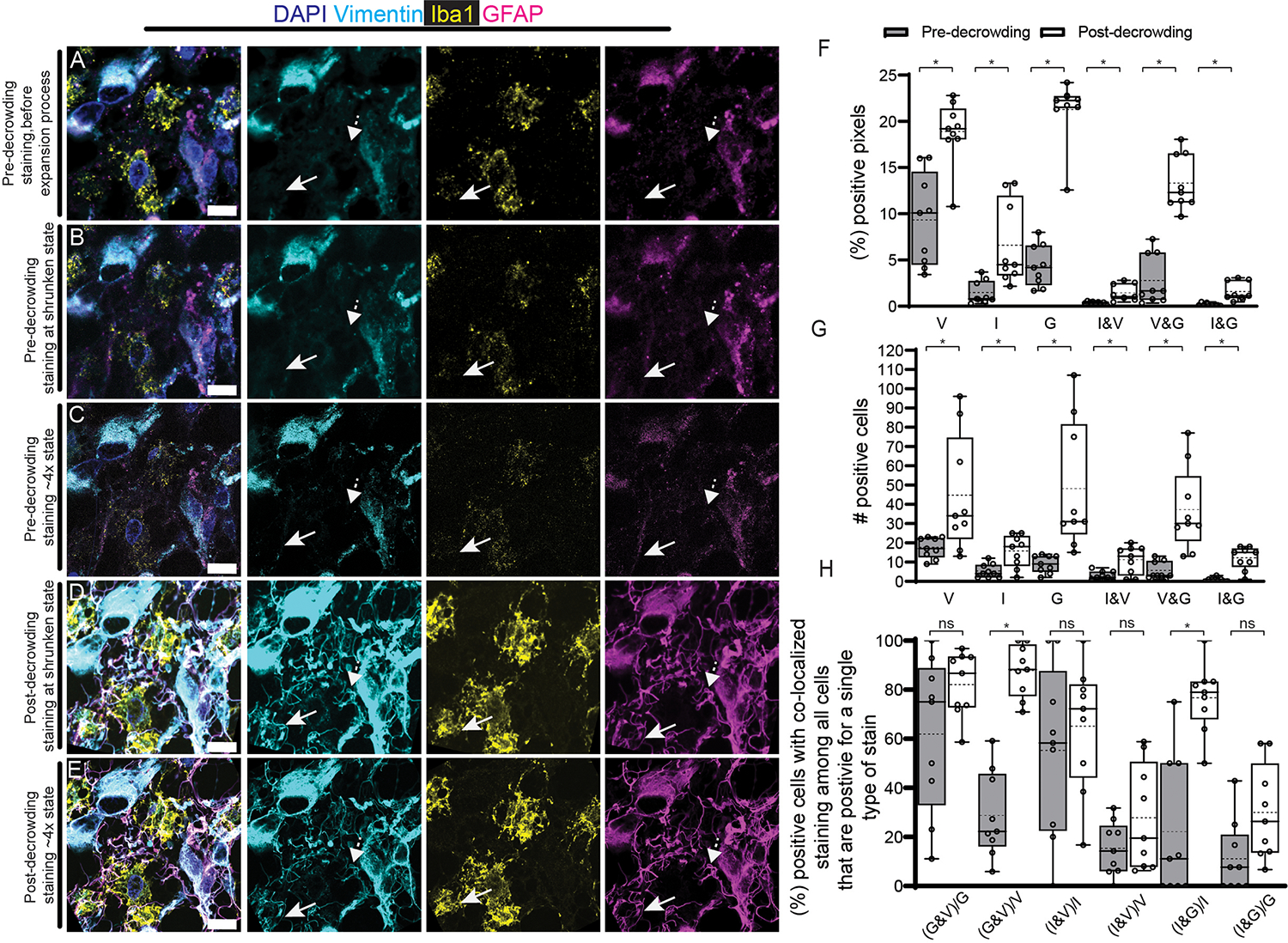
dExPath reveals previously undetected cells defined by single and multiple markers of importance to glioma biology. **(A)** Representative pre-expansion confocal image (single z slice) of a 5-μm-thick FFPE human low-grade glioma specimen. Sample was immunostained for vimentin, Iba1 and GFAP, and DAPI staining (Fig. S3A). Left panel, overlay of all 4 channels; right three panels, individual channels (not including DAPI). Dotted arrows show regions that were vimentin and GFAP negative in pre-expansion images, and solid arrows show regions that were Iba1, GFAP and vimentin negative in pre-expansion images, for comparison to post-decrowding staining panels later in this figure. **(B)** Sample used for (A) after anchoring, gelation), softening (fig. S3B–C), washing with PBS (which results in an expansion factor of ~2.3x), tissue shrinkage (via adding salt) to ~1.3x of the original size, and imaging. Single z slice image centered at the same midpoint of the original slice; images were of the same field of view as in (A), using identical hardware and software settings. Arrows as in (A). **(C)** Sample used for (B) after expansion (fig. S3D) for imaging at ~4x linear expansion. Sum intensity z-projection of an image stack covering the biological thickness of the original slice; images were of the same field of view as in (A), using identical hardware and software settings. Arrows as in (A). **(D)** Post-decrowding stained confocal images of the same field of view as in (A) after decrowding and additional immunostaining for vimentin, Iba1, and GFAP, tissue shrinkage (fig. S3E–F) and imaging at shrunken state. Arrows as in (A). **(E)** Sample used for (D) after expansion (fig. S3D) for imaging at ~4x linear expansion. Arrows as in (A). **(F)** Pixel level analysis of the percent of single or double positive stained pixels, from pre-expansion (gray boxes) and post-decrowding at shrunken state (white boxes) images, for vimentin (V), Iba1 (I), GFAP (G), Iba1 and vimentin (I&V), vimentin and GFAP (V&G), and Iba1 and GFAP (I&G). Values represent the percentage of positive pixels among all pixels in the field of view with 3 different values per sample each corresponding to a different field of view Box plot: individual values (open circles; 3 measurements were acquired from each patient), median (middle line), mean (dotted line), first and third quartiles (lower and upper box boundaries), lower and upper raw values (whiskers), used throughout the graphs of this figure. **(G)** Cell level analysis of single or double positive labeled cells, from pre-expansion and post-decrowding at shrunken state images. Values represent total number of labeled cells in the field of view. **(H)** Cell level analysis of the percentage of double positive labeled cells divided by all single positive cells for a stain in the pre-expansion and post-decrowding at shrunken state images. Values represent the percentage (%) of double positive cells relative to the total number of single positive cells for a stain. Brightness and contrast settings in images (A-E): first set by the ImageJ auto-scaling function, and then manually adjusted (by raising the minimum-intensity threshold and lowering the maximum-intensity threshold) to improve contrast for stained structures but quantitative analysis in (F-H) was conducted on raw image data. Statistical testing: 2-tailed paired t-test (non-Bonferroni corrected) were applied on pre-expansion and post-decrowding values. *, p < 0.05; ns, not significant. Scale bars: (A-E) 11 μm. Linear expansion factors: (B, D) 1.3x; (C, E) 4.4x.

For identification of cells, one may want the enhanced staining afforded by post-expansion staining, without incurring the time cost of imaging an expanded specimen. Thus, we compared the initial pre-decrowded immunostained state to the post-decrowded immunostained state after tissues were shrunk back down to almost their native size (~1.3x) by adding salt. We imaged low-grade glioma tissue sections serially i) after antigen retrieval and pre-decrowding immunostaining (Fig. 7A); ii) after dExPath softening, washing with PBS (which results in an expansion factor of ~2.3x), and tissue shrinkage (via adding salt to attain expansion factor of 1.3x, Fig. 7B); iii) after ~4x expansion (~4x, Fig. 7C); iv) after post-decrowding immunostaining, washing (~2.3x) and shrinkage (~1.3x) (Fig. 7D); and v) after a final expansion step back to ~4x (Fig. 7E). In this way we could decouple the effects of improved resolution from those of improved staining, in the same specimen.

By comparing samples at pre-decrowding vs. post-decrowding staining stages, both in the shrunken ~1.3x state (Fig. 7A,B,D), we observed that post-decrowding immunostaining (Fig. 7D) was able to reveal additional vimentin-, GFAP-, and Iba1-positive staining not detected in the pre-expansion (Fig. 7A) or pre-decrowding staining (Fig. 7B) states, despite the lack of physical magnification in all three cases. Some regions showed increased signal, after post-decrowding immunostaining. For example, some regions showed new structures that were GFAP- and vimentin-positive (compare Fig. 7D–E vs. 7A–C), or Iba1, GFAP and vimentin positive (compare Fig. 7D–E vs. 7A–C). Indeed, when we examined the fraction of pixels that were positive for one or more stains in single z-slices of pre-expansion (Fig. 7A) and post-decrowding (Fig. 7D) images, they increased significantly (Fig. 7F; p <0.05).

These increases in stain-positive pixels translated into increases in the number of cells identified with a label (Fig. 7G; vimentin, p = 0.032, 2-tailed paired t-test, n = 3 tissue samples from different patients; GFAP, p = 0.0071, 2-tailed paired t-test, n = 3 tissue samples from different patients; Iba1, p = 0.0011, 2-tailed paired t-test, n = 3 tissue samples from different patients). Thus, the number of cells corresponding to some tumor cells, some activated microglia, as well as all endothelial cells and some pericytes of mesenchymal origin (vimentin), or astrocytes and glioma cells (GFAP), or macrophages and microglia (Iba1), increased dramatically, suggesting that many cell types important for glioma pathology and response may be quantitatively underestimated by conventional immunostaining.

We next examined the counts of cell types defined by multiple labels. As mentioned earlier, a cell with both GFAP and vimentin is an aggressive tumor cell (91, 123–125), a cell with Iba1 and vimentin is an activated macrophage or microglial cell (71, 74, 127), and a cell with Iba1 and GFAP is either a macrophage or microglial cell that phagocytosed a GFAP expressing cell (astrocyte or tumor cell) or a cell type with a dual astrocytic and macrophage/microglia molecular phenotype (75–77, 79, 122). In each case, the dually labeled cell is qualitatively different from a singly labeled one. Cells positive both for GFAP and vimentin, identified as aggressive/invasive tumor cells, increased in number by about 6-fold with post-expansion vs. pre-expansion staining, suggesting that more aggressive/invasive tumor cells are present than previously thought (Fig. 7G, p = 0.0035, 2-tailed paired t-test, n = 3 tissue samples from different patients). Amongst GFAP-expressing cells, we observed a ~30% increase in the fraction that were vimentin-positive (Fig. 7H, p = 0.036, 2-tailed paired t-test, n = 3 tissue samples from different patients), suggesting that even in low-grade gliomas, a vast majority of tumor cells may be aggressive. Cells double-labeled with Iba1 and vimentin increased by about 4-fold using post-decrowding vs. pre-decrowding staining (Fig. 7G, p = 0.0030, 2-tailed paired t-test, n = 3 tissue samples from different patients), suggesting that a majority of activated macrophages and microglia might currently be overlooked.

Similarly, cells double-labeled with Iba1 and GFAP increased by about 10-fold with post-decrowding vs pre-decrowding staining (Fig. 7G, p = 0.00043, 2-tailed paired t-test, n = 3 tissue samples from different patients). These dual labeled cells are indicative of two cell populations. One population is that of macrophages or microglia, which have phagocytosed GFAP-expressing cells or debris in the tumor tissue sample from astrocytes or tumor cells) (75–77, 79, 122). Macrophage or microglial phagocytosis of GFAP-expressing cells and their debris may support tumor growth via removing of debris such as apoptotic corpses from the tumor microenvironment(75). The second cell population might be a cell population found in diseased states such as stroke and neurodegenerative states (76, 77, 122) and recently found to be present in glioblastoma (79), in which cells share the molecular signatures of both Iba1 expressing cells (macrophages or microglia) and GFAP expressing cells (astrocytes or tumor cells) (75, 79). We show a substantial increase of these Iba1-GFAP dually labeled cells, which can have a protumorigenic role in low-grade gliomas (75, 79). Approximately 80% of Iba1-expressing cells also exhibited GFAP post-expansion, versus only 20% pre-expansion (p = 0.000094, 2-tailed paired t-test, n = 3 tissue samples from different patients, Fig. 7H). In summary, we observed an increase in the percentage of immune cells with phenotypes of importance for the growth of low-grade gliomas.

## Discussion

We describe here a form of expansion microscopy, dExPath, that enables immunostaining of decrowded proteins, for nanoscale visualization of previously unseen biological structures and cell populations in human clinical tissue specimens. By isotropic magnification of human tissues, together with antigen preservation, we achieve protein decrowding, improving the accessibility of target epitopes by antibodies (3–5, 9–12, 128). dExPath works across both normal and diseased brain tissue (low- and high-grade gliomas) types, improving immunostaining for many molecular targets. dExPath enables immunostaining of previously inaccessible cells or subcellular features in normal brain and tumor tissues. Post-expansion staining not only improved the continuity of staining for existing structures, but revealed new, previously invisible, structures of appropriate morphology – as has been noted before in mouse brain tissue (26), but now shown for human brain tissue. It also increased cell counts, including cell types involved with tumor aggression and immune response. These results suggest that post-decrowding staining could, potentially, uncover cell populations that may contribute to early tumor progression but remain undetected with common histological methods. Further studies correlating the presence of these cell populations with clinical outcomes will be necessary to quantify and apply increased clinico-pathological accuracy.

While the clinical applications of these findings need to be explored further, in the context of patient outcomes, treatment regimens, and other relevant factors beyond the scope of this technology paper, to be relevant for any potential future diagnostic or prognostic use, our results show potential for dExPath as a research tool for clinicians and researchers to uncover immunostaining patterns previously unseen in diseased tissues, with further potential, when validated in clinical contexts, for improved diagnostics.

Post-decrowding staining may increase the number of spatially accessible epitopes on a target protein, increasing labeling density of the antibodies and their associated fluorescent signals. Previous studies demonstrate improvement in immunostaining by using small-sized probes (~3nm) (3, 4, 9, 13, 14, 80). dExPath supports the use of conventional off-the-shelf antibodies and can therefore be applied immediately in research settings.

dExPath removed the autofluorescence from lipofuscin found in senescent brain tissues (43–50), improving IHC-mediated detection of intracellular structures. While other methods exist for the masking or quenching of lipofuscin autofluorescence, such as with Sudan Black B (51), they have been associated with limitations including interruption of antibody binding, and reduction of on-target fluorescence (44, 46, 49, 50).

dExPath provides for highly multiplexed immunostaining of decrowded proteins, by retaining protein antigenicity across sequential rounds of antibody stripping and re-staining. These capabilities could be useful for mapping cellular and molecular types and states in normal and diseased tissue microenvironments.

This study examined several antigen targets that have been commonly used as molecular markers to identify specific cell types or states important in normal or diseased brains. For example, dExPath revealed abundant, clearly resolved GFAP-positive filaments in non-diseased human brain tissue, via its decrowding capability. GFAP is involved in physiological and injury-induced functions in which the precise role of this protein remains unknown but its spatial localization appears critical for function (for example, formation of glial scars (129) (130), maintenance of myelinated sites (131), lining of the blood-brain barrier (132), etc.). Visualizing GFAP-positive filaments will facilitate studies of cellular responses to brain injury in clinically relevant human contexts.

Our triple staining experiment (vimentin, Iba1, GFAP) of low-grade glioma tissues showed that dExPath can reveal substantially increased colocalization between these cell type markers, with implications for the analysis of cell populations in glioma biology. For example, our detection of an increased number of previously undetected double-labeled GFAP- and vimentin-positive cells in low-grade glioma tissue may represent a malignant cell subpopulation in these tumors (133–137), usually not detected histologically. Similarly, cells double-labeled with Iba1 and vimentin (interpreted as activated macrophages or microglial cells (71, 74, 127)) may represent a smoldering status of immune activation that could have clinical relevance in these tumors, and cells double-labeled with Iba1 and GFAP may represent an increase in the number of phagocytic macrophages/microglia, or possibly an increase in tumor cells with phagocytic properties with increased invasive ability (74, 78, 79) (138–140).

While we have primarily focused on glioma tissues for this study, dExPath could be applied to other malignancies and/or neuropathologies (AD and PD).

Our study has limitations. Here we optimized dExPath for normal and pathologic brain tissues. However, its application in other tissue types may require additional optimization; for example, a preliminary application of standard dExPath to lymph node tissue did not yield the same low distortion seen for brain (fig. S15), suggesting that additional softening would be useful for tissues more fibrous than the soft brain; lymph nodes studies are beyond the scope of our clinical expertise. dExPath uses low cost, commercially available reagents and instruments (confocal microscopes) found in a conventional basic science laboratory. However, confocal microscopes and some of the reagents, all of which are commercially available, are not standard in clinical laboratories, which might limit the immediate use of dExPath in a standard clinical setting.

dExPath requires multiple manual steps that could benefit from automation to become more efficient for routine use. dExPath may be amenable for automation, given the low amount of tissue deformation present (Fig. 2), the excellent protein preservation evidenced (figs. 4–7; figs, S9, S13), even following multiple rounds of immunostaining and stripping (fig. S11, S12), and the previously published compatibility of gel-embedded tissues for automated multiple rounds of staining and imaging in an RNA context (141). Image analysis would benefit from software that supports accurate quantification and automated registration, currently not part of routine clinical laboratory workflows. Lightsheet microscopy could accelerate imaging of expanded samples (35, 105), but again is not routinely used in the clinic. Studies demonstrating the clinical benefit of dExPath in prognosis or personalized medicine would be required for ultimate clinical adoption. All of these limitations represent urgent opportunities for our field.

In conclusion, dExPath achieves protein decrowding and highly multiplexed immunostaining of archival clinical samples while enabling nanoscale resolution imaging on conventional microscopes, all accomplished using low cost, commercially available reagents and instruments found in conventional basic science or pathology laboratories. We anticipate broad utility of dExPath in many scientific and clinical contexts to advance our understanding of molecular relationships in pathological states and improve diagnostic capabilities.

## Methods

### Study design

The aim of this study was to optimize, and characterize, decrowding expansion pathology (dExPath), for use in normal and pathologic human brain tissues. The goal of this technology is to preserve protein epitopes for post-decrowding staining using commercially available antibodies while expanding tissues isotropically so that nanoscale resolution can be achieved on conventional microscopes. We characterized the ability of dExPath to eliminate tissue autofluorescence associated with lipofuscin and other molecular phenomena (β-amyloid plaques). We characterized the ability of dExPath to support multi-round post-expansion immunostaining. For this we used human tissue microarrays with specific numbers of samples and experimental replicates as indicated in the figures and main text. No blinding, randomization, or prior power calculations were performed.

### Statistical analysis

For sample sizes *n* < 20, individual level data in tabular format can be found in supplemental Table S3. 2-tailed paired student’s *t* test was used when comparing two groups. Statistically significant results were marked with * in figures, with specific *P*-values noted in the text. Statistical analyses were performed using GraphPad prism software.

### Human and animal samples

The normal brain, low- and high-grade glioma human samples used in this study were all 5-μm-thick formalin-fixed paraffin-embedded (FFPE) tissue microarrays obtained from US Biomax or GeneTex. The use of excess deidentified archival specimens does not require informed consent from the subjects.

All procedures involving animals were conducted in accordance with the US National Institutes of Health Guide for the Care and Use of Laboratory Animals and approved by the Massachusetts Institute of Technology Committee on Animal Care. Male and female 12–16 week old, wild type (Swiss Webster) mice were used in this study. Mice were deeply anesthetized with isoflurane and perfused transcardially with ice cold phosphate buffered saline (PBS) followed by ice cold 4% paraformaldehyde (PFA) in phosphate buffered saline (PBS). Brains were harvested and postfixed in the same fixative solution at 4°C overnight. Fixed brains were incubated in 100 mM glycine for 1–2hrs at 4°C and sectioned to 10 or 100 μm-thick slices.

### Tissue processing methods

#### Format conversation, antigen retrieval, pre-expansion NHS ester staining and pre-expansion immunostaining

For FFPE 5-μm thick samples of normal human hippocampus or cortex, human low- or high-grade glioma brain tumor tissues, and human Alzheimer’s disease (AD) or Parkinson’s disease (PD) cortex format conversion (Fig. 1A; fig. S1A) entails deparaffinization and rehydration, which includes 2 washes in 100% xylene for 3 min each, and then serial incubation in the following solutions, for 3 min each at room temperature (RT): (1) 50% xylene + 50% ethanol, (2) 100% ethanol, (3) 95% ethanol (in deionized water, as for all the following ethanol dilution solutions), (4) 80% ethanol, (5) 50% ethanol, (6) deionized water, and (7) 1x PBS. For 4%-PFA 10-μm thick samples of normal mouse brains, format conversion entails 3 washes in 1x PBS at RT for 5 min each.

Following format conversion, tissues samples were designated for 1) pre-expansion immunostaining ( fig. S3A; fig. S4A); 2) no pre-expansion immunostaining and only pre-expansion DAPI staining at 2 μg/ml in 1 x PBS at RT for 15 min (Fig. 1A); 3) or directly to the next steps in our protocol (Fig. 1B–E; fig. S2B–F) without any pre-expansion staining.

For tissue samples that were designated for pre-expansion immunostaining, following format conversion, we applied antigen retrieval to enhance immunostaining(6, 41, 42). Antigen retrieval was performed by incubating tissues in either the softening buffer (20% (weight/volume) sodium dodecyl sulfate (SDS), 100 mM β-mercaptoethanol, 25 mM ethylenediaminetetraacetic acid (EDTA) and 0.5% Triton-X in 50 mM Tris at pH 8 at RT for 1 hr (Fig. 1A) or by microwave heating for 1 min in 5mM citric acid buffer, 0.5% Triton-X, pH 6, because it provided improved collagen staining (fig. S4A). Antigen retrieval was then followed by 3 washes in 1x PBS for 5 min each and blocking at 37°C for 30 min with MAXblock blocking buffer (Active Motif, #15252)(6). Immunostaining was performed by diluting primary antibody in MAXbind Staining buffer (Active Motif, #15253), and incubating tissue samples in the antibody solution at 37°C for 1 hr, at RT for 2.5 hr or at 4°C overnight. The same procedure conditions were applied for secondary antibodies. Primary and secondary antibodies used in this work are listed in Table S2. All pre-expansion stained tissues were immersed in VectaShield mounting media (Vector Laboratories, #H-1000–10) and covered with a No. 1 coverslip prior to imaging

#### Anchoring and gelation

Anchoring and gelation were performed according to previously published protocols(6, 19), and briefly summarized below. Acryloyl-X (a.k.a. 6-((acryloyl)amino)hexanoic acid, succinimidyl ester, here abbreviated AcX, Thermo Fisher Scientific, #A20770) powder was dissolved in anhydrous dimethyl sulfoxide at a concentration of 10 mg/ml and stored in aliquots in a desiccated environment at −20°C. Tissues underwent anchoring by incubation with AcX at a concentration of 0.1 mg/ml in 1x PBS with 0.5% Triton-X, at 4°C for 30 min, followed by 1.5 hrs at 37°C, and then x3 washes with 1x PBS at RT for 5 min each. Next, a monomer solution composed of 1× PBS, 2 M sodium chloride (NaCl), 8.625% (w/v) sodium acrylate, 2.5% (w/v) acrylamide and 0.10% (w/v) N,N′-methylenebisacrylamide (Sigma-Aldrich) was prepared, aliquoted and stored at −20°C. Gelling solution was prepared by mixing the monomer solution with the following chemicals, in the order shown: (1) 4-hydroxy-2,2,6,6-tetramethylpiperidin-1-oxyl (abbreviated as 4-HT; final concentration, 0.01% (w/v)) as an inhibitor of gelation, (2) tetramethylethylenediamine (abbreviated as TEMED; final concentration, 0.2% (w/v)) as an accelerator of gelation, and (3) ammonium persulfate (abbreviated as APS; final concentration, 0.2% (w/v)) as an initiator of gelation. Tissue sections on glass slides were covered with gelling solution, and then a gel chamber was constructed by first placing two No. 1.5 square coverslips (22 mm x 22 mm) as spacers, one at each end of the glass slide and flanking the tissue section in the middle; then, a rectangular coverslip is placed on top of spacers, to enclose the gel chamber, in which the tissue sample is fully immersed in the gelling solution and sandwiched by the glass slide and the top coverslip. Samples were first incubated in a humidified atmosphere at 4°C for 30 min, which slows down gelation rate and enables diffusion of solution into tissues and subsequently incubated in a humidified atmosphere at 37°C for 2.5 hrs to complete gelation (Fig. 1B; fig. S1B, fig. S2B, fig. S3B, fig. S4B).

#### Softening

After gelation, all coverslips were gently removed from the glass slide that carries the gelled tissue. Excessive gel around the tissue sample was trimmed away using a razor blade. Then, tissues were incubated in the softening buffer, which consists of 20% (w/v) SDS, 100 mM β-mercaptoethanol, 25 mM EDTA, 0.5% Triton-X, and Tris 50 mM at pH 8, at 37°C for 30 min followed by 1 hr in an autoclave at 121°C, followed by cooling to RT for 30 min (Fig. 1C; fig. S1C; fig. S2D; fig. S3C; fig. S4D). Tissues were observed to detach from the glass slides during softening, or during subsequent washes with gentle shaking.

#### Expansion with post-decrowding methoxy-xO4 or NHS ester staining

After softening, tissue underwent either decrowding (Fig. 1D; fig. S1D), expansion with post-expansion methoxy-O4 staining, expansion without post-expansion immunostaining (fig. S3D), or decrowding with NHS ester post-expansion staining (fig. S9). After softening, the gelled tissue has detached from the slide and is floating freely in the softening buffer. The tissue is transferred into a clear polystyrene petri dish plate by slowly decanting the buffer solution which contains the gelled tissue into the plate. Then, using a pipette, the excess buffer is removed and discarded. Then 1x PBS is added to the well plate to fully cover the tissue and the petri dish plate is gently shaken at RT to remove excess softening buffer. Then while the gelled tissue was free floating in 1x PBS we used a flat, wide mini paintbrush which we then placed underneath the gelled tissue, ensuring that the paint brush was covering most of the gelled tissue undersurface area, and transferred it into a clear 6-well plate (Clearstar) that contained 1x PBS to completely submerge the tissue. Then the well plate was gently shaken at RT for 3 min. Then the excess 1x PBS was removed using a pipette and new 1x PBS was added to cover the tissue and the well plate gently shaken at RT for 3 min. This process was repeated a total of 5 times, which results in tissues reaching an expansion factor of ~2.3x (Fig. 1D; fig. S1D; fig. S2E; fig. S3E). For decrowding, tissues were washed 5 times with 1x PBS at RT for 3 min each. At this stage, tissues were at a partially expanded state, with ~2.3x linear expansion factor. For expansion with methoxy-x04 (Biotechne, #4920), tissues were stained with methoxy-x04 at a concentration of 0.01 mg/ml in 1x PBS for 2 hours at RT, and then additionally washed in deionized water 3–5 times at RT for 3 min each, expanding the hydrogel-embedded tissue to an expansion factor of ~4x (6, 15, 19). For decrowding with NHS ester post-decrowding staining, we used Alexa Fluor 647 succinimidyl ester at the same concentration and conditions as with pre-expansion staining. For expansion without post-expansion immunostaining, tissues were then additionally washed in deionized water for 3–5 times at RT for 3 min each, to expand the hydrogel-embedded tissue to an expansion factor of ~4x (6, 15, 19) (fig. S3D). A subset of tissue samples was imaged by confocal microscopy at this state, with methods described in the section Image Acquisition, to obtain the post-expansion, pre-decrowding staining images.

#### Post-decrowding immunostaining

Tissues underwent decrowding by washing 5 times with 1x PBS at RT for 3 min which results in an expansion factor of ~2.3x (Fig. 1D; fig. S1D; fig. S2E; fig. S3E). Then, while the gelled tissue was free floating in 1x PBS, we used a flat, wide mini paintbrush placed underneath the gelled tissue, ensuring that the paint brush was covering most of the gelled tissue undersurface area, and transferred it into a new 6-well plate (CellVis) that was subsequently used for imaging containing 1x PBS. We prepared antibody solutions by diluting primary antibody in MAXbind Staining buffer (Active Motif, #15253) and performed post-decrowding immunostaining (Fig. 1E; fig. fig. S1E; fig. S2F; fig. S3F) by incubating tissue samples in the antibody solution at 37°C for 1 hr, at RT for 2.5 hr or at 4°C overnight. Next, excess antibody solution was removed with a pipette and the tissues were washed 3 times with MAXwash buffer (Active Motif, #15254) at RT for 3 min, each time removing excess MAXwash buffer with a pipette. The same procedure conditions were applied for secondary antibodies. For tissues that underwent both pre-expansion and post-decrowding staining, antibody concentrations and incubation conditions were identical to ensure quantitative comparisons pre- and post-expansion. Immunostained tissues were imaged at ~2.3x linear expansion or further expanded by washing with deionized water at RT for 3–5 times for 3 min each to ~4x linear expansion (Fig. 1E; dig. S3F). Then, excess deionized water was removed from the well plate, and the gelled tissue was ready for imaging with a confocal microscope by imaging through the clear glass of the well plate. We performed confocal microscopy at the ~4x state, adhering to the protocol described in the Image Acquisition section, to obtain post-decrowding staining images. Once imaged, tissue can be stored by adding 1x PBS to completely cover tissue, also ensuring the container is covered so tissue does not dry out. Fully ~4x expanded gelled tissues are friable and can easily break if the user tries to lift them up and out of the well plate with a paint brush. To transfer gels between containers do so in the ~2.3x state by washing ~4x expanded gelled tissues in 1x PBS 5 times at RT. A step by step protocol is found in Supplementary Materials (S1).

#### Tissue shrinking

We shrunk tissues to a ~1.3x linear expansion factor by treating with in a high-ionic-strength buffer (1M NaCl + 60 mM MgCl2)(19) following the softening (fig. S3C) and washing with PBS (which results in an expansion factor of ~2.3x) or following post-decrowding staining and washing with PBS (which results in an expansion factor of ~2.3x).Specifically, we washed the tissues 3–5 times with this buffer at RT for 3 min each, until no more tissue shrinkage was observed. We then performed confocal microscopy at this stage, with methods described in the following section Image Acquisition, to obtain pre-decrowding or post-decrowding staining at shrunken state images.

### Imaging processing methods

#### Image acquisition

For confocal imaging, we used a spinning disk confocal system (CSU-W1, Yokogawa) on a Nikon Ti-E microscope. The objective lenses that we used include a 40× 1.15 NA water immersion objective or 10× 0.20 NA air objective. The excitation lasers and emission filters that we used to image each fluorescent dye are the following: 405 nm excitation, 450/50 nm emission filter; 488 nm excitation, 525/40 nm emission filter; 561 nm excitation, 607/36 emission filter; 640 nm excitation, 685/40 emission filter. The following acquisition and display settings apply to all images shown in this study, unless otherwise specified: (1) within the same experiment (as grouped by figures and described in the Results and Figure Legends), all images were obtained with the same laser power, camera settings, and objective lens. (2) For all image display in all figures except Fig. 4, brightness and contrast settings were first individually set by the automated adjustment function in ImageJ, and then manually adjusted (raising the minimum-intensity threshold and lowering the maximum-intensity threshold) to improve contrast for features of interest. For image display of Fig. 4 brightness and contrast settings of images were adjusted so that 1% of the pixels were saturated. None of these changes in the brightness and contrast settings, throughout the entire study, affects the downstream quantitative analysis of fluorescent intensities, which were always applied on raw images, as specified in Results and captions.

For super-resolution structured illumination microscopy (SR-SIM) of samples in the pre-expansion state, for isotropy analyses (Fig. 2A,B) and comparative analyses (Fig. 5A, B), we used a Deltavision OMX Blaze (GE Healthcare) SR-SIM microscope with a 100× 1.40 NA (Olympus) oil objective to acquire the images. Please see Supplemental Materials for details on image processing methods for 1) distortion quantification; 2) image registration between pre-expansion and post-expansion images; 3) image registration between post-expansion images and post-decrowding ~4x expanded Images; 4) Image registration between pre-expansion, pre-decrowding, post-decrowding 1x state and ~4x expanded Images; 5) quantification of lipofuscin autofluorescence removal; 6) fluorescence quantification for protein decrowding;7) quantification of amyloid β plaque autofluorescence removal; and 8) quantification of fluorescence co-localization of vimentin, Iba1, and GFAP in in low-grade gliomas

## Supplementary Material

Table S1

Figure S1

Figure S2

Figure S3

Protocol

Figure S4

Figure S6

Movie S1

Figure S7

Movie S2

Figure S5

Figure S8

Figure S10

Figure S11

Figure S12

Figure S9

Figure S14

Figure S15

Figure S13

## Data Availability

All data are available in the main text or the supplementary materials. The dExPath protocol will also be available online at http://expansionmicroscopy.org. Data will also be available online at https://figshare.com/s/045b3c3676db7f9e553f.

## References

[1] Harris LJ, Skaletsky E, McPherson A, Crystallographic structure of an intact IgG1 monoclonal antibody. J Mol Biol 275, 861–872 (1998). 9480774 10.1006/jmbi.1997.1508

[2] Huang B, Bates M, Zhuang X, Super-resolution fluorescence microscopy. Annu Rev Biochem 78, 993–1016 (2009). 19489737 10.1146/annurev.biochem.77.061906.092014 PMC2835776

[3] Maidorn M, Rizzoli SO, Opazo F, Tools and limitations to study the molecular composition of synapses by fluorescence microscopy. Biochem J 473, 3385–3399 (2016). 27729584 10.1042/BCJ20160366

[4] Kent SP, Ryan KH, Siegel AL, Steric hindrance as a factor in the reaction of labeled antibody with cell surface antigenic determinants. J Histochem Cytochem 26, 618–621 (1978). 357645 10.1177/26.8.357645

[5] Zilly FE , Ca2+ induces clustering of membrane proteins in the plasma membrane via electrostatic interactions. EMBO J 30, 1209–1220 (2011). 21364530 10.1038/emboj.2011.53 PMC3094119

[6] Zhao Y , Nanoscale imaging of clinical specimens using pathology-optimized expansion microscopy. Nat Biotechnol 35, 757–764 (2017). 28714966 10.1038/nbt.3892 PMC5548617

[7] Sahl SJ, Hell SW, Jakobs S, Fluorescence nanoscopy in cell biology. Nat Rev Mol Cell Biol 18, 685–701 (2017). 28875992 10.1038/nrm.2017.71

[8] Ries J, Kaplan C, Platonova E, Eghlidi H, Ewers H, A simple, versatile method for GFP-based super-resolution microscopy via nanobodies. Nat Methods 9, 582–584 (2012). 22543348 10.1038/nmeth.1991

[9] Fornasiero EF, Opazo F, Super-resolution imaging for cell biologists: concepts, applications, current challenges and developments. Bioessays 37, 436–451 (2015). 25581819 10.1002/bies.201400170

[10] Lang T, Rizzoli SO, Membrane protein clusters at nanoscale resolution: more than pretty pictures. Physiology (Bethesda) 25, 116–124 (2010). 20430955 10.1152/physiol.00044.2009

[11] Maidorn M, Olichon A, Rizzoli SO, Opazo F, Nanobodies reveal an extra-synaptic population of SNAP-25 and Syntaxin 1A in hippocampal neurons. MAbs 11, 305–321 (2019). 30466346 10.1080/19420862.2018.1551675 PMC6380399

[12] Hatami A, Albay R 3rd, Monjazeb S, Milton S, Glabe C, Monoclonal antibodies against Abeta42 fibrils distinguish multiple aggregation state polymorphisms in vitro and in Alzheimer disease brain. J Biol Chem 289, 32131–32143 (2014). 25281743 10.1074/jbc.M114.594846 PMC4231689

[13] Opazo F , Aptamers as potential tools for super-resolution microscopy. Nat Methods 9, 938–939 (2012). 23018995 10.1038/nmeth.2179

[14] Mikhaylova M , Resolving bundled microtubules using anti-tubulin nanobodies. Nat Commun 6, 7933 (2015). 26260773 10.1038/ncomms8933 PMC4918323

[15] Chen F, Tillberg PW, Boyden ES, Optical imaging. Expansion microscopy. Science 347, 543–548 (2015). 25592419 10.1126/science.1260088 PMC4312537

[16] Wassie AT, Zhao Y, Boyden ES, Expansion microscopy: principles and uses in biological research. Nat Methods 16, 33–41 (2019). 30573813 10.1038/s41592-018-0219-4 PMC6373868

[17] Gambarotto D , Imaging cellular ultrastructures using expansion microscopy (U-ExM). Nat Methods 16, 71–74 (2019). 30559430 10.1038/s41592-018-0238-1 PMC6314451

[18] Ku T , Multiplexed and scalable super-resolution imaging of three-dimensional protein localization in size-adjustable tissues. Nat Biotechnol 34, 973–981 (2016). 27454740 10.1038/nbt.3641 PMC5070610

[19] Tillberg PW , Protein-retention expansion microscopy of cells and tissues labeled using standard fluorescent proteins and antibodies. Nat Biotechnol 34, 987–992 (2016). 27376584 10.1038/nbt.3625 PMC5068827

[20] Shen FY , Light microscopy based approach for mapping connectivity with molecular specificity. Nat Commun 11, 4632 (2020). 32934230 10.1038/s41467-020-18422-8 PMC7493953

[21] M’Saad O, Bewersdorf J, Light microscopy of proteins in their ultrastructural context. Nat Commun 11, 3850 (2020). 32737322 10.1038/s41467-020-17523-8 PMC7395138

[22] Zwettler FU , Molecular resolution imaging by post-labeling expansion single-molecule localization microscopy (Ex-SMLM). Nat Commun 11, 3388 (2020). 32636396 10.1038/s41467-020-17086-8 PMC7340794

[23] Karagiannis ED , Expansion Microscopy of Lipid Membranes. bioRxiv, 829903 (2019).

[24] Yu CJ , Expansion microscopy of C. elegans. Elife 9, (2020). 10.7554/eLife.46249 PMC7195193 32356725

[25] Sarkar D , Revealing nanostructures in brain tissue via protein decrowding by iterative expansion microscopy. Nat Biomed Eng 6, 1057–1073 (2022). 36038771 10.1038/s41551-022-00912-3 PMC9551354

[26] Sarkar D , Expansion Revealing: Decrowding Proteins to Unmask Invisible Brain Nanostructures. bioRxiv, 2020.2008.2029.273540 (2020).

[27] Schmid M, Prinz TK, Stabler A, Sangerlaub S, Effect of Sodium Sulfite, Sodium Dodecyl Sulfate, and Urea on the Molecular Interactions and Properties of Whey Protein Isolate-Based Films. Front Chem 4, 49 (2016). 28149835 10.3389/fchem.2016.00049 PMC5241285

[28] Xu H, Yang Y, Controlled De-Cross-Linking and Disentanglement of Feather Keratin for Fiber Preparation via a Novel Process. ACS Sustainable Chemistry & Engineering 2, 1404–1410 (2014).

[29] Aumailley M, The laminin family. Cell Adh Migr 7, 48–55 (2013). 23263632 10.4161/cam.22826 PMC3544786

[30] Glockshuber R, Schmidt T, Pluckthun A, The disulfide bonds in antibody variable domains: effects on stability, folding in vitro, and functional expression in Escherichia coli. Biochemistry 31, 1270–1279 (1992). 1736986 10.1021/bi00120a002

[31] Grigorian AL, Bustamante JJ, Hernandez P, Martinez AO, Haro LS, Extraordinarily stable disulfide-linked homodimer of human growth hormone. Protein Sci 14, 902–913 (2005). 15741328 10.1110/ps.041048805 PMC2253441

[32] Park YG , Protection of tissue physicochemical properties using polyfunctional crosslinkers. Nat Biotechnol, (2018). 10.1038/nbt.4281 PMC6579717 30556815

[33] Kleihues P, Soylemezoglu F, Schauble B, Scheithauer BW, Burger PC, Histopathology, classification, and grading of gliomas. Glia 15, 211–221 (1995). 8586458 10.1002/glia.440150303

[34] Chang JB , Iterative expansion microscopy. Nat Methods 14, 593–599 (2017). 28417997 10.1038/nmeth.4261 PMC5560071

[35] Chen F , Nanoscale imaging of RNA with expansion microscopy. Nat Methods 13, 679–684 (2016). 27376770 10.1038/nmeth.3899 PMC4965288

[36] Hirokawa N, Hisanaga S, Shiomura Y, MAP2 is a component of crossbridges between microtubules and neurofilaments in the neuronal cytoskeleton: quick-freeze, deep-etch immunoelectron microscopy and reconstitution studies. J Neurosci 8, 2769–2779 (1988). 3045269 10.1523/JNEUROSCI.08-08-02769.1988 PMC6569399

[37] Yuan A, Rao MV, Veeranna RA Nixon, Neurofilaments and Neurofilament Proteins in Health and Disease. Cold Spring Harb Perspect Biol 9, (2017). 10.1101/cshperspect.a018309 PMC5378049 28373358

[38] Yamada T, Kawamata T, Walker DG, McGeer PL, Vimentin immunoreactivity in normal and pathological human brain tissue. Acta Neuropathol 84, 157–162 (1992). 1523971 10.1007/BF00311389

[39] Wang E, Cairncross JG, Liem RK, Identification of glial filament protein and vimentin in the same intermediate filament system in human glioma cells. Proc Natl Acad Sci U S A 81, 2102–2106 (1984). 6371809 10.1073/pnas.81.7.2102 PMC345445

[40] D’Amico F, Skarmoutsou E, Stivala F, State of the art in antigen retrieval for immunohistochemistry. J Immunol Methods 341, 1–18 (2009). 19063895 10.1016/j.jim.2008.11.007

[41] Brown D , Antigen retrieval in cryostat tissue sections and cultured cells by treatment with sodium dodecyl sulfate (SDS). Histochem Cell Biol 105, 261–267 (1996). 9072183 10.1007/BF01463929

[42] Gustafsson OJ, Arentz G, Hoffmann P, Proteomic developments in the analysis of formalin-fixed tissue. Biochim Biophys Acta 1854, 559–580 (2015). 25315853 10.1016/j.bbapap.2014.10.003

[43] Keller JN , Autophagy, proteasomes, lipofuscin, and oxidative stress in the aging brain. Int J Biochem Cell Biol 36, 2376–2391 (2004). 15325579 10.1016/j.biocel.2004.05.003

[44] Kaluzny J, Purta P, Poskin Z, Rogers JD, Fawzi AA, Ex Vivo Confocal Spectroscopy of Autofluorescence in Age-Related Macular Degeneration. PLoS One 11, e0162869 (2016). 27631087 10.1371/journal.pone.0162869 PMC5024989

[45] Tohma H, Hepworth AR, Shavlakadze T, Grounds MD, Arthur PG, Quantification of ceroid and lipofuscin in skeletal muscle. J Histochem Cytochem 59, 769–779 (2011). 21804079 10.1369/0022155411412185 PMC3261605

[46] Belichenko PV, Fedorov AA, Dahlstrom AB, Quantitative analysis of immunofluorescence and lipofuscin distribution in human cortical areas by dual-channel confocal laser scanning microscopy. J Neurosci Methods 69, 155–161 (1996). 8946318 10.1016/S0165-0270(96)00035-0

[47] Legrand A, Alonso G, Pregnenolone reverses the age-dependent accumulation of glial fibrillary acidic protein within astrocytes of specific regions of the rat brain. Brain Res 802, 125–133 (1998). 9748538 10.1016/s0006-8993(98)00580-0

[48] Bing G, Nguyen XV, Liu M, Markesbery WR, Sun A, Biophysical and biochemical characterization of the intrinsic fluorescence from neurofibrillary tangles. Neurobiol Aging 27, 823–830 (2006). 15946772 10.1016/j.neurobiolaging.2005.04.005

[49] Yin D, Biochemical basis of lipofuscin, ceroid, and age pigment-like fluorophores. Free Radic Biol Med 21, 871–888 (1996). 8902532 10.1016/0891-5849(96)00175-x

[50] Jung T, Bader N, Grune T, Lipofuscin: formation, distribution, and metabolic consequences. Ann N Y Acad Sci 1119, 97–111 (2007). 18056959 10.1196/annals.1404.008

[51] Moreno-Garcia A, Kun A, Calero O, Medina M, Calero M, An Overview of the Role of Lipofuscin in Age-Related Neurodegeneration. Front Neurosci 12, 464 (2018). 30026686 10.3389/fnins.2018.00464 PMC6041410

[52] Brunk UT, Terman A, Lipofuscin: mechanisms of age-related accumulation and influence on cell function. Free Radic Biol Med 33, 611–619 (2002). 12208347 10.1016/s0891-5849(02)00959-0

[53] Singh Kushwaha S, Patro N, Kumar Patro I, A Sequential Study of Age-Related Lipofuscin Accumulation in Hippocampus and Striate Cortex of Rats. Ann Neurosci 25, 223–233 (2018). 31000961 10.1159/000490908 PMC6470335

[54] Kakimoto Y , Myocardial lipofuscin accumulation in ageing and sudden cardiac death. Sci Rep 9, 3304 (2019). 30824797 10.1038/s41598-019-40250-0 PMC6397159

[55] Linstedt AD, Hauri HP, Giantin, a novel conserved Golgi membrane protein containing a cytoplasmic domain of at least 350 kDa. Mol Biol Cell 4, 679–693 (1993). 7691276 10.1091/mbc.4.7.679 PMC300978

[56] Lowe M, Structural organization of the Golgi apparatus. Curr Opin Cell Biol 23, 85–93 (2011). 21071196 10.1016/j.ceb.2010.10.004

[57] Wiedenmann B, Franke WW, Identification and localization of synaptophysin, an integral membrane glycoprotein of Mr 38,000 characteristic of presynaptic vesicles. Cell 41, 1017–1028 (1985). 3924408 10.1016/s0092-8674(85)80082-9

[58] Hol EM, Pekny M, Glial fibrillary acidic protein (GFAP) and the astrocyte intermediate filament system in diseases of the central nervous system. Curr Opin Cell Biol 32, 121–130 (2015). 25726916 10.1016/j.ceb.2015.02.004

[59] Sofroniew MV, Vinters HV, Astrocytes: biology and pathology. Acta Neuropathol 119, 7–35 (2010). 20012068 10.1007/s00401-009-0619-8 PMC2799634

[60] Caceres A, Banker GA, Binder L, Immunocytochemical localization of tubulin and microtubule-associated protein 2 during the development of hippocampal neurons in culture. J Neurosci 6, 714–722 (1986). 3514816 10.1523/JNEUROSCI.06-03-00714.1986 PMC6568475

[61] Bushong EA, Martone ME, Jones YZ, Ellisman MH, Protoplasmic astrocytes in CA1 stratum radiatum occupy separate anatomical domains. J Neurosci 22, 183–192 (2002). 11756501 10.1523/JNEUROSCI.22-01-00183.2002 PMC6757596

[62] Colombo JA, Gayol S, Yanez A, Marco P, Immunocytochemical and electron microscope observations on astroglial interlaminar processes in the primate neocortex. J Neurosci Res 48, 352–357 (1997). 9169861

[63] Brehar FM, Arsene D, Brinduse LA, Gorgan MR, Immunohistochemical analysis of GFAP-delta and nestin in cerebral astrocytomas. Brain Tumor Pathol 32, 90–98 (2015). 25178519 10.1007/s10014-014-0199-8

[64] Brenner M, Role of GFAP in CNS injuries. Neurosci Lett 565, 7–13 (2014). 24508671 10.1016/j.neulet.2014.01.055 PMC4049287

[65] Choi KC, Kwak SE, Kim JE, Sheen SH, Kang TC, Enhanced glial fibrillary acidic protein-delta expression in human astrocytic tumor. Neurosci Lett 463, 182–187 (2009). 19647039 10.1016/j.neulet.2009.07.076

[66] Alarcon-Martinez L , Capillary pericytes express alpha-smooth muscle actin, which requires prevention of filamentous-actin depolymerization for detection. Elife 7, (2018). 10.7554/eLife.34861 PMC5862523 29561727

[67] Verbeek MM, Otte-Holler I, Wesseling P, Ruiter DJ, de Waal RM, Induction of alpha-smooth muscle actin expression in cultured human brain pericytes by transforming growth factor-beta 1. Am J Pathol 144, 372–382 (1994). 8311120 PMC1887139

[68] Yamazaki T, Mukouyama YS, Tissue Specific Origin, Development, and Pathological Perspectives of Pericytes. Front Cardiovasc Med 5, 78 (2018). 29998128 10.3389/fcvm.2018.00078 PMC6030356

[69] Bergers G, Song S, The role of pericytes in blood-vessel formation and maintenance. Neuro Oncol 7, 452–464 (2005). 16212810 10.1215/S1152851705000232 PMC1871727

[70] Herpers MJ, Ramaekers FC, Aldeweireldt J, Moesker O, Slooff J, Co-expression of glial fibrillary acidic protein- and vimentin-type intermediate filaments in human astrocytomas. Acta Neuropathol 70, 333–339 (1986). 3020864 10.1007/BF00686093

[71] Graeber MB, Streit WJ, Kreutzberg GW, The microglial cytoskeleton: vimentin is localized within activated cells in situ. J Neurocytol 17, 573–580 (1988). 3193132 10.1007/BF01189811

[72] Diaz-Flores L, Gutierrez R, Varela H, Rancel N, Valladares F, Microvascular pericytes: a review of their morphological and functional characteristics. Histol Histopathol 6, 269–286 (1991). 1802127

[73] Cheng L , Glioblastoma stem cells generate vascular pericytes to support vessel function and tumor growth. Cell 153, 139–152 (2013). 23540695 10.1016/j.cell.2013.02.021 PMC3638263

[74] Deininger MH, Seid K, Engel S, Meyermann R, Schluesener HJ, Allograft inflammatory factor-1 defines a distinct subset of infiltrating macrophages/microglial cells in rat and human gliomas. Acta Neuropathol 100, 673–680 (2000). 11078219 10.1007/s004010000233

[75] Saavedra-Lopez E , Phagocytic glioblastoma-associated microglia and macrophages populate invading pseudopalisades. Brain Commun 2, fcz043 (2020). 32954312 10.1093/braincomms/fcz043 PMC7491442

[76] Diaz-Amarilla P , Phenotypically aberrant astrocytes that promote motoneuron damage in a model of inherited amyotrophic lateral sclerosis. Proc Natl Acad Sci U S A 108, 18126–18131 (2011). 22010221 10.1073/pnas.1110689108 PMC3207668

[77] Morizawa YM , Reactive astrocytes function as phagocytes after brain ischemia via ABCA1-mediated pathway. Nat Commun 8, 28 (2017). 28642575 10.1038/s41467-017-00037-1 PMC5481424

[78] Huysentruyt LC, Akgoc Z, Seyfried TN, Hypothesis: are neoplastic macrophages/microglia present in glioblastoma multiforme? ASN Neuro 3, (2011). 10.1042/AN20110011 PMC3178415 21834792

[79] Persson A, Englund E, Phagocytic properties in tumor astrocytes. Neuropathology 32, 252–260 (2012). 22098621 10.1111/j.1440-1789.2011.01266.x

[80] Gomes de Castro MA, Hobartner C, Opazo F, Aptamers provide superior stainings of cellular receptors studied under super-resolution microscopy. PLoS One 12, e0173050 (2017). 28235049 10.1371/journal.pone.0173050 PMC5325610

[81] DeTure MA, Dickson DW, The neuropathological diagnosis of Alzheimer’s disease. Mol Neurodegener 14, 32 (2019). 31375134 10.1186/s13024-019-0333-5 PMC6679484

[82] Serrano-Pozo A, Frosch MP, Masliah E, Hyman BT, Neuropathological alterations in Alzheimer disease. Cold Spring Harb Perspect Med 1, a006189 (2011). 22229116 10.1101/cshperspect.a006189 PMC3234452

[83] Cras P , Senile plaque neurites in Alzheimer disease accumulate amyloid precursor protein. Proc Natl Acad Sci U S A 88, 7552–7556 (1991). 1652752 10.1073/pnas.88.17.7552 PMC52339

[84] Perl DP, Neuropathology of Alzheimer’s disease. Mt Sinai J Med 77, 32–42 (2010). 20101720 10.1002/msj.20157 PMC2918894

[85] Querol-Vilaseca M , Nanoscale structure of amyloid-beta plaques in Alzheimer’s disease. Sci Rep 9, 5181 (2019). 30914681 10.1038/s41598-019-41443-3 PMC6435662

[86] Olar A , IDH mutation status and role of WHO grade and mitotic index in overall survival in grade II-III diffuse gliomas. Acta Neuropathol 129, 585–596 (2015). 25701198 10.1007/s00401-015-1398-z PMC4369189

[87] Pusztaszeri MP, Seelentag W, Bosman FT, Immunohistochemical expression of endothelial markers CD31, CD34, von Willebrand factor, and Fli-1 in normal human tissues. J Histochem Cytochem 54, 385–395 (2006). 16234507 10.1369/jhc.4A6514.2005

[88] De Meyer SF, Stoll G, Wagner DD, Kleinschnitz C, von Willebrand factor: an emerging target in stroke therapy. Stroke 43, 599–606 (2012). 22180250 10.1161/STROKEAHA.111.628867 PMC4102321

[89] Yang AC , A human brain vascular atlas reveals diverse mediators of Alzheimer’s risk. Nature 603, 885–892 (2022). 35165441 10.1038/s41586-021-04369-3 PMC9635042

[90] Yung WK, Luna M, Borit A, Vimentin and glial fibrillary acidic protein in human brain tumors. J Neurooncol 3, 35–38 (1985). 3889231 10.1007/BF00165169

[91] Mendez MG, Kojima S, Goldman RD, Vimentin induces changes in cell shape, motility, and adhesion during the epithelial to mesenchymal transition. FASEB J 24, 1838–1851 (2010). 20097873 10.1096/fj.09-151639 PMC2874471

[92] Chacinska A, Koehler CM, Milenkovic D, Lithgow T, Pfanner N, Importing mitochondrial proteins: machineries and mechanisms. Cell 138, 628–644 (2009). 19703392 10.1016/j.cell.2009.08.005 PMC4099469

[93] C. A. Wurm , Nanoscale distribution of mitochondrial import receptor Tom20 is adjusted to cellular conditions and exhibits an inner-cellular gradient. Proc Natl Acad Sci U S A 108, 13546–13551 (2011). 21799113 10.1073/pnas.1107553108 PMC3158204

[94] Wang X, Gerdes HH, Transfer of mitochondria via tunneling nanotubes rescues apoptotic PC12 cells. Cell Death Differ 22, 1181–1191 (2015). 25571977 10.1038/cdd.2014.211 PMC4572865

[95] Pasquier J , Preferential transfer of mitochondria from endothelial to cancer cells through tunneling nanotubes modulates chemoresistance. J Transl Med 11, 94 (2013). 23574623 10.1186/1479-5876-11-94 PMC3668949

[96] Roehlecke C, Schmidt MHH, Tunneling Nanotubes and Tumor Microtubes in Cancer. Cancers (Basel) 12, (2020). 10.3390/cancers12040857 PMC7226329 32244839

[97] Wang L, Wang X, Wang CC, Protein disulfide-isomerase, a folding catalyst and a redox-regulated chaperone. Free Radic Biol Med 83, 305–313 (2015). 25697778 10.1016/j.freeradbiomed.2015.02.007

[98] Xu S, Sankar S, Neamati N, Protein disulfide isomerase: a promising target for cancer therapy. Drug Discov Today 19, 222–240 (2014). 24184531 10.1016/j.drudis.2013.10.017

[99] Ligon KL , The oligodendroglial lineage marker OLIG2 is universally expressed in diffuse gliomas. J Neuropathol Exp Neurol 63, 499–509 (2004). 15198128 10.1093/jnen/63.5.499

[100] Ishizawa K, Komori T, Shimada S, Hirose T, Olig2 and CD99 are useful negative markers for the diagnosis of brain tumors. Clin Neuropathol 27, 118–128 (2008). 18552083 10.5414/npp27118

[101] Camelo-Piragua S , Mutant IDH1-specific immunohistochemistry distinguishes diffuse astrocytoma from astrocytosis. Acta Neuropathol 119, 509–511 (2010). 20044756 10.1007/s00401-009-0632-y PMC2864729

[102] Louis DN , The 2016 World Health Organization Classification of Tumors of the Central Nervous System: a summary. Acta Neuropathol 131, 803–820 (2016). 27157931 10.1007/s00401-016-1545-1

[103] Reuss DE , ATRX and IDH1-R132H immunohistochemistry with subsequent copy number analysis and IDH sequencing as a basis for an “integrated” diagnostic approach for adult astrocytoma, oligodendroglioma and glioblastoma. Acta Neuropathol 129, 133–146 (2015). 25427834 10.1007/s00401-014-1370-3

[104] Koschmann C , ATRX loss promotes tumor growth and impairs nonhomologous end joining DNA repair in glioma. Sci Transl Med 8, 328ra328 (2016). 10.1126/scitranslmed.aac8228 PMC5381643 26936505

[105] Gao R , Cortical column and whole-brain imaging with molecular contrast and nanoscale resolution. Science 363, (2019). 10.1126/science.aau8302 PMC6481610 30655415

[106] Boggs JM, Myelin basic protein: a multifunctional protein. Cell Mol Life Sci 63, 1945–1961 (2006). 16794783 10.1007/s00018-006-6094-7 PMC11136439

[107] Roberts RC, Roche JK, McCullumsmith RE, Localization of excitatory amino acid transporters EAAT1 and EAAT2 in human postmortem cortex: a light and electron microscopic study. Neuroscience 277, 522–540 (2014). 25064059 10.1016/j.neuroscience.2014.07.019 PMC4164610

[108] Katsetos CD, Herman MM, Mork SJ, Class III beta-tubulin in human development and cancer. Cell Motil Cytoskeleton 55, 77–96 (2003). 12740870 10.1002/cm.10116

[109] Draberova E, Lukas Z, Ivanyi D, Viklicky V, Draber P, Expression of class III beta-tubulin in normal and neoplastic human tissues. Histochem Cell Biol 109, 231–239 (1998). 9541471 10.1007/s004180050222

[110] Trojanowski JQ, Lee VM, Schlaepfer WW, An immunohistochemical study of human central and peripheral nervous system tumors, using monoclonal antibodies against neurofilaments and glial filaments. Hum Pathol 15, 248–257 (1984). 6538179 10.1016/s0046-8177(84)80188-4

[111] Wierzba-Bobrowicz T, Schmidt-Sidor B, Gwiazda E, Bertrand E, The significance of immunocytochemical markers, synaptophysin and neurofilaments in diagnosis of ganglioglioma. Folia Neuropathol 37, 157–161 (1999). 10581850

[112] Davydova D , Bassoon specifically controls presynaptic P/Q-type Ca(2+) channels via RIM-binding protein. Neuron 82, 181–194 (2014). 24698275 10.1016/j.neuron.2014.02.012

[113] Kim E, Sheng M, PDZ domain proteins of synapses. Nat Rev Neurosci 5, 771–781 (2004). 15378037 10.1038/nrn1517

[114] Choong CJ, Mochizuki H, Neuropathology of alpha-synuclein in Parkinson’s disease. Neuropathology 42, 93–103 (2022). 35362115 10.1111/neup.12812

[115] Wakabayashi K, Hayashi S, Yoshimoto M, Kudo H, Takahashi H, NACP/alpha-synuclein-positive filamentous inclusions in astrocytes and oligodendrocytes of Parkinson’s disease brains. Acta Neuropathol 99, 14–20 (2000). 10651022 10.1007/pl00007400

[116] Dickson DW, Neuropathology of Parkinson disease. Parkinsonism Relat Disord 46 Suppl 1, S30–S33 (2018). 28780180 10.1016/j.parkreldis.2017.07.033 PMC5718208

[117] Goedert M, Jakes R, Spillantini MG, The Synucleinopathies: Twenty Years On. J Parkinsons Dis 7, S51–S69 (2017). 28282814 10.3233/JPD-179005 PMC5345650

[118] Tofaris GK , Pathological changes in dopaminergic nerve cells of the substantia nigra and olfactory bulb in mice transgenic for truncated human alpha-synuclein(1–120): implications for Lewy body disorders. J Neurosci 26, 3942–3950 (2006). 16611810 10.1523/JNEUROSCI.4965-05.2006 PMC6673887

[119] Booth HDE, Hirst WD, Wade-Martins R, The Role of Astrocyte Dysfunction in Parkinson’s Disease Pathogenesis. Trends Neurosci 40, 358–370 (2017). 28527591 10.1016/j.tins.2017.04.001 PMC5462417

[120] Sonustun B , Pathological Relevance of Post-Translationally Modified Alpha-Synuclein (pSer87, pSer129, nTyr39) in Idiopathic Parkinson’s Disease and Multiple System Atrophy. Cells 11, (2022). 10.3390/cells11050906 PMC8909017 35269528

[121] Hohenester E, Yurchenco PD, Laminins in basement membrane assembly. Cell Adh Migr 7, 56–63 (2013). 23076216 10.4161/cam.21831 PMC3544787

[122] van Bodegraven EJ, van Asperen JV, Robe PAJ, Hol EM, Importance of GFAP isoform-specific analyses in astrocytoma. Glia 67, 1417–1433 (2019). 30667110 10.1002/glia.23594 PMC6617972

[123] Lin L , Analysis of expression and prognostic significance of vimentin and the response to temozolomide in glioma patients. Tumour Biol 37, 15333–15339 (2016). 27704357 10.1007/s13277-016-5462-7

[124] Nowicki MO, Hayes JL, Chiocca EA, Lawler SE, Proteomic Analysis Implicates Vimentin in Glioblastoma Cell Migration. Cancers (Basel) 11, (2019). 10.3390/cancers11040466 PMC6521049 30987208

[125] Jan HJ , Osteopontin regulates human glioma cell invasiveness and tumor growth in mice. Neuro Oncol 12, 58–70 (2010). 20150368 10.1093/neuonc/nop013 PMC2940564

[126] Zhao J , High Expression of Vimentin is Associated With Progression and a Poor Outcome in Glioblastoma. Appl Immunohistochem Mol Morphol 26, 337–344 (2018). 27556820 10.1097/PAI.0000000000000420

[127] Jiang SX, Slinn J, Aylsworth A, Hou ST, Vimentin participates in microglia activation and neurotoxicity in cerebral ischemia. J Neurochem 122, 764–774 (2012). 22681613 10.1111/j.1471-4159.2012.07823.x

[128] Lau L, Lee YL, Sahl SJ, Stearns T, Moerner WE, STED microscopy with optimized labeling density reveals 9-fold arrangement of a centriole protein. Biophys J 102, 2926–2935 (2012). 22735543 10.1016/j.bpj.2012.05.015 PMC3379620

[129] Liedtke W, Edelmann W, Chiu FC, Kucherlapati R, Raine CS, Experimental autoimmune encephalomyelitis in mice lacking glial fibrillary acidic protein is characterized by a more severe clinical course and an infiltrative central nervous system lesion. Am J Pathol 152, 251–259 (1998). 9422542 PMC1858102

[130] Xu K, Malouf AT, Messing A, Silver J, Glial fibrillary acidic protein is necessary for mature astrocytes to react to beta-amyloid. Glia 25, 390–403 (1999). 10028921 10.1002/(sici)1098-1136(19990215)25:4<390::aid-glia8>3.0.co;2-7

[131] Liedtke W , GFAP is necessary for the integrity of CNS white matter architecture and long-term maintenance of myelination. Neuron 17, 607–615 (1996). 8893019 10.1016/s0896-6273(00)80194-4

[132] Pekny M, Stanness KA, Eliasson C, Betsholtz C, Janigro D, Impaired induction of blood-brain barrier properties in aortic endothelial cells by astrocytes from GFAP-deficient mice. Glia 22, 390–400 (1998). 9517571

[133] De Pascalis C , Intermediate filaments control collective migration by restricting traction forces and sustaining cell-cell contacts. J Cell Biol 217, 3031–3044 (2018). 29980627 10.1083/jcb.201801162 PMC6122997

[134] Reifenberger G, Bilzer T, Seitz RJ, Wechsler W, Expression of vimentin and glial fibrillary acidic protein in ethylnitrosourea-induced rat gliomas and glioma cell lines. Acta Neuropathol 78, 270–282 (1989). 2475009 10.1007/BF00687757

[135] Battaglia RA, Delic S, Herrmann H, Snider NT, Vimentin on the move: new developments in cell migration. F1000Res 7, (2018). 10.12688/f1000research.15967.1 PMC6241562 30505430

[136] Osswald M , Brain tumour cells interconnect to a functional and resistant network. Nature 528, 93–98 (2015). 26536111 10.1038/nature16071

[137] Weil S , Tumor microtubes convey resistance to surgical lesions and chemotherapy in gliomas. Neuro Oncol 19, 1316–1326 (2017). 28419303 10.1093/neuonc/nox070 PMC5596180

[138] Lewis CE, Pollard JW, Distinct role of macrophages in different tumor microenvironments. Cancer Res 66, 605–612 (2006). 16423985 10.1158/0008-5472.CAN-05-4005

[139] Qian BZ, Pollard JW, Macrophage diversity enhances tumor progression and metastasis. Cell 141, 39–51 (2010). 20371344 10.1016/j.cell.2010.03.014 PMC4994190

[140] Kubelt C, Hattermann K, Sebens S, Mehdorn HM, Held-Feindt J, Epithelial-to-mesenchymal transition in paired human primary and recurrent glioblastomas. Int J Oncol 46, 2515–2525 (2015). 25845427 10.3892/ijo.2015.2944

[141] Alon S , Expansion sequencing: Spatially precise in situ transcriptomics in intact biological systems. Science 371, (2021). 10.1126/science.aax2656 PMC7900882 33509999

[142] Florian D, Kock H, Plankensteiner K, Glavanovics M, Auto focus and image registration techniques for infrared imaging of microelectronic devices. Meas Sci Technol 24, (2013).

[143] Stringer C, Wang T, Michaelos M, Pachitariu M, Cellpose: a generalist algorithm for cellular segmentation. Nat Methods 18, 100–106 (2021). 33318659 10.1038/s41592-020-01018-x

[144] Otsu N, A Threshold Selection Method from Gray-Level Histograms. IEEE Transactions on Systems, Man, and Cybernetics 9, 62–66 (1979).

