# Supplementary figures and images for: Improved immunostaining of nanostructures and cells in human brain specimens through expansion-mediated protein decrowding
Source: Sci Transl Med. Author manuscript; Available in PMC 2024 Mar 4. (PMC10911838; doi:10.1126/scitranslmed.abo0049)

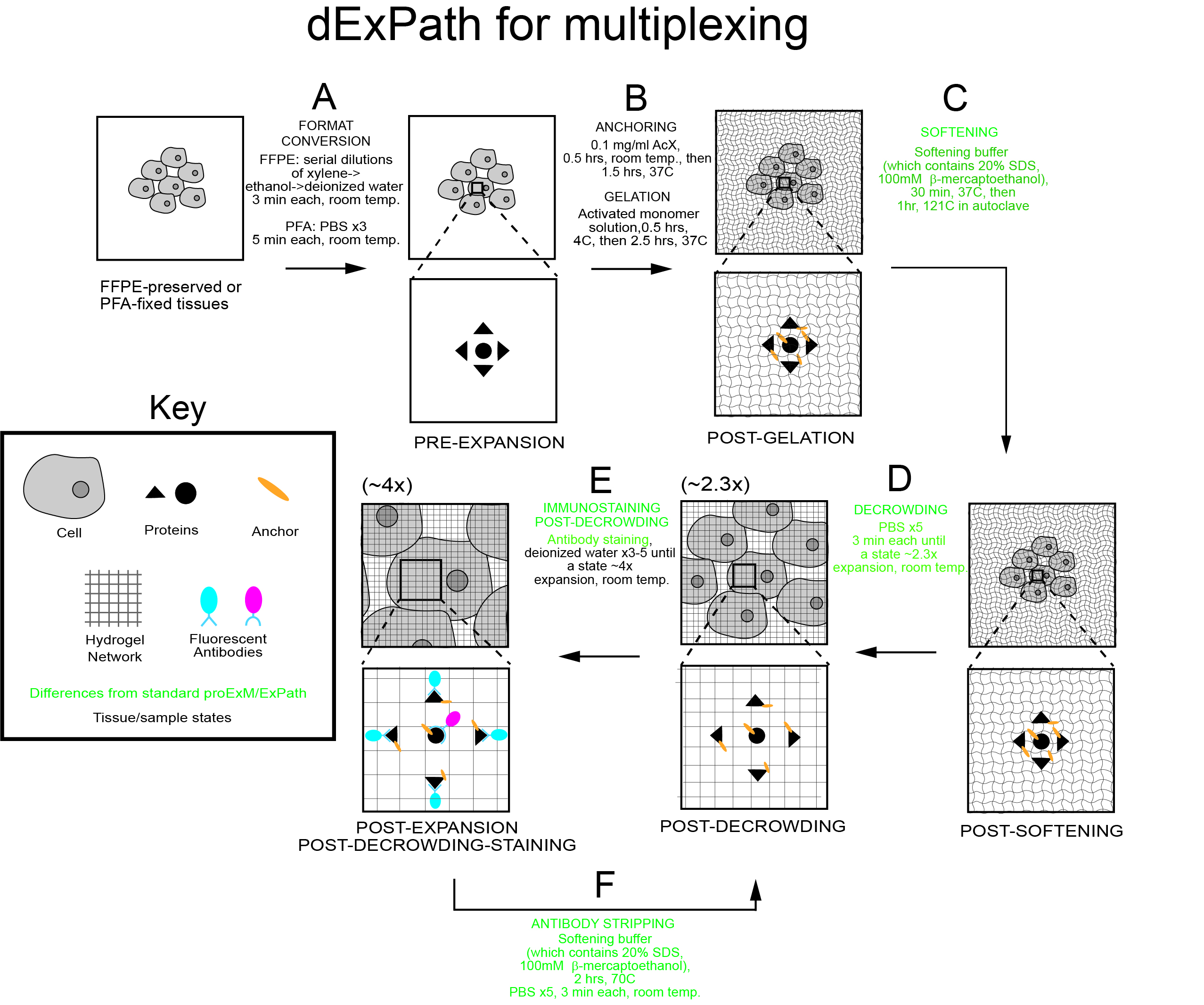

Supplement: Figure S1 [file NIHMS1967379-supplement-Figure_S1.jpg]

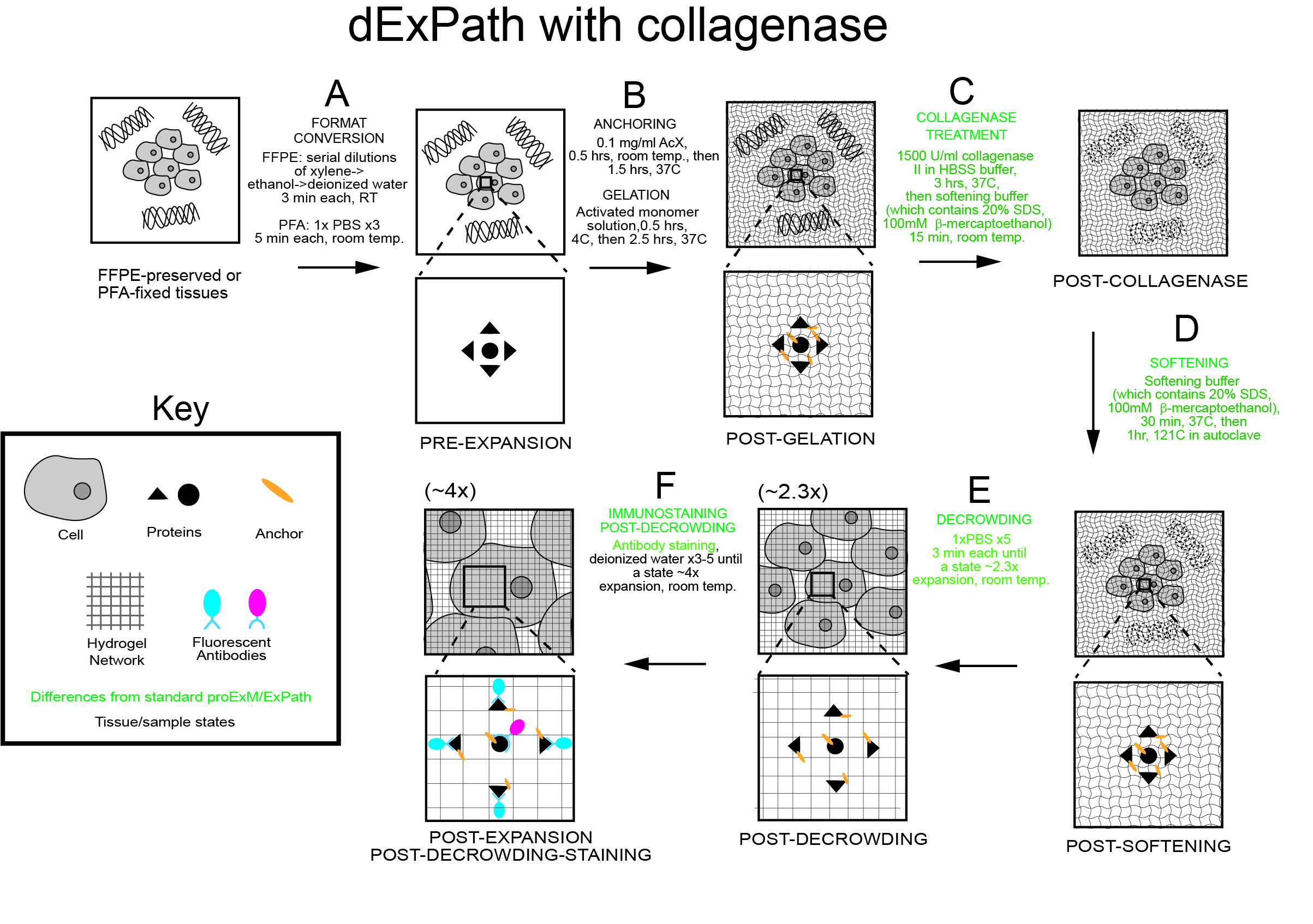

Supplement: Figure S2 [file NIHMS1967379-supplement-Figure_S2.jpg]

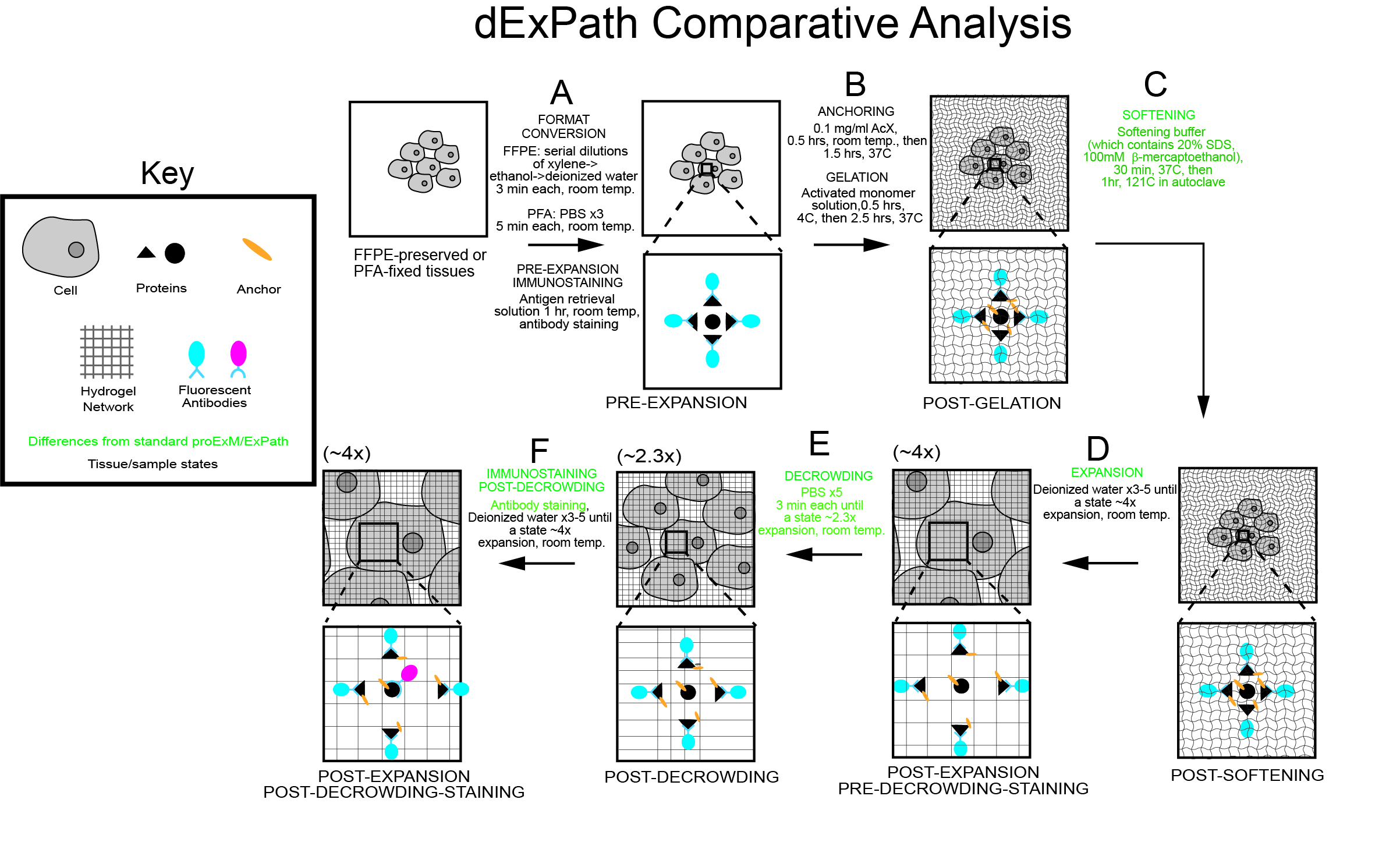

Supplement: Figure S3 [file NIHMS1967379-supplement-Figure_S3.jpg]

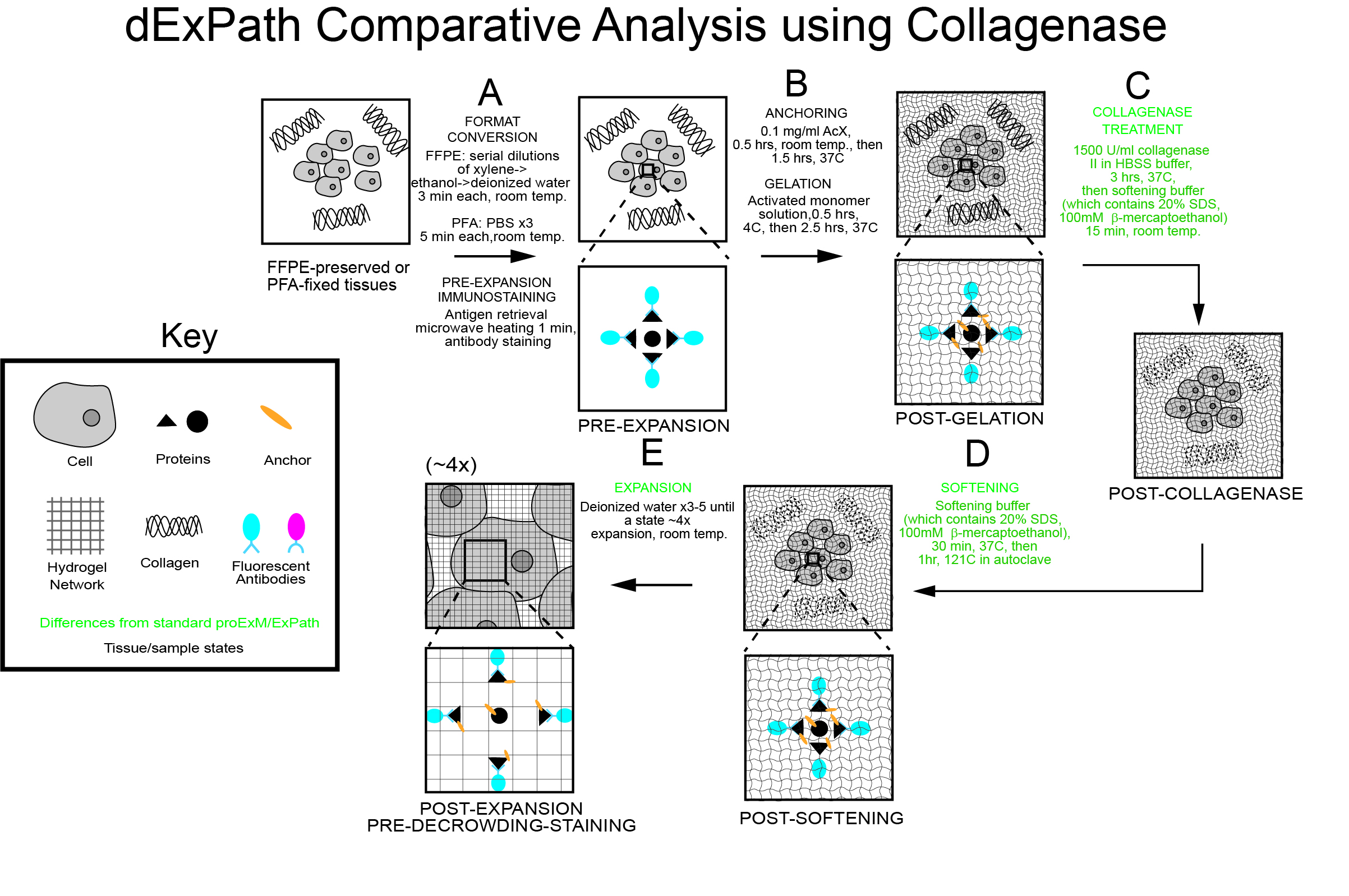

Supplement: Figure S4 [file NIHMS1967379-supplement-Figure_S4.jpg]

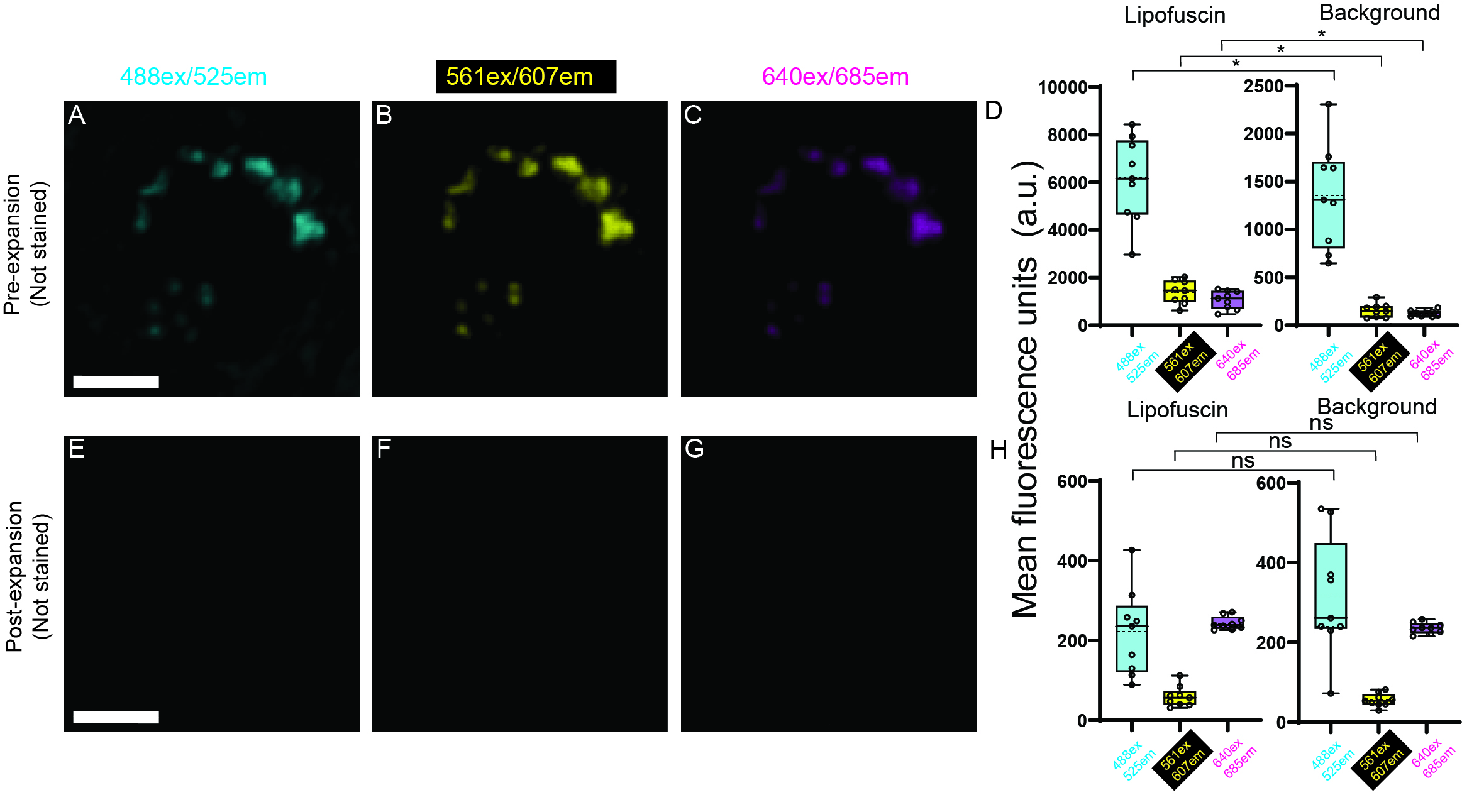

Supplement: Figure S6 [file NIHMS1967379-supplement-Figure_S6.jpg]

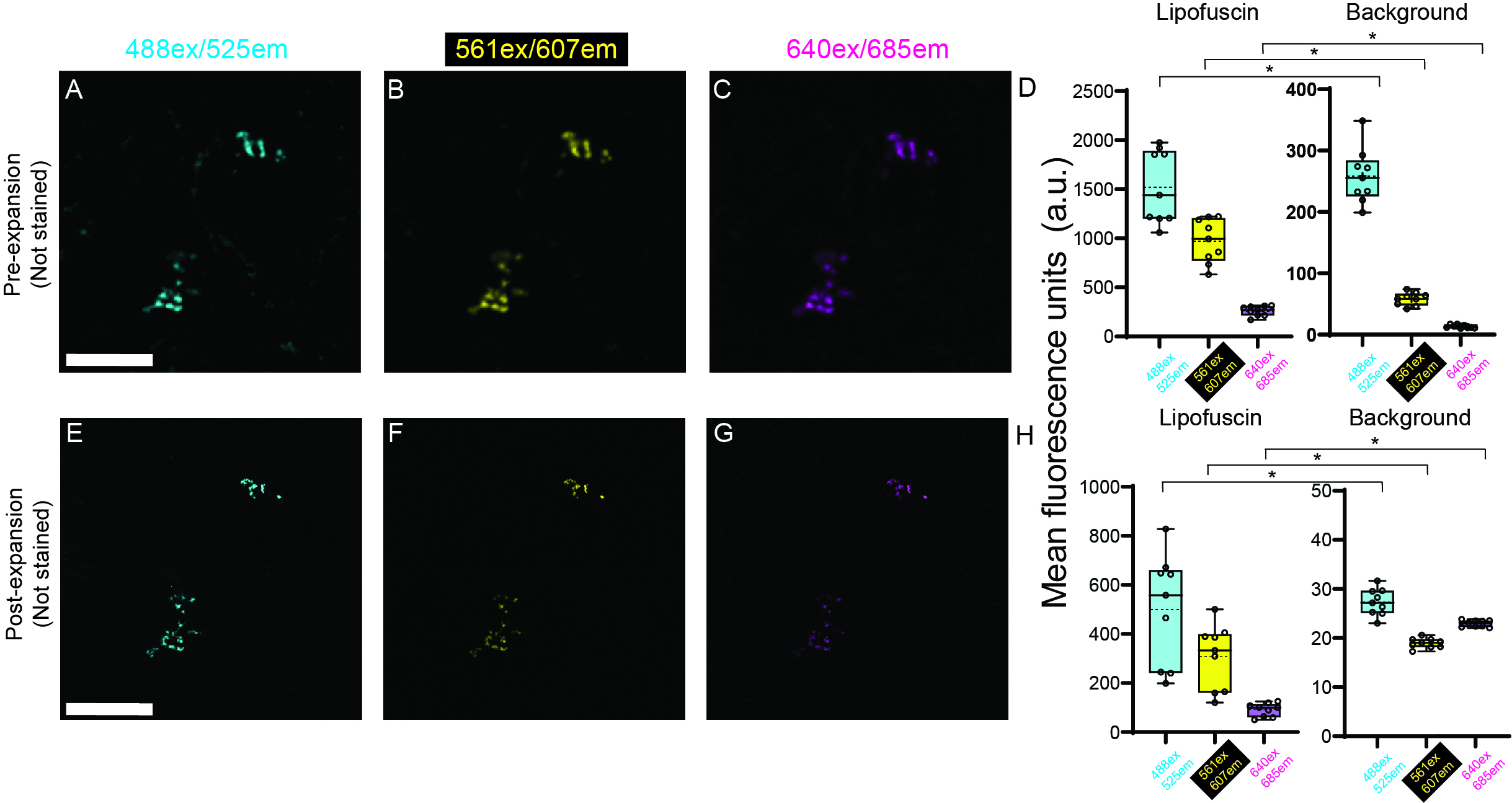

Supplement: Figure S7 [file NIHMS1967379-supplement-Figure_S7.jpg]

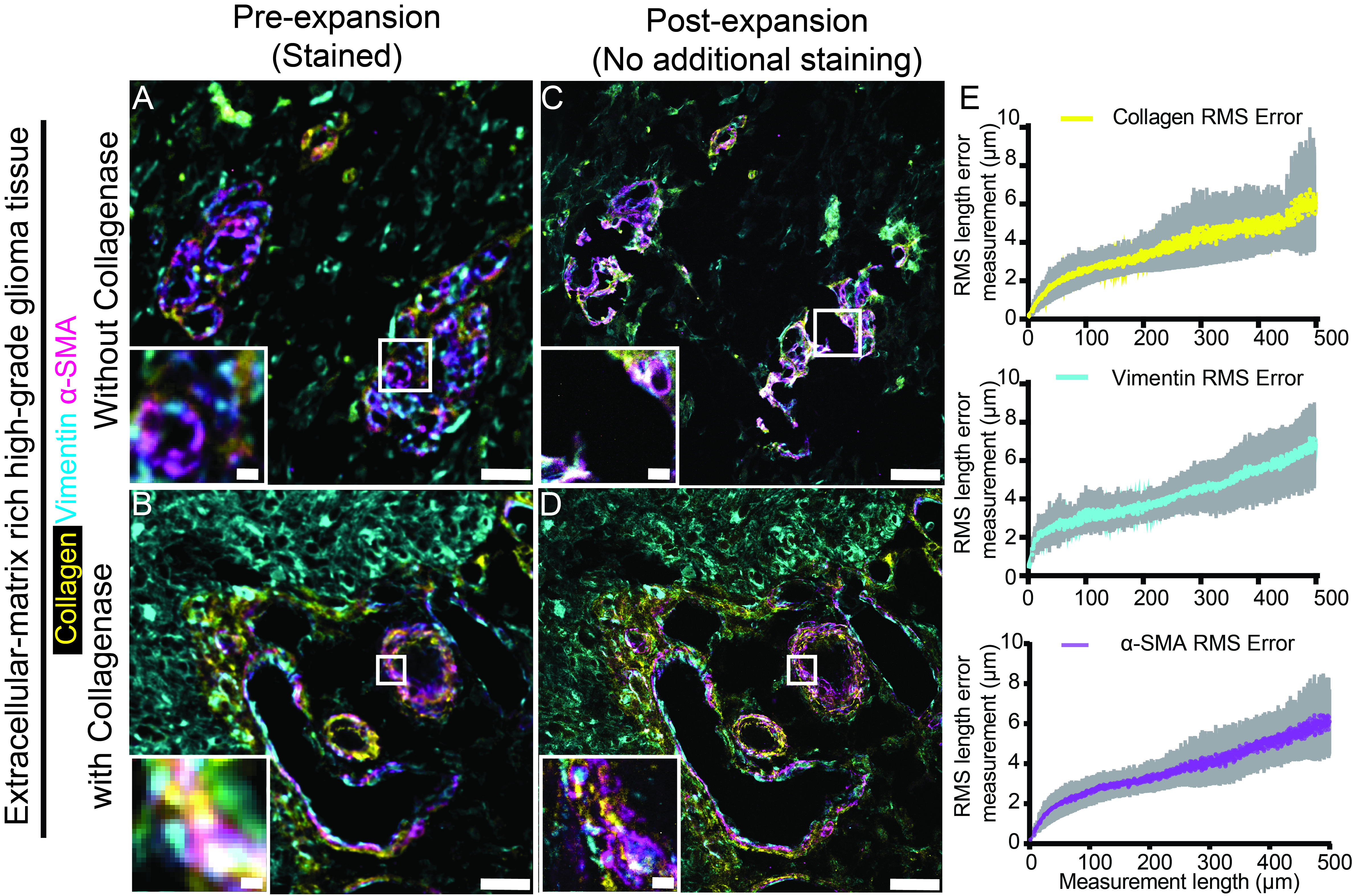

Supplement: Figure S5 [file NIHMS1967379-supplement-Figure_S5.jpg]

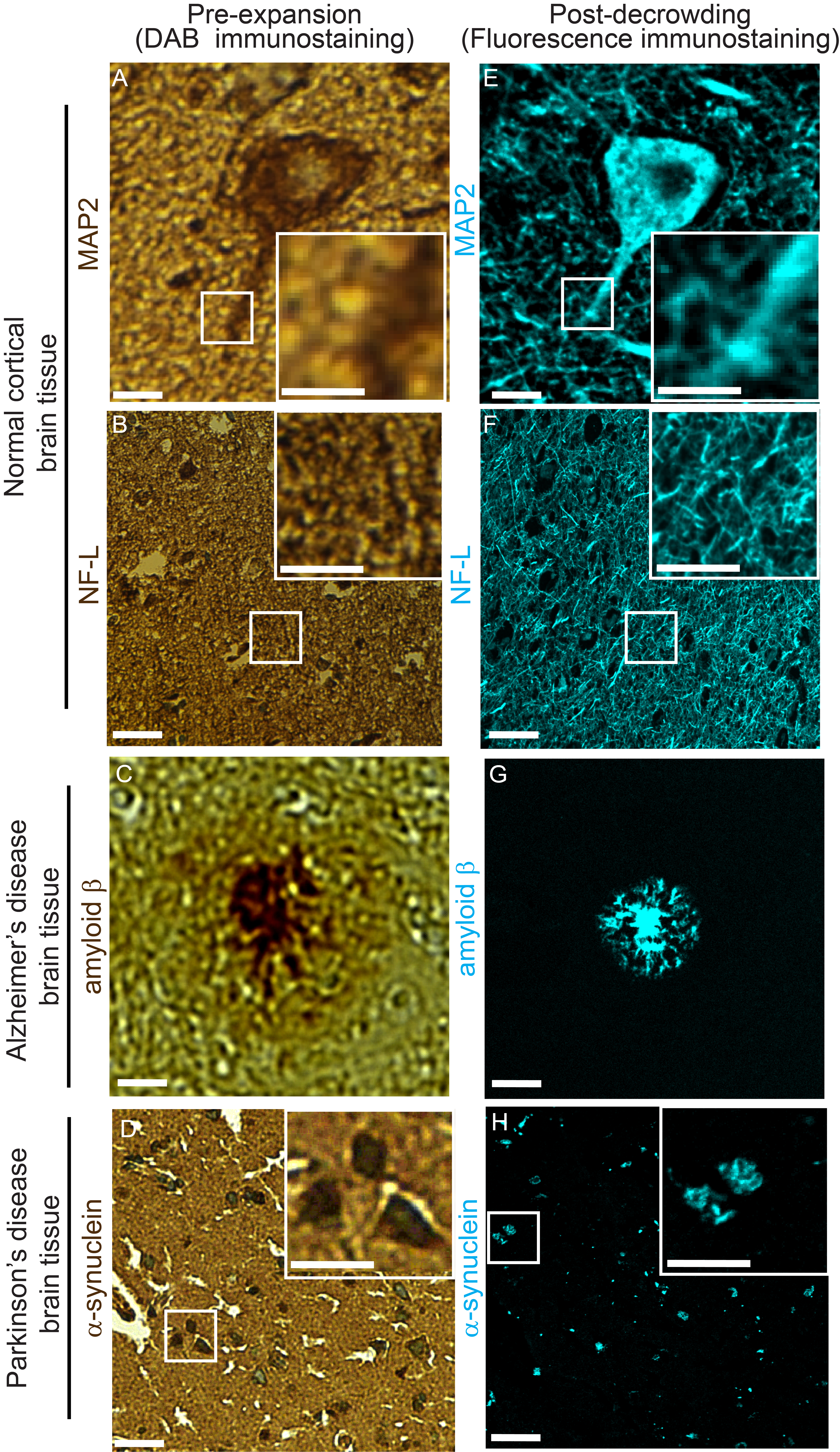

Supplement: Figure S8 [file NIHMS1967379-supplement-Figure_S8.jpg]

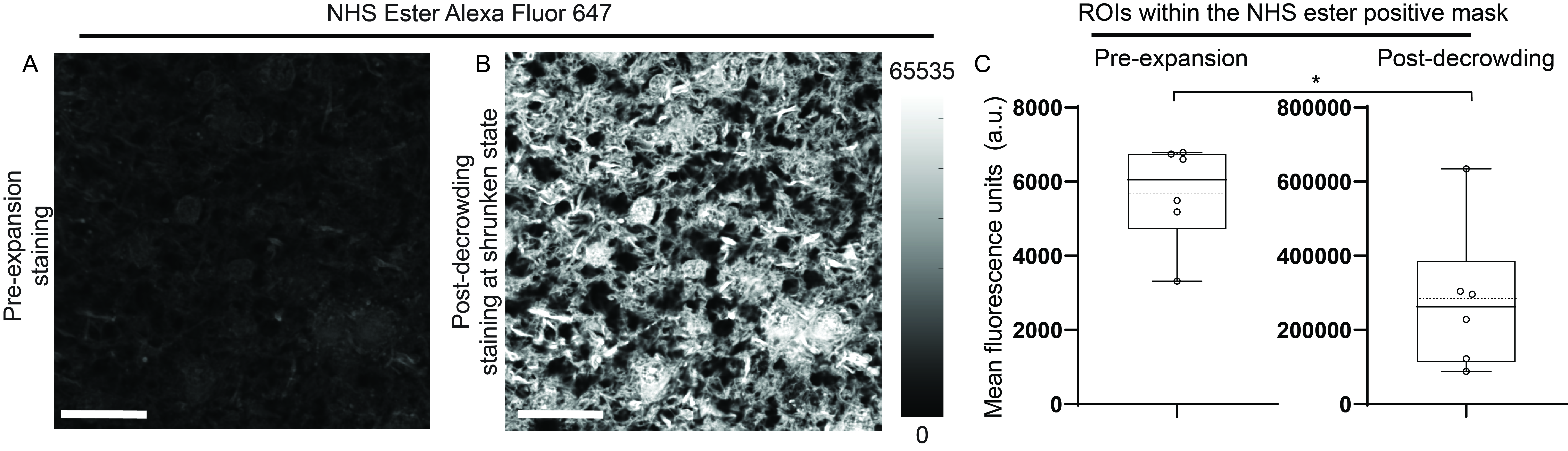

Supplement: Figure S10 [file NIHMS1967379-supplement-Figure_S10.jpg]

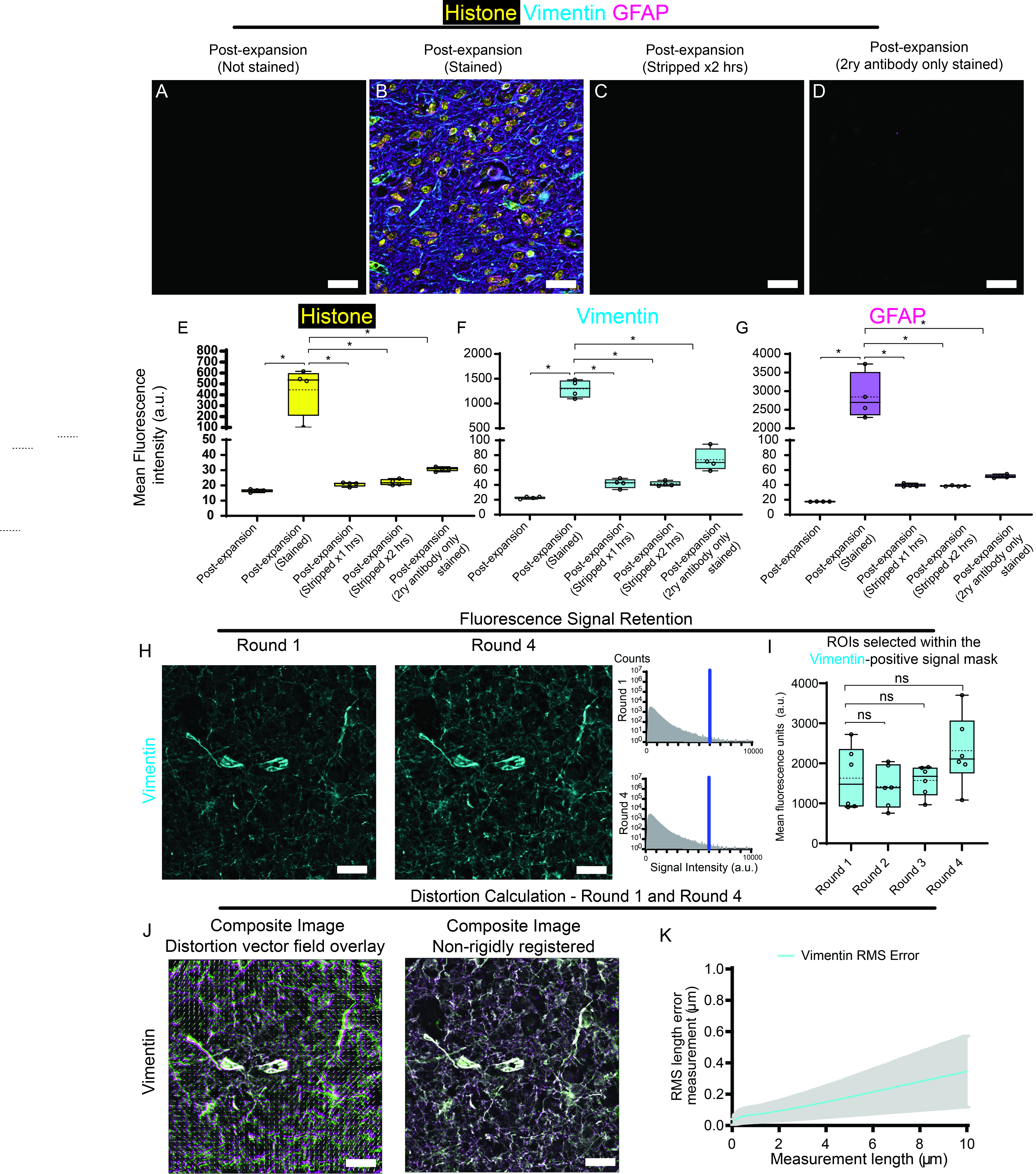

Supplement: Figure S11 [file NIHMS1967379-supplement-Figure_S11.jpg]

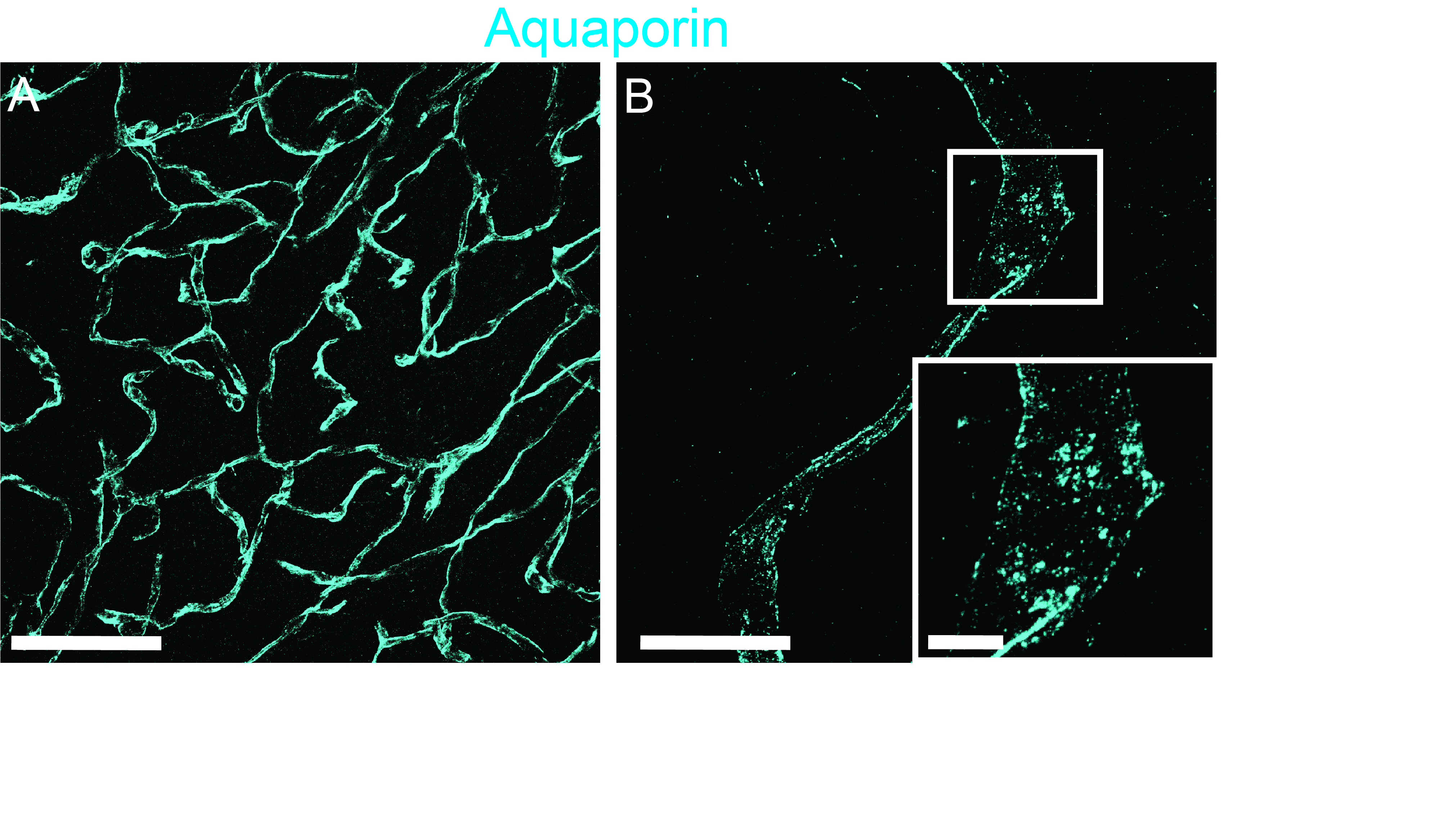

Supplement: Figure S12 [file NIHMS1967379-supplement-Figure_S12.jpg]

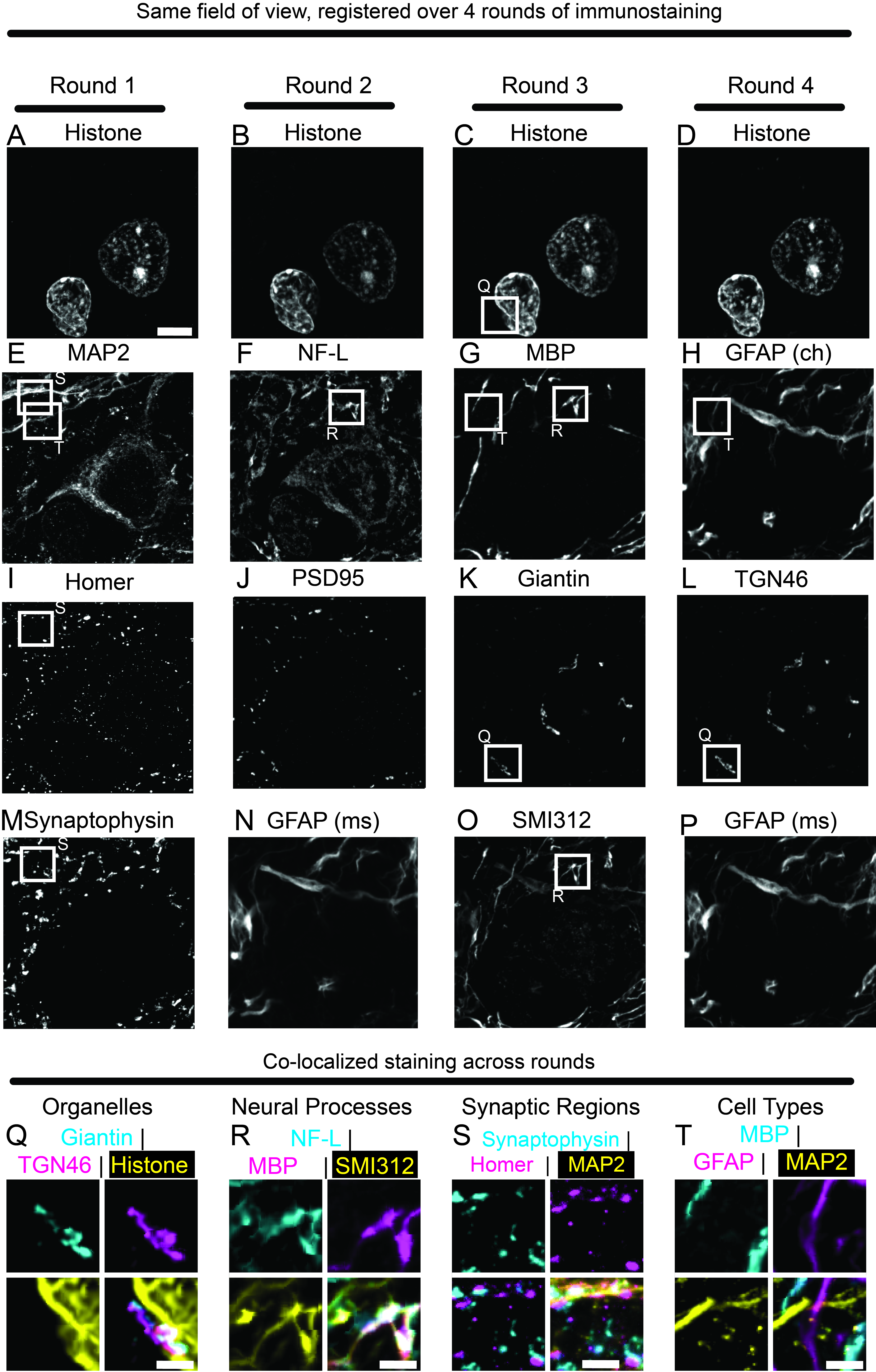

Supplement: Figure S14 [file NIHMS1967379-supplement-Figure_S14.jpg]

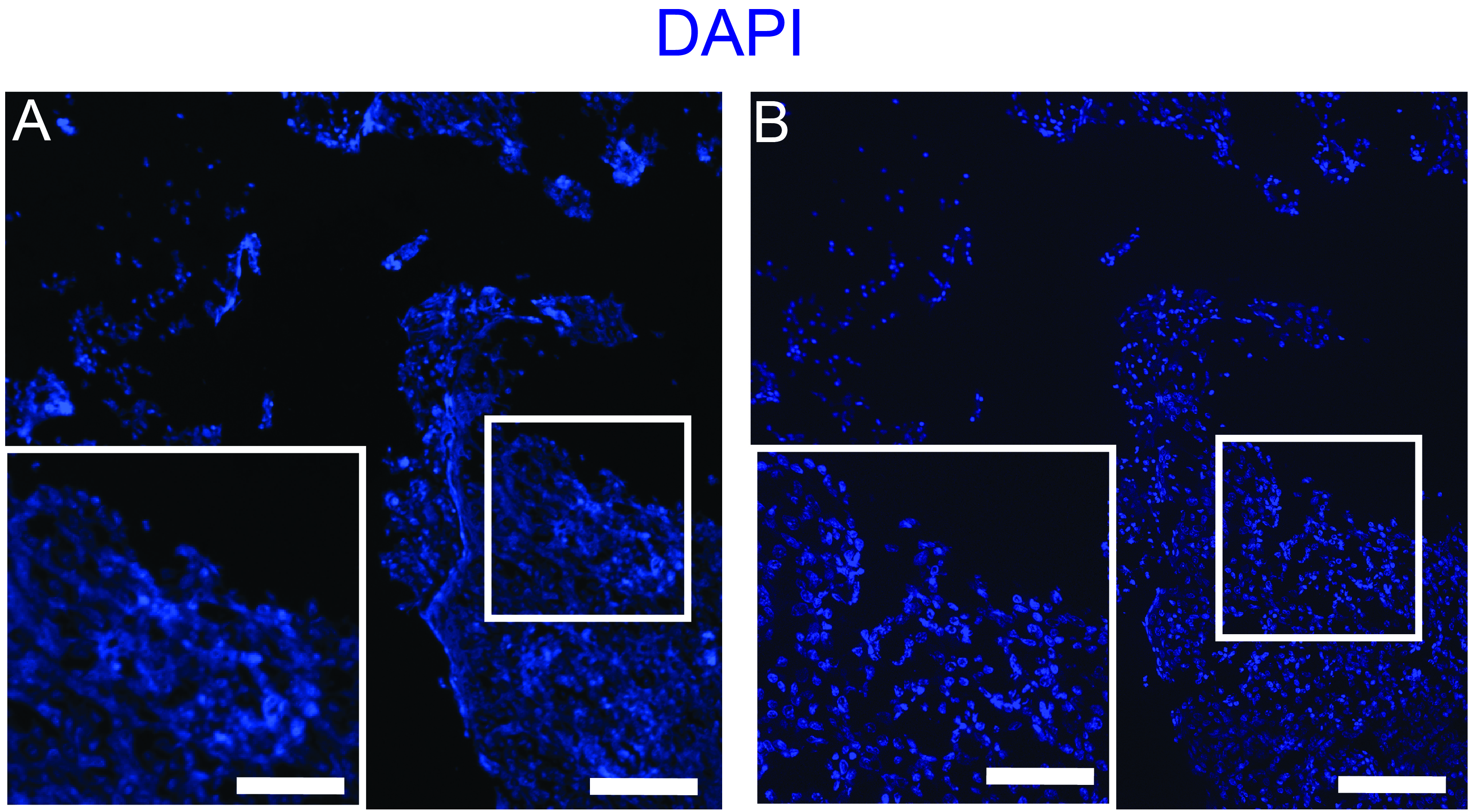

Supplement: Figure S15 [file NIHMS1967379-supplement-Figure_S15.jpg]

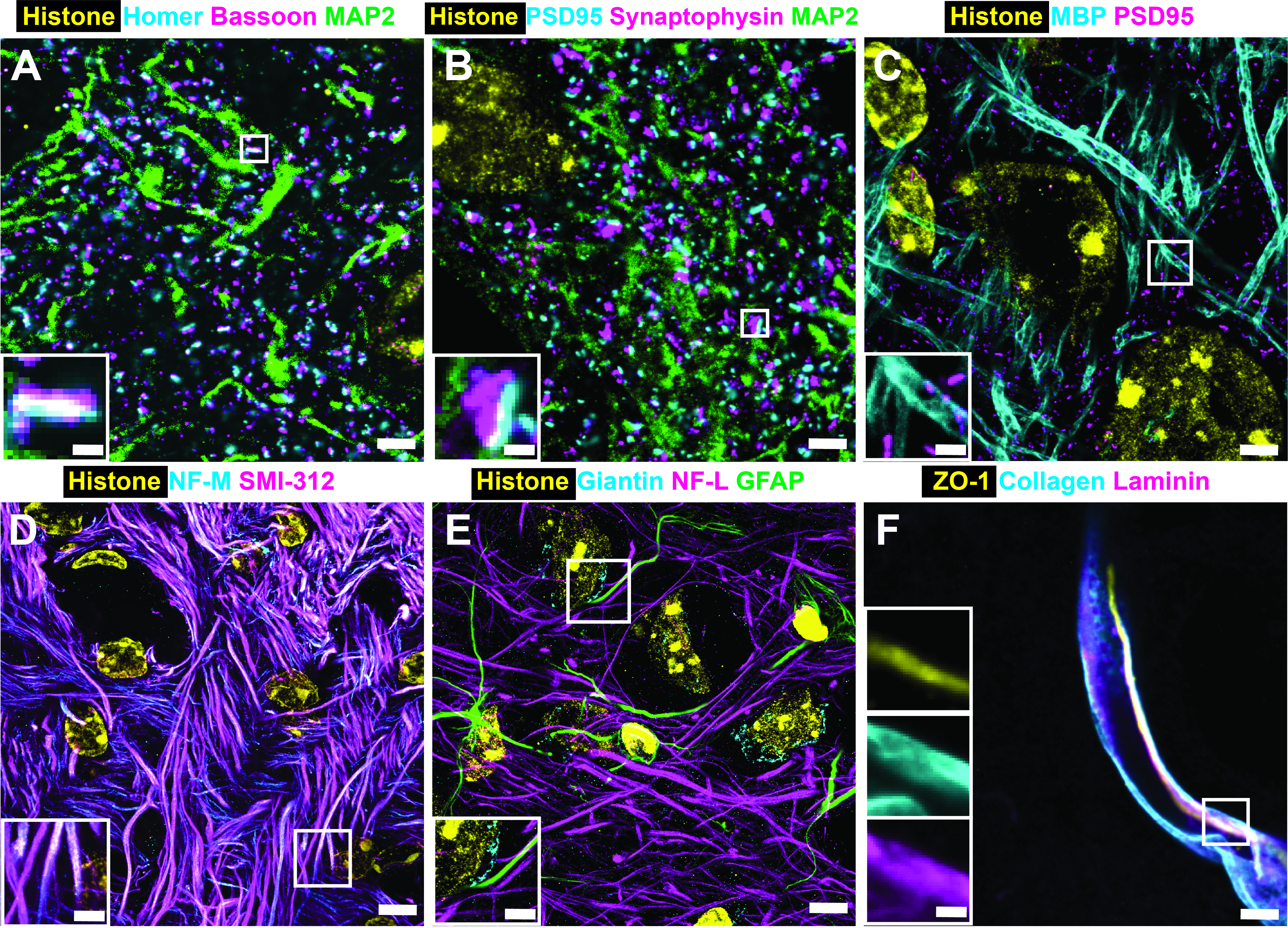

Supplement: Figure S13 [file NIHMS1967379-supplement-Figure_S13.jpg]
