# Supplementary material for: Improved immunostaining of nanostructures and cells in human brain specimens through expansion-mediated protein decrowding
Source: Sci Transl Med. Author manuscript; Available in PMC 2024 Mar 4. (PMC10911838; doi:10.1126/scitranslmed.abo0049)
Supplement: Protocol [file NIHMS1967379-supplement-Protocol.docx]

**Decrowding Expansion Pathology**

This protocol is designed for 5-10 µm thick brain sections of human formalin-fixed paraffin embedded (FFPE) tissues or 5-10 µm thick brain mouse tissues fixed in 4% paraformaldehyde (PFA). Incubation times need to be adjusted for thicker tissues, e.g., 100 µm thick. For the reader’s guidance, we have provided additional details for use in thicker mouse tissues.

**A. STOCK SOLUTIONS (Anchoring, Gelation)**

First, prepare the stock solutions and store in aliquots at -20°C for long term storage. We found that storing these solutions in 1-1.5 mL aliquots was ideal for use when preparing 1-20 tissue gels per day, 3-5 times per week.

**1. Monomer solution:**

| **Table 1. Monomer Solution Composition** | | | | |
| --- | --- | --- | --- | --- |
| **#** | **Component** | **Stock concentration*** | **Amount (mL)** | **Final concentration*** |
| **1** | Sodium acrylate | 38 | 2.25 | 8.6 |
| **2** | Acrylamide | 50 | 0.5 | 2.5 |
| **3** | N,N′-Methylenebisacrylamide | 2 | 0.50 | 0.10 |
| **4** | Sodium chloride | 29.2 | 4 | 11.7 |
| **5** | Phosphate buffered saline (PBS) | 10x | 1 | 1x |
| **6** | Water |  | 1.15 |  |
|  | **Total** |  | **9.4**** |  |

*All concentrations are in g/100 mL in ultrapure deionized water except phosphate buffered saline (PBS) 10x. Remember, these are weight by volume (w/v) concentrations, so make sure, for example, when making the acrylamide stock solution, you have 2.5 g added to the vial and then fill up the vial to the 100 mL mark, or 0.25 g and fill up the vial to the 10 mL mark. DO NOT add 10 mL and then 0.25 g of solid or else your final concentration will be incorrect.

**9.4/10 mL with the remaining 6% volume brought up by initiator, accelerator and inhibitor.

Prepare the monomer solution at room temperature (RT). In a 10 mL conical tube add each component (**Table 1**) #1-6 in sequential order. After adding each component, vortex the components to ensure they are in solution. Once you have mixed all the components, dispense into 1-1.5 mL aliquots and then store at -20°C for long term storage.

**2. Inhibitor solution**

Prepare the 4-hydroxy-TEMPO (**4HT)** stock solution at RT made up at 0.5% w/v in water and then dispense into 1-1.5 mL aliquots.

**3. Accelerator solution**

Prepare the tetramethylethylenediamine (**TEMED)** stock solution at RT made up at 10% w/v in water and then dispense into 1-1.5 mL aliquots.

**4. Initiator solution**

Prepare the APS (**APS)** stock solution at RT made up at 10% w/v in water and then dispense into 1-1.5 mL aliquots.

*TEMED, APS, and 4HT stock solutions can be kept at -20°C for at least 6 months.

**5. Anchoring solution**

Acryloyl-X, SE (Life Technologies, A20770) (**AcX**) comes in a solid powder. Prepare the AcX stock solution by adding 500 µL anhydrous dimethylsulfoxide (DMSO) into the original container resulting in a 10 mg/mL stock solution, dispense into 20 µL aliquots and store in a desiccated environment at -20°C for long term storage.

**5. PBS 1x in Triton-X (PBST)**

Prepare a PBST stock solution at RT made up of PBS 1x with 0.5% Triton-X (v/v) and store at RT in 10 mL conical tube. Make fresh every week.

**B. BUFFERS (Softening, Immunostaining)**

| **Table 2. Softening Buffer Composition (pH = 8.0)*** | |
| --- | --- |
| **1** | 50 mM Tris buffer |
| **2** | 20% wt/vol Sodium dodecyl sulfate (SDS) |
| **3** | 25 mM Ethylenediaminetetraacetic acid (EDTA) |
| **4** | 0.5% Triton-X |
| **5**** | 100 mM beta-mercaptoethanol (BME) |

*Buffer can be stored at room temperature. Should be made fresh regularly every week without beta-mercaptoethanol (BME) (**Table 2**).

**IMPORTANT: Use a 14M stock solution of BME and add immediately prior to use with tissue to achieve a final concentration of 100 mM BME in softening buffer. DO NOT store Softening buffer with BME.

Prepare Softening buffer by first weighing out the solid SDS powder and adding into a glass container with a magnetic stirrer. Then, add deionized water to below the desired volume to leave sufficient room for the Tris, EDTA and Triton-X. Stir at a temperature of approximately 90°C. Then, add the Tris buffer, using a stock solution of Tris. We use a 1 or 2 M Tris buffer stock solution. Then add the EDTA, using a stock solution of 500 mM EDTA. Allow components to mix well so they all go into solution, and once that has occurred add the Triton-X. Then adjust pH = 8.0 by slowly adding stock concentrated hydrochloric acid. Then store the Softening buffer WITHOUT the BME at room temperature. Add the BME immediately prior to using with tissue.

**Collagenase Buffer** (make fresh each time):

| **Table 3. Collagenase Buffer Composition*** | |
| --- | --- |
| **1** | Hanks' Balanced Salt Solution (1x),(HBSS), with calcium, with magnesium, no phenol red (Gibco, Thermofisher #14025134) |
| **2** | Collagenase II 1500 U/ml (Gibco, Thermofisher #17101015) |

Prepare Collagenase buffer by adding HBSS 1x stored at RT into the collagenase container to achieve a final concentration of Collagenase II of 1500 U/mL (**Table 3**). No not store but make fresh each time.

| **Table 4. Immunostaining Buffers** | |
| --- | --- |
| **1** | **Blocking buffer**: MAXblock™ Blocking Medium (Active Motif) |
| **2** | **Staining buffer**: MAXbind™ Staining Medium (Active Motif) |
| **3** | **Washing buffer**: MAXwash™ Washing Medium (Active Motif) |

We used these commercial immunostaining buffers for blocking, staining, and washing. However, the protocol can be used with any conventional immunofluorescence protocol, e.g., use of normal goat serum rather than the blocking buffer from Active Motif.

**C. PROTOCOL: Decrowding ExPath Protocol for Fixed Human Tissues**

**Step 1. FORMAT CONVERSION**

*For formaldehyde-fixed paraffin-embedded (FFPE) clinical samples*

1. Completely immerse the tissue slide sample (herein, “slide”) through a series of solutions, by sequentially immersing the slide for three (3) mins in each solution in the following order (**Figure 1**): xylene 🡪 xylene 🡪 50/50 solution of xylene/100% ethanol 🡪 100% ethanol 🡪 95% ethanol 🡪 90% ethanol🡪 80% ethanol 🡪 50% ethanol 🡪 deionized water 🡪 deionized water. All steps are done at RT. All ethanol dilutions from 95% down to 50% are diluted with deionized water.

2. Remove any excess deionized water from around and on top of the tissue by air drying and using a Kim wipe.

*Removing excess deionized water ensures that the anchoring solution is not diluted.

3. Place in 1x PBS for 5 min and then remove any excess PBS from around and on top of the tissue by air drying and using a Kim wipe.

*For 4% paraformaldehyde (PFA) samples*

1. Completely the immerse tissue slide three (3) times in 1x PBS for 5 min each time at RT.


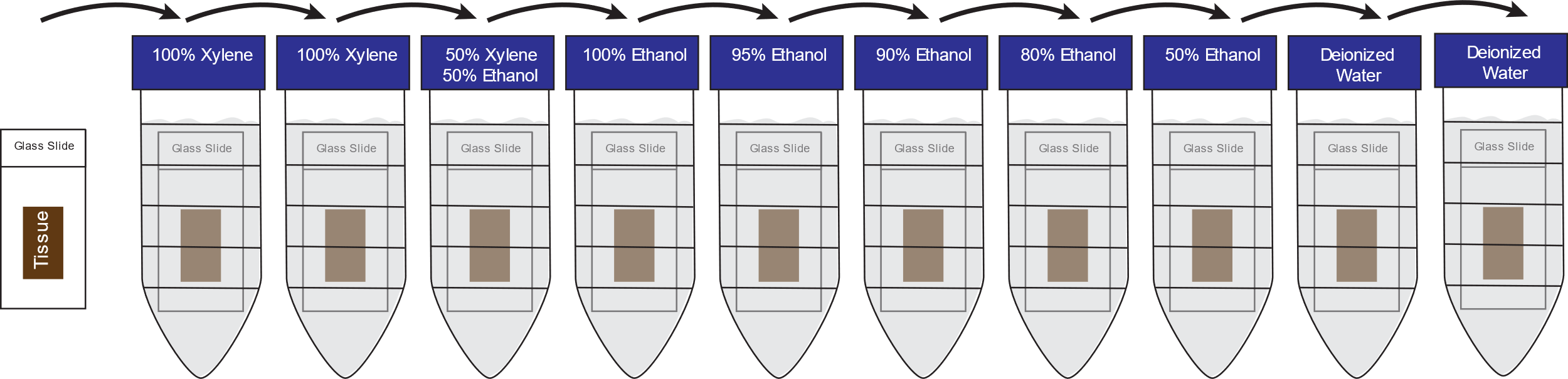


**Figure 1. Deparaffinization and rehydration.** Tissue glass slide with FFPE tissue (brown) is sequentially transferred starting in xylene through subsequent solutions of varying ethanol concentration and eventually to deionized water to deparaffinize and rehydrate tissue prior to anchoring and gelation.

****Pre-Expansion Staining:** If the user wishes to do pre-expansion staining for comparison between pre-expansion staining and post-expansion staining on the same tissue, please see below section **Pre-Expansion Staining.** The user would then proceed from **Step 1** to **Step 1-Extra: Pre-Expansion Staining.**

**Step 2. ANCHORING & GELATION**

*Anchoring*

1. Use the 10 mg/mL aliquot of AcX stock solution and allow it to come to RT.

2. Dilute stock AcX solution to a final concentration of 0.1 mg/ml using stock solution of PBST to prepare the working AcX solution.

3. To each slide, add 200 - 500 µL of working AcX solution.

*It is important to make sure the tissue is fully covered with working AcX solution.

4. Incubate slides for 30 min at RT and then transfer the slides for another 1.5 hours to a temperature of 37°C

*Incubate slides in a humidified closed container.

**If desired, this reaction can be run overnight at 4°C and then for 1 hour at 37°C to ensure complete gelation.

***For 50-100 µm thick PFA fixed mouse brain slices do an overnight incubation at 4°C and then 1 hour at 37°C to ensure complete anchoring.

*Gelation*

1. Remove the AcX solution from the slide by washing three (3) times in 1x PBS for three (3) min each wash

2. Remove any excess PBS from around and on top of the tissue by air drying and using a Kim wipe.

*Removing excess PBS ensures that the gelling solution is not diluted

3. Assemble a gel chamber by first sandwiching the tissue between the slide and a coverslip, with spacers on either side of the tissue section to prevent compression of the tissue (**Figure 2)**.

*Spacers are made from cut coverslips using a diamond knife.

**For most human tissue sections in clinical settings (5-10 µm thick), one piece of cover glass (VWR micro cover glass, 24x60mm, No. 1.5) can be used for spacers and a whole, uncut cover glass for the top cover glass.


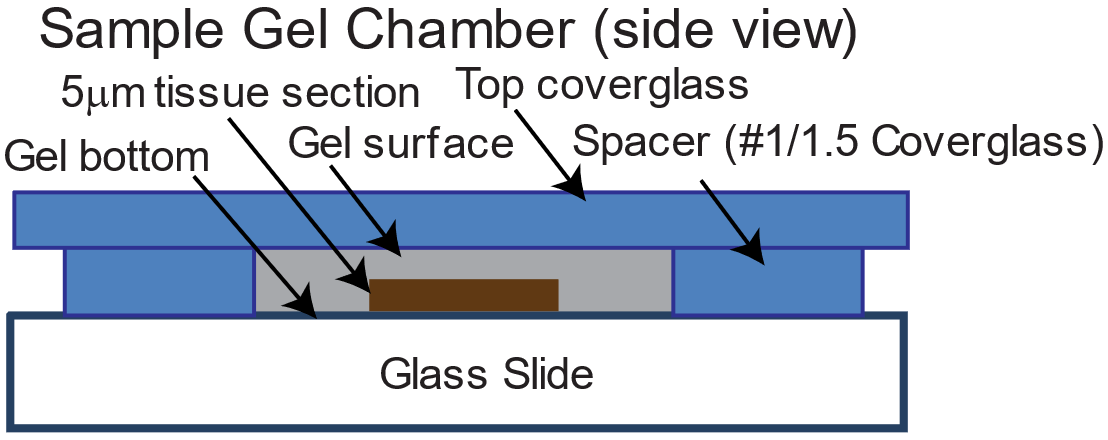


**Figure 2. Gel Chamber.** Gel chamber for gelation of anchored tissue. The tissue is positioned between the glass slide and the top cover glass, with cover glass spacers on each end to allow for space for the gelation solution to enter the chamber to form the gel and without compressing the tissue.

4. Make the gelling solution (**Table 5**) by mixing 4 solutions in the following order: a) monomer solution, b) TEMED (accelerator), c) 4HT (inhibitor), d) APS (initiator). The mixture should be vortexed to ensure full mixing.

***HT** which inhibits gelation to enable diffusion into tissue sections.

****TEMED** accelerates radical generation by APS.

*****APS** initiates the gelling process, so it needs to be added last to prevent premature gelation.

| **Table 5. Gelling Solution Composition** | | | |  |
| --- | --- | --- | --- | --- |
| **#** | **Component** | **Amount of stock solution (µL)** | **Dilution ratio** | **Final concentration (w/v)** |
| **1** | Monomer solution | 187 | N/A | N/A |
| **2** | 4HT (inhibitor) | 4 | 1:50 | 0.01% |
| **3** | TEMED (accelerator) | 4 | 1:50 | 0.2% |
| **4** | APS (initiator) | 5 | 1:50 | 0.2% |
|  | **Total (µL)** | **200** |  |  |

5. Add gelling solution into the gelling chamber by using a micropipette with a 200 µL tip.

a) THIN sections, 5-10 µm thick: Use freshly prepared gelling solution (adding APS at the very end). Make sure at least a 100-fold excess volume of monomer solution is used, e.g., ~200 µl of gelling solution for each tissue section on the slide, and then incubate for 30 min at 4°C in a humidified environment followed by 2.5 hours at 37°C.

*You can also perform the incubation overnight at 4°C followed by 1.5 hours at 37°C.

b) THICK sections, 50-100 µm thick. Use freshly prepared gelling solution (without APS) making sure the whole tissue section is immersed in the solution and incubate at 4°C in a humidified environment overnight. Then remove excess gelling solution (without APS) and add fresh gelling solution (with APS) and incubate for 30 min at 4°C in the gel chamber and then for 2.5 hours at 37°C.

6. Once the sample is gelled you can proceed to softening and decrowding (below), or storage by placing the slide chamber at 4°C inside a humidified container such as a Petri dish with a damp Kim wipe (or other container as appropriate) sealed with Parafilm for storage. You can also cut out the tissue gels and transfer into an airtight Eppendorf tube for later use.

- 1. For clinical FFPE tissues that are tightly attached to the charged slide: in those cases, the user can store the whole slide as noted above until softening.
  2. For tissues attached to non-charged slides: generally, the user can gently remove tissues with a single edge razor blade (Fisher Scientific 17-989-126) or a brush to store separately.

**Step 3. Softening**

1. Take off the top cover glass of the gel chamber using a razor blade placed at the edge of the coverslip, sliding the blade along the coverslip side touching the gel surface and then gently using the blade to lift the coverslip off the gel surface.

2. Trim the tissue-containing-gel, keeping away from the tissue but removing excess gel to minimize volume by using a sharp razor blade, and cut a corner in an off-angle fashion to keep track of the orientation of tissue for later steps (e.g., when the gel is transparent, and orientation is difficult to ascertain). This step will help determine the gel surface and the gel bottom (**Figure 3**).


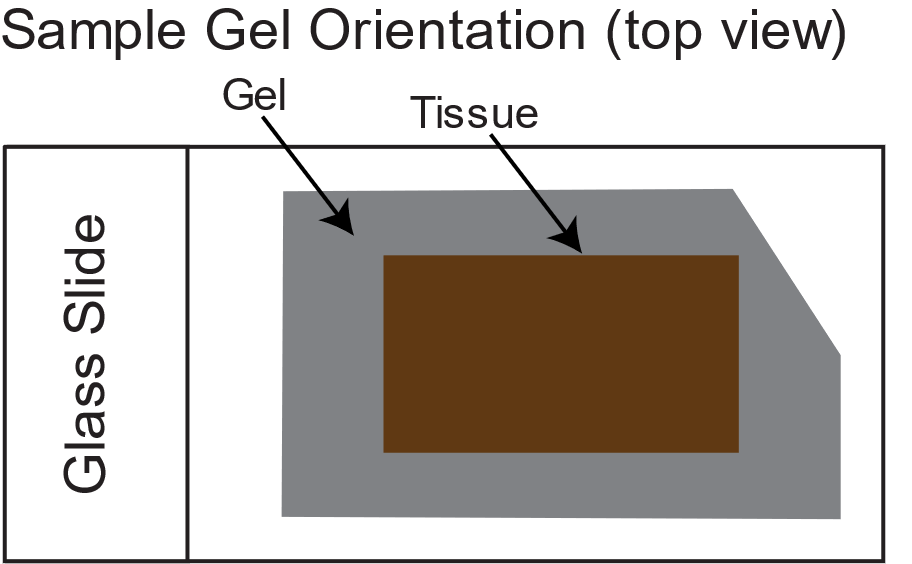


**Figure 3. Gel Trim and Orientation.**  Gelled tissue is trimmed after removing the gelation chamber. A cut in an off-angle fashion is made to help the user remember the orientation of the original tissue and distinguish the gel bottom from the gel surface.

3. This is particularly useful when using objectives with a short working distance, to ensure the gel surface is facing down towards the objective (**Figure 4**).


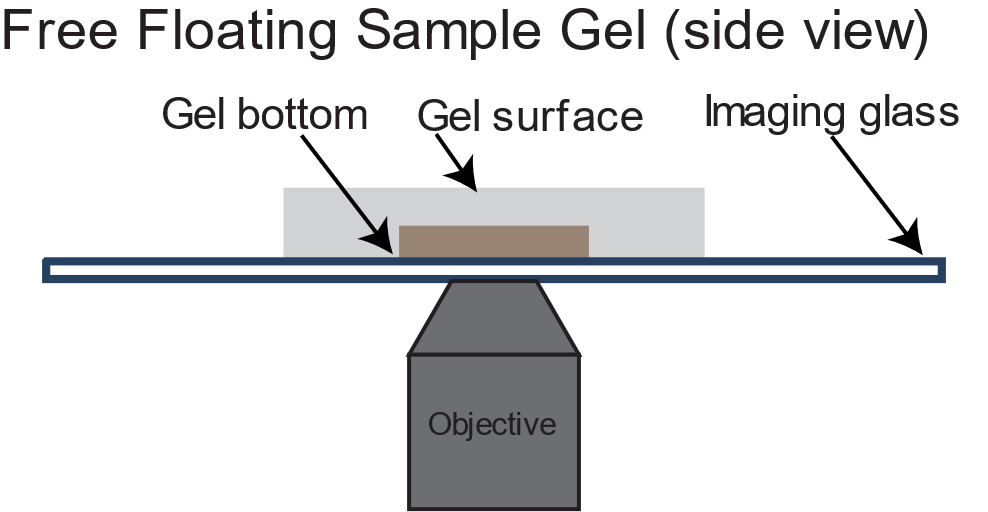


**Figure 4. Gel surface and bottom orientation.** In short distance objectives, it is especially important to remember the gel surface and bottom to be able to adequately image tissue at super-resolution scales.

4. ***This step (4) can be skipped in many brain tissues, including normal brain and mouse tissues. This is particularly useful in tissue with significant amounts of vascular and extra cellular matrix components found in brain pathologies like certain types of vascular brain tumors. Submerge the tissue sample slide into a container ensuring the tissue is fully covered with Collagenase buffer and incubate for 3 hours at 37°C with gentle shaking. Then transfer the slide with gel to a new container containing Softening buffer.

5. Incubate the gelled tissue in Softening buffer at 37°C for 30 min with gentle shaking and then incubate for 1 hr in a steamed autoclave at 121°C (**Figure 5**).

*For FFPE tissues in glass slides, add the full tissue slide into a conical tugbe, as the gelled tissue will separate from the glass slide with the least disruption of the tissue and into the bottom of the tube following softening.

**For PFA tissues on a non-charged slide and that have been removed from the slide, they can be added as free floating tissue.


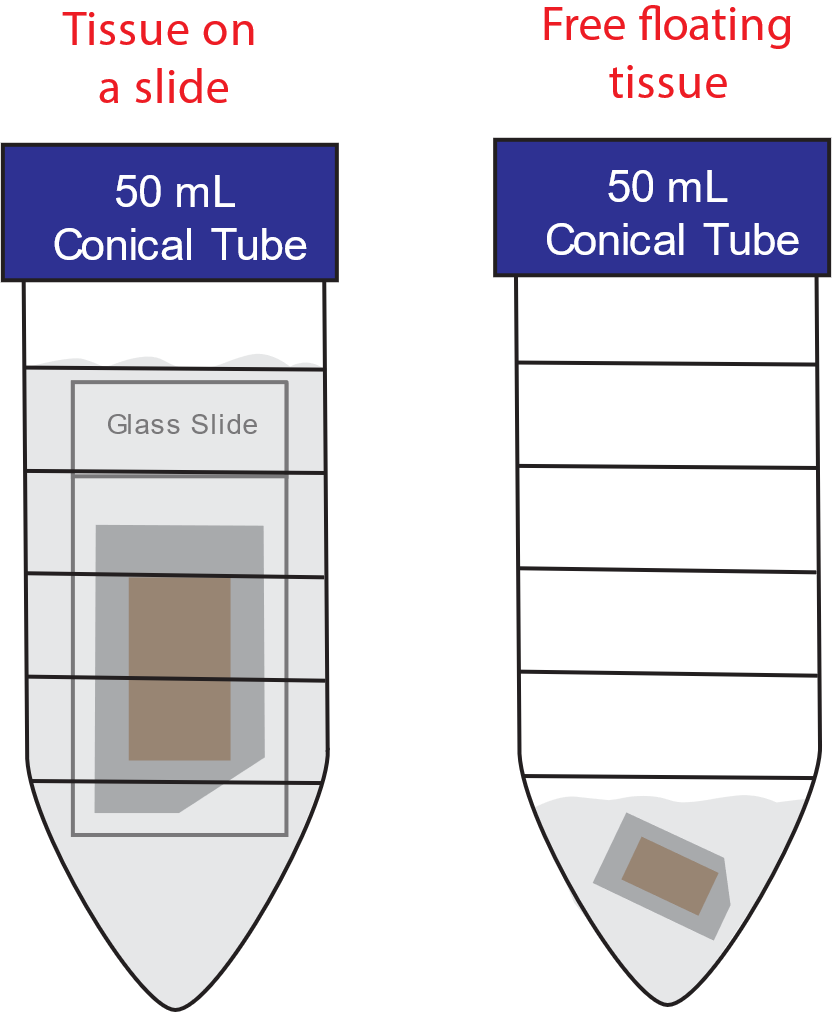


**Figure 5. Softening Buffer Incubation.** Two examples of how to perform the softening step on tissues attached to a slide or on free floating tissues

- 1. You can submerge the gel, still on the slide (or the free floating gel if it has been separated from slide) meanwhile ensuring tissue is fully covered with freshly made softening buffer (including BME).
  2. Small tissues can be added into an Eppendorf tube; full slides can be added into appropriate containers that ensure sealing like a 50 mL conical tube which would then have the tissue fall off the slide.

6. Normally the sample will detach from the glass slide by itself after softening with gentle shaking. If needed, use a razor blade or brush to gently move the sample off the slide. However,it has been our experience that FFPE tissues will fall off at the end of treatment or during the cool down period with gentle shaking.

*For 50-100 µm thick PFA fixed mouse brain slices do 48 hours (overnight x2) at 37°C with gentle shaking in Softening buffer (including BME) and then, remove old buffer, add new buffer, and incubate for another 1 hr in a steamed autoclave cycle, cool down, and wash in 1x PBS five (5) times at least 45-60 min each wash, ensuring complete removal of softening buffer.

**Step 4. Decrowding**

1. After softening, the gelled tissue has detached from the slide and is floating freely in the Softening buffer.

2. Transfer the tissue into a clear polystyrene petri dish plate by slowly decanting the buffer solution which contains the gelled tissue into the plate.

3. Using a pipette, the excess buffer is removed and discarded.

4. Add 1x PBS to the well plate to fully cover the tissue and the petri dish plate is gently shaken at RT to remove excess softening buffer.

5. While the gelled tissue is free floating in 1x PBS use a flat, wide, miniature paintbrush and place underneath the gelled tissue (**Figure 6**), ensuring that the paint brush is covering most of the gelled tissue undersurface area and transfer into a clear 6-well plate (Clearstar) that contains 1x PBS to completely submerge the tissue

6. Gently shake the well plate at RT for 3 min.

7. Excess 1x PBS is removed using a pipette and new 1x PBS is added to cover the tissue and the well plate and gently shake at RT for 3 min. This process is repeated a total of 5 times, which results in tissues reaching an expansion factor of ~2.3x.

8. While the gelled tissue is free floating in 1x PBS, use a flat, wide mini paintbrush (i.e., a brush with no prior contact with softening buffer) and place underneath the gelled tissue, ensuring that the paint brush is covering most of the gelled tissue undersurface area, and transfer into a new 6-well plate (CellVis) that will be subsequently used for imaging, and which contains 1x PBS.

*Once the gelled tissue has been washed thoroughly in 1x PBS they can be stored in this state for days to weeks if the PBS does not evaporate, otherwise, a dry environment will dry out the gels and the tissue gel will be unusable.


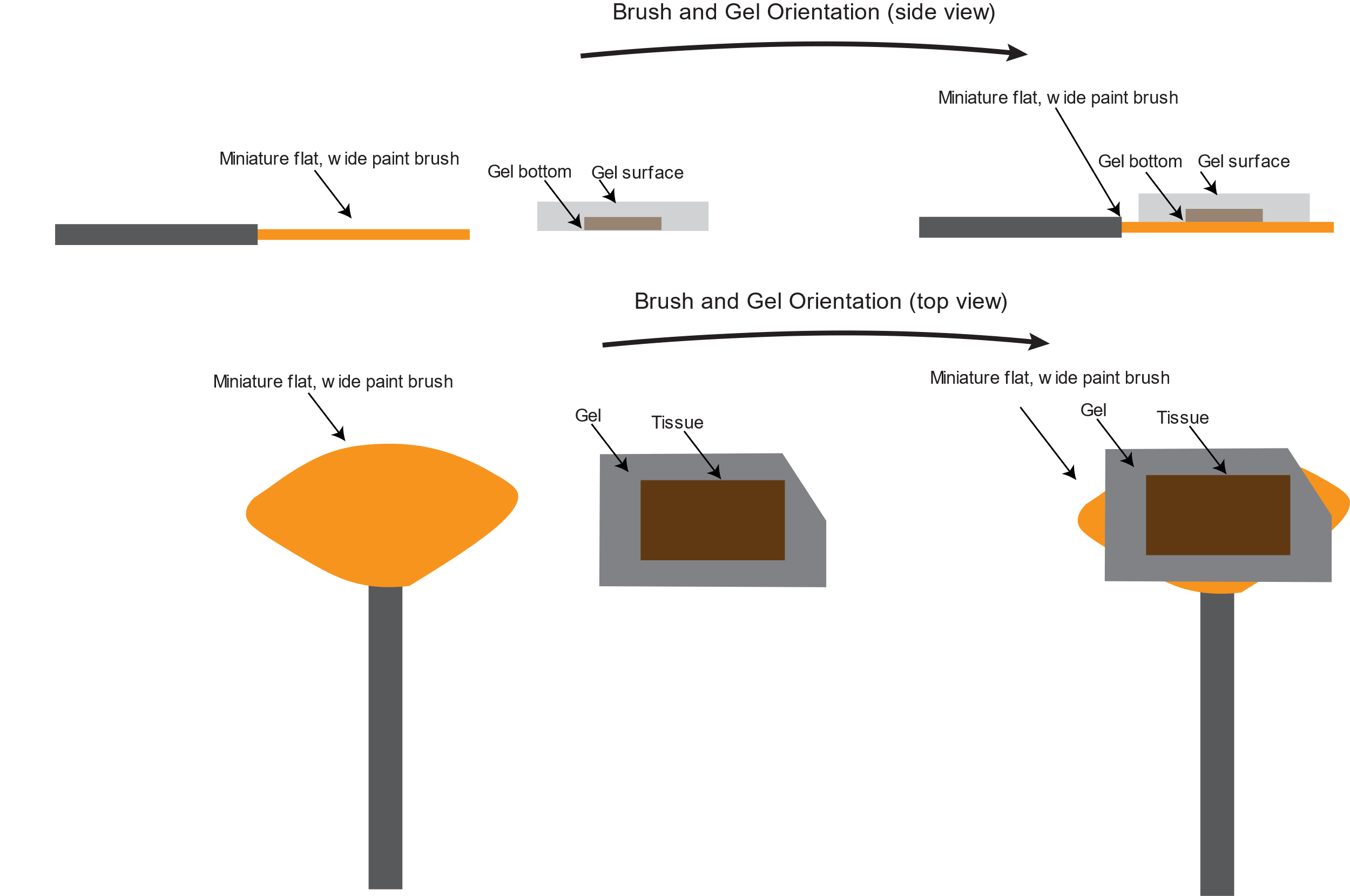


**Figure 6. Gel Transfer.** Miniature flat, wide paintbrush (orange) placed underneath the gelled tissue while it is floating in solution. Transfer on and off the pain brush while tissue is in solution to ensure no damage to the gelled tissue.

**Step 5. Immunostaining Post-Decrowding**

*The following steps are like a typical staining protocol (immunofluorescence (IF)/immunohistochemistry (IHC)) but in this case “on a gelled tissue”. The numbers below are for a typical 5x5 mm tissue gel (i.e., the width and length size after the decrowding step) and in which the staining is done on a 6-well plate (CellVis). **Volumes can be modified if using smaller containers/samples, e.g., a tissue microarray core with an original diameter of 1 mm, after decrowding can be a 2.3x 2.3 mm square and can be used in a 12-well plate (CellVis) with glass bottom or transferred into 0.5 mL Eppendorf tubes, and volumes can go down to 100-200 µL for working antibody solutions.

***The incubation times here are for FFPE tissues with an original thickness of 5-10 µm. For 50-100 µm thick tissues we recommend at least 24 hours incubation times with both primary and secondary antibody incubations at 4°C.

*Primary Antibody Staining*

1. Remove excess PBS from the well, add 500 µL of MAXblock™ Blocking Medium and incubate for 1 hour at 37°C.

2. Remove excess MAXblock™ Blocking Medium and wash three (3) times with 1 mL each time using MAXwash™ buffer for 3 min each at RT.

3. Prepare primary antibody solution by diluting primary antibody in MAXbind™ Staining buffer.

4. Perform post-decrowding immunostaining by incubating tissue samples in 500 µL of primary antibody solution at 37°C for 1 hour, at RT for 2.5 hours or at 4°C overnight.

5. Remove excess primary antibody solution and wash three (3) times with 1 mL each time using MAXwash™ buffer for 3 min each at RT.

*Secondary Antibody Staining*

6. Prepare secondary antibody solutions by diluting secondary antibody in MAXbind™ Staining buffer.

*If using a nuclear stain (e.g., DAPI), staining can be done by incorporating the nuclear stain into the secondary antibody solution.

7. Incubate the tissue samples in 500 µL of secondary antibody solution using the same conditions applied for primary antibodies.

8. Remove excess secondary antibody solution and wash three (3) times with 1 mL each time using MAXwash™ buffer for 3 min each at RT.

9. At this stage tissues are ready for imaging at the 2.3x expanded state, ready for expansion to ~4x (see below), or for storage in excess 1x PBS.

*Suggested incubation periods and temperatures are given as a guide only. It is recommended that the user optimize these parameters for use in their own experiment based on antibodies and tissue thickness. It has been our experience that with a large range of commercially available antibodies and in tissues of this thickness, incubation times of 1-2 hours seem sufficient to get adequate staining.

**Sample needs to be covered with the well plate cover and sealed with Parafilm during the incubation periods to prevent drying out.

***Antibody concentrations are antibody specific and will require optimization based on the antibody used. It has been our experience that we start with the recommended concentrations by the vendor and then concentrate or dilute the solution as needed.

*Tissue Expansion*

10. Immunostained tissues are expanded by washing with deionized water at RT for 3-5 times for 3 min each at RT to achieve an approximate ~4x linear expansion.

*For expansion, remove the PBS and wash the samples with excess volume of 1/100 1x PBS for 3-5 times, for 10 minutes each time at RT. You can also use deionized water, but in post-expansion staining we have noticed a small decrease in fluorescence intensity of up to 10% when expanding in deionized water compared to a low salt solution like 1/100 PBS 1x. Gels will reach an expansion factor of ~4x.

**Slice expansion should reach a plateau after about the 3^rd^ or 4^th^ wash.

***Expansion chamber (i.e., well plate) needs to be of adequate size to fit the expanded sample.

***The sample might need to be trimmed prior to expansion using a razor blade into smaller pieces if no chamber of proper size can be obtained. In general, an expanded gel containing a tissue with diameter less than 0.6 cm pre-expansion fits adequately in a glass bottom 6-well plate.

****Please note that fully expanded gels are friable and can easily break if the user tries to lift them up. To transfer gels between containers do so in the ~2.3x state.

**Step 6. Imaging**

1. Remove any excess solution to ensure that the tissue sample is dry around all the edges using a micropipette to gently suction out any excess liquid as well as Kim wipes without touching and damaging the gelled tissue.

*Tissue samples can be imaged at super-resolution scales using conventional systems such as a wide field or confocal fluorescence microscope.

2. Make sure the gel bottom is facing down towards the objective (**Figure 7**).

*See step 4.2 for details regarding gel orientation.

**Experienced users can determine which side is the gel surface or gel bottom by the microscope z-settings when focusing on their tissues.

3. Once confirmed that the gel bottom is facing down, the tissue sample can be imaged.

*We found we can minimize micromovements to obtain high quality images at 10x or 40x magnification by placing a cut piece of cover glass of slightly larger surface area (i.e., larger than the gel) on top of the gel (**Figure 7**).

*When imaging is completed, the gel can be submerged in excess 1x PBS and the cover glass will separate from the gel without damaging it.


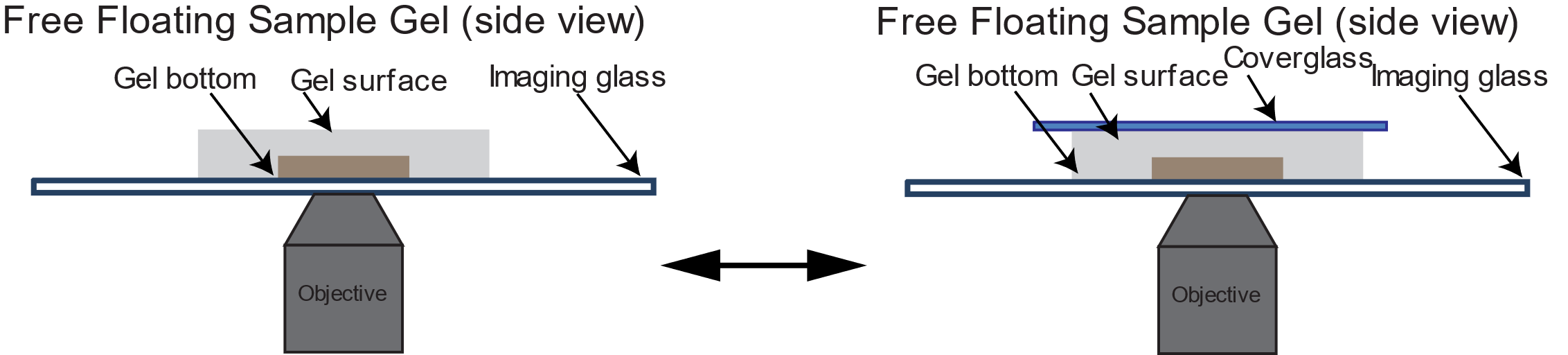


**Figure 7. Imaging of dExPath Gelled Tissues.** Imaging with an inverted wide field or confocal fluorescence microscope, with the gel bottom facing down towards the microscope objective, which is especially important when working with short working distance objectives. To ensure no movement of tissue after drying, place a cut cover glass on top of the gelled tissue

**Step 7. Antibody Stripping**

1. Once tissue samples have been imaged, transfer the sample into a closed container (e.g., 6-well plate, conical tube) and add Softening buffer (with BME) to fully immerse the sample (e.g., 1x1 cm gel, cover with 3-5 ml buffer)

2. Incubate samples for two (2) hours at 70°C with gentle shaking (ensuring samples do not dry out).

3. Once the stripping incubation is finished, samples shrink to a size of approximate ~1.3x expansion factor.

4. Wash samples for five (5) times with 1x PBS for at least five (5) min each time.

*Times will be longer with thicker tissues.

5. Transfer sample into a 6-well plate and repeat **Step 5. Immunostaining Post-Decrowding** for another round of antibodies and **Step 6. Imaging** for imaging with the new round of antibodies.

*You can repeat “stripping and staining” as many rounds as needed to test markers of interest.

**Of note, we recommend keeping one channel constant or as a reference for all rounds. This constant or reference channel can be used for registration between rounds. For example, for FFPE human samples we found that using DAPI or staining with a goat anti-H3 histone antibody as the constant channel yielded excellent results. For example, if you have mouse, rabbit and chicken antibodies, you can do 3x3=9 different targets + 1 for DAPI or nuclear H3 histone, to achieve multiplexed imaging. However, you can choose your constant channel as your experiment dictates, just remembering that you will need a channel with enough similar features to ensure adequate registration between immunostaining rounds.

***After each round of stripping and staining, the gels shrink, and prior to imaging they require expanding back to ~4x expansion factor.

**Expansion Factor Considerations**

If the user wishes to calculate the expansion factor to relate the post-expansion physical units’ size to the pre-expansion biological unit’s size, they can consider the following two methods:

*Pre-expansion DAPI staining*

1. Following **Step 1** and prior to **Step 2,** stain the pre-expansion (native) tissues with 2 µg/mL of DAPI in 1x PBS for 30 min at RT.

2. Cover the tissue section with aqueous mounting media and take a pre-expansion image.

3. The expansion factor and thus, the biological units of length can be established later by measuring identical features from the pre-expansion tissue with the post-expansion tissue to calculate the expansion factor.


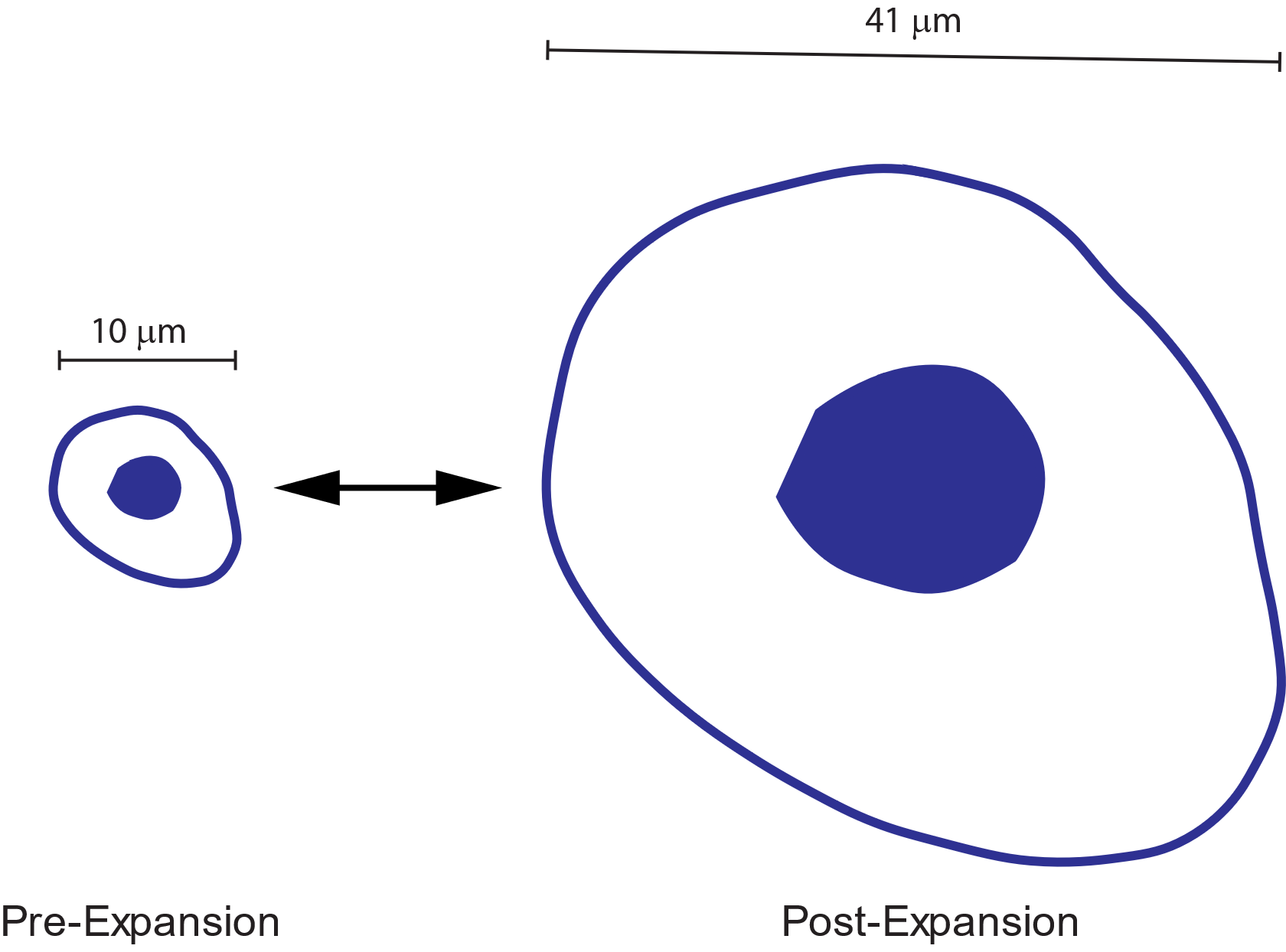


**Figure 8. Pre- and Post-Expansion DAPI Staining for Expansion Factor Calculation**

*Pre-expansion Gel Measurement*

1. Obtain pre-expansion measurements using a measurement tool (e.g., a ruler) to measure the gel prior to placing in the softening buffer.

2. Obtain post-expansion measurements using the same measurement tool but this time measure the expanded gel to calculate the expansion factor.

**Step 1 Extra. PRE-EXPANSION STAINING**

*Antigen retrieval*

1. After drying at the end of **Step 1**, place the slide in a humidified container and add Softening buffer to ensure the tissue is completely covered by buffer.

2. Incubate for 1 hour at room temperature in Softening buffer inside a humidified container.

*Here we incubated in Softening buffer for antigen retrieval to then perform pre-expansion staining and thus mimic the chemical conditions that the tissue undergoes following decrowding in the gel format. However, the user can use other methods of antigen retrieval if they so wish (e.g., citric acid with microwave incubation).

3. Remove excess Softening buffer from the slide and wash five (5) times with 1x PBS for 3 min each time.

*Pre-expansion antibody staining*

Here we proceed with primary and secondary antibody staining using the same conditions, antibodies, concentrations and buffers as in **Step 5**.

*Primary Antibody Staining*

1. Using a hydrophobic pen, create a layer surrounding the tissue (**Figure 9**).

2. In a humidified chamber add 200-500 µL of MAXblock™ Blocking Medium to make sure the tissue is completely covered with Blocking Medium and ensuring Blocking Medium is not overflowing over the hydrophobic layer and incubate for 1 hour at 37°C.

3. Remove excess MAXblock™ Blocking Medium and wash three (3) times with 1 mL each time using MAXwash™ buffer for 3 min each at RT.

4. Prepare primary antibody solution by diluting primary antibody in MAXbind™ Staining buffer.

5. Perform pre-expansion (i.e., pre-decrowding) immunostaining by adding 100-300 µL of primary antibody solution to make sure the tissue is completely covered with antibody staining solution and ensuring antibody staining solution is not overflowing over the hydrophobic layer and incubate tissue samples at 37°C for 1 hour, at RT for 2.5 hours or at 4°C overnight.

6. Remove excess primary antibody solution and wash three (3) times with 1 mL each time using MAXwash™ buffer for 3 min each at RT.


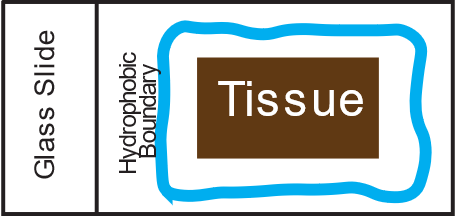


**Figure 9. Pre-expansion staining hydrophobic layer preparation**

*Secondary Antibody Staining*

7. Prepare secondary antibody solutions by diluting secondary antibody in MAXbind™ Staining buffer.

*If using a nuclear stain (e.g., DAPI), staining can be done by incorporating nuclear stain into the secondary antibody solution.

8. Incubate the tissue samples in 100-300 µL of secondary antibody solution using the same conditions applied for primary antibodies.

9. Remove excess secondary antibody solution and wash three (3) times with 1 mL each time using MAXwash™ buffer for 3 min each at RT and then transfer into a new container with 1x PBS.

10. Gently air-dry the slide and using Kim wipe, remove excess PBS for less than 1 min

11. Use a blade to scrape off the hydrophobic layer thoroughly.

12. Then add VectaShield (aqueous) mounting medium (2-3 drops) on the tissue and cover tissue with a #1 cover slip making sure there are no air bubbles trapped.

13. Add a drop of nail polish at each end so it covers both the end of the coverslip and a portion of the slide to help keep the coverslip without moving while imaging.

11. At this stage tissues are ready for imaging at the pre-expanded state or storage at 4°C in low light conditions to minimize quenching and drying out of tissues.

*Suggested incubation periods and temperatures are given as a guide only. It is recommended that the user optimize these parameters for use in their own experiment based on antibodies and tissue thickness. It has been our experience that with a large range of antibodies in tissues of this thickness, incubation times of 1-2 hours seem sufficient to get adequate staining.

**Antibody concentrations are antibody specific and will require optimization based on the antibody. It has been our experience that we start with the recommended concentrations by the vendor and then concentrate the solution as needed.

12. Once imaging has been completed, the user can submerge the tissue slide into a container with 1x PBS for 10 min at RT with gentle shaking.

13. Using a blade, gently remove the cover slip while in the PBS solution and once removed, wash slide in 1x PBS three (3) times for 3 min each.

14. Now tissues are ready to proceed with **Step 2** above.
